# Ultrasound-induced cavitation renders prostate cancer cells susceptible to hyperthermia: Analysis of potential cellular and molecular mechanisms

**DOI:** 10.3389/fgene.2023.1122758

**Published:** 2023-04-19

**Authors:** Shaonan Hu, Xinrui Zhang, Andreas Melzer, Lisa Landgraf

**Affiliations:** ^1^ Innovation Center Computer Assisted Surgery (ICCAS), University of Leipzig, Leipzig, Germany; ^2^ Institute for Medical Science and Technology (IMSaT), University of Dundee, Dundee, United Kingdom

**Keywords:** focused ultrasound, cavitation, hyperthermia, combination, mechanisms, prostate cancer

## Abstract

**Background:** Focused ultrasound (FUS) has become an important non-invasive therapy for prostate tumor ablation *via* thermal effects in the clinic. The cavitation effect induced by FUS is applied for histotripsy, support drug delivery, and the induction of blood vessel destruction for cancer therapy. Numerous studies report that cavitation-induced sonoporation could provoke multiple anti-proliferative effects on cancer cells. Therefore, cavitation alone or in combination with thermal treatment is of great interest but research in this field is inadequate.

**Methods:** Human prostate cancer cells (LNCap and PC-3) were exposed to 40 s cavitation using a FUS system, followed by water bath hyperthermia (HT). The clonogenic assay, WST-1 assay, and Transwell^®^ invasion assay, respectively, were used to assess cancer cell clonogenic survival, metabolic activity, and invasion potential. Fluorescence microscopy using propidium iodide (PI) as a probe of cell membrane integrity was used to identify sonoporation. The H2A.X assay and Nicoletti test were conducted in the mechanism investigation to detect DNA double-strand breaks (DSBs) and cell cycle arrest. Immunofluorescence microscopy and flow cytometry were performed to determine the distribution and expression of 5α-reductase (SRD5A).

**Results:** Short FUS shots with cavitation **(**FUS-Cav) in combination with HT resulted in, respectively, a 2.2, 2.3, and 2.8-fold decrease (LNCap) and a 2.0, 1.5, and 1.6-fold decrease (PC-3) in the clonogenic survival, cell invasiveness and metabolic activity of prostate cancer cells when compared to HT alone. FUS-Cav immediately induced sonoporation in 61.7% of LNCap cells, and the combination treatment led to a 1.4 (LNCap) and 1.6-fold (PC-3) increase in the number of DSBs compared to HT alone. Meanwhile, the combination therapy resulted in 26.68% of LNCap and 31.70% of PC-3 with cell cycle arrest in the Sub-G1 phase and 35.37% of PC-3 with cell cycle arrest in the G2/M phase. Additionally, the treatment of FUS-Cav combined with HT block the androgen receptor (AR) signal pathway by reducing the relative Type I 5α-reductase (SRD5A1) level to 38.28 ± 3.76% in LNCap cells, and decreasing the relative Type III 5α-reductase 3 (SRD5A3) level to 22.87 ± 4.88% in PC-3 cells, in contrast, the relative SRD5A level in untreated groups was set to 100%.

**Conclusion:** FUS-induced cavitation increases the effects of HT by interrupting cancer cell membranes, inducing the DSBs and cell cycle arrest, and blocking the AR signal pathway of the prostate cancer cells, with the potential to be a promising adjuvant therapy in prostate cancer treatment.

## Introduction

The mechanism of focused ultrasound or high-intensity focused ultrasound (FUS/HIFU) for medical applications is based on thermal and mechanical effects (Gourevich et al., 2013). In current clinical practice, HIFU-induced thermal ablation (at a temperature above 55°C) of the targeted tissue has been approved for the clinical treatment of uterine myomas, prostate diseases, bone metastasis-related pain, essential tremor, and Parkinson’s disease, and the techniques of magnetic resonance (MR) or ultrasound (US) imaging are used to anatomical guide the FUS waves to the target and simultaneously enable therapy control (Siedek et al., 2019). Currently, further applications of FUS e.g., blood-brain barrier opening, immune stimulation, and neuromodulation are in the preclinical and clinical research phases. More biological and physical understanding is required for the investigation of FUS application in cancer therapy.

As one of the most crucial mechanical effects caused by FUS/HIFU, cavitation is described as the linear or non-linear oscillation of small vapor-filled cavities in the effects of expansion and compression cycles traveling through a medium in an acoustic field (Izadifar et al., 2019). Stable oscillations of small vapor-filled cavities at low acoustic pressures induce micro-streaming around cavitation nuclei and increase mass transmission through micromixing and convection (Wiggins and Ottino, 2004). This effect is named stable cavitation, which is applied for the induction of cell sonoporation and to support drug delivery. At high acoustic pressures, the small-sized vapor-filled cavities will expand rapidly over a few acoustic cycles and collapse violently. The phenomenon is termed inertial cavitation (Wiggins and Ottino, 2004), during which the generation of shock waves and liquid microjets are applied for histotripsy (Schade et al., 2012) and induction of anti-vascular (Daecher et al., 2017) effects. Hydrophones (Lai et al., 2006; Morris et al., 2009; Bull et al., 2011; Maxwell et al., 2013; Lo et al., 2014) were utilized to investigate cavitation dose quantification previously. Lo et al. (2014) a needle hydrophone to measure and control the cavitation events that occurred in a 24-well plate. The results showed that accurate, stable, and repeatable cavitation levels could be obtained using the hydrophone method. In contrast to the needle hydrophone, the fiber-optic hydrophone (FOH) with a thin optic fiber sensor was able to be located inside the 96-well plate, allowing low interference to the acoustic field and more accurate determination of cavitation dose for the limited space (Bull et al., 2011). A lot of prior research manifested that the hydrophone technique could also be utilized to determine the cavitation activity within *ex vivo* tissues or tissue-mimicking phantom, and the FOH sensor showed an advantage of the ease of positioning within the tissues or phantom and higher spatial sensitivity to cavitation occurring within samples (Lai et al., 2006; Morris et al., 2009; Bull et al., 2011; Maxwell et al., 2013). The technique of passive cavitation detection was also reported to determine the cavitation activity precisely *in vivo*.

Thermal ablation is currently the most commercially available FUS application in the clinic, with several devices approved by the FDA for clinical use and a total of 374,812 patients treated with HIFU thermal ablation by 2020 [FUS foundation]. In the clinical context of HIFU treatment, cavitation is so far a problem and is avoided during clinical thermal ablation with existing HIFU systems since it can interfere with magnetic resonance imaging (MRI) thermometry and is difficult to forecast and manage throughout the treatment process (Izadifar et al., 2019). However, this does not imply that cavitation is an undesirable event for the treatment of tumors; in fact, histotripsy—the non-invasive mechanical destruction of diseased tissue using the cavitation effect has been clinically accomplished (Schuster et al., 2018). The non-thermal characteristics of cavitation overcome all the drawbacks of thermal effects, including the heat sink effect, lack of predictability of margins, and thermal spread, etc. (Xu et al., 2021). In addition to histotripsy, cavitation is also used in drug delivery, blood–brain barrier opening, lithotripsy, and the induction of blood vessel destruction during cancer treatment. Thermal and cavitation effects are frequently concomitant during FUS therapy, where the cavitation effect is also one of the mechanisms for thermal effect generation (Farny et al., 2009; Izadifar et al., 2019). With the development of clinical cavitation detection techniques such as passive cavitation detection, active cavitation detection, and MRI techniques, precise detection and control of cavitation events for their clinical use is becoming possible. It is feasible that cavitation will no longer be considered a “useless and to be avoided” form in future HIFU therapeutic applications, but rather could synergistically work in combination with thermal effects in therapies. Because numerous studies reported anti-proliferative effects on cancer cells (e.g., cell apoptosis, cell-cycle arrest, and clonogenicity suppression) caused by cavitation-induced sonoporation (Miller and Dou, 2009; Karshafian et al., 2010; Zhong et al., 2011; Chen et al., 2013), making it a potential adjuvant therapy to sensitize cancer cells in combination treatment regimes.

Hyperthermia (HT) refers to the generation of moderate heat in a range of 40°C–47°C where treatment time varies between a few minutes to 1 h (Hurwitz and Stauffer, 2014). HT technology can be classified into whole-body HT, localized HT, and regional HT, which are regularly employed to treat solid tumors in deep tissue with an external heat source to kill cancer cells or suppress their proliferation (Hegyi et al., 2013; Peeken et al., 2017). State-of-the-art of HT techniques includes electromagnetic equipment such as radiofrequency and microwave, ultrasound-induced HT, and novel magnetic nanoparticle heating (Peeken et al., 2017). Compared to other techniques, FUS-induced HT shows the benefit of non-invasive tissue penetration, and allowance of beamforming as well as shaping for both superficial and deep HT treatment. The HT caused by FUS has also been reported to induce cancer cell apoptosis by triggering intracellular oxidative stress (Hildebrandt et al., 2002; Saliev et al., 2013). HT inhibited the repair of DNA damage induced by radiotherapy and enhanced tissue oxygenation by improving blood flow, thus boosting the radiotherapy’s cytotoxic effect (Song et al., 1997; Kampinga and Dikomey, 2001). HT was also reported to render cancer cells susceptible to chemotherapeutic drugs, accelerating tumor cell death. Thus, HT is generally used as adjuvant therapy to improve radiation or chemotherapy (Peeken et al., 2017). Additionally, HT induces changes in the tumor microenvironment and stimulation of immune response (Peeken et al., 2017). Due to the good linearity and temperature dependence, proton resonance frequency (PRF) shift MR thermometry is widely used in MRI-guided HIFU for non-invasive temperature monitoring inside the body by measuring the phase change resulting from temperature-induced PRF shift (Rieke and thermometry., 2011).

The AR signaling pathway plays a unique role in the development, functionality, and homeostasis of the prostate (Lonergan and Tindall, 2011). The conventional functions of the AR signaling pathway include modulation of lipid and protein biosynthesis and coordination of cell division, differentiation, proliferation, and apoptosis (Meehan and Sadar, 2003). Both testosterone (T) and dihydrotestosterone (DHT) can bind to the AR to activate the AR signaling pathway. The dissociation rate of the AR-DHT complex is much lower than the AR-T complex. Therefore, DHT is regarded as the primary ligand for binding with the AR due to the more stable AR-DHT complex (Wu et al., 2013). The binding of DHT to the AR promotes the dissociation of heat-shock proteins, and thereafter the AR-DHT complex is transferred into the cell nucleus to bind with androgen response elements and other complex response elements. By this time, the AR is trans-activated by the co-activators located on the DNA to modulate the transcription and expression of corresponding genes. Using various techniques, 146 to 517 genes and 44 proteins regulated by the AR signaling pathway have been detected in human prostate cancer cells (Meehan and Sadar, 2003). The AR signaling pathway is crucial to the initiation and development of prostate cancer. Maintaining of AR protein and activation of the AR signaling pathway are in every stage of prostate cancer, even after androgen deprivation therapy (Wu et al., 2013). The AR signaling pathway is indispensable for normal prostate development and function but also crucial for the initiation and progression of prostate cancer. DHT is responsible for activating the AR and is generated from testosterone (T) by the enzyme 5α-reductase (SRD5A), playing a vital role in the AR signaling pathway (Li et al., 2011). Three isozymes of 5α-reductase have been identified inside the human body till now. Type I and type III 5α-reductase (SRD5A1 and SRD5A3) were discovered to be correlated with DHT generation and AR activation in malignant prostate tumors (Uemura et al., 2008; Godoy et al., 2011), while type II (SRD5A2) is primarily expressed in normal prostate tissues (Thigpen et al., 1993; Chen et al., 1998). Immunofluorescence is a widely used technique to visualize the distribution of SRD5A in the cytoplasm and quantify the SRD5A level *via* flow cytometry.

In contrast, the effect of cavitation in combination with the thermal effect has not been sufficiently investigated till now. Our previous study (Hu et al., 2020) indicated that FUS-induced cavitation can improve the treatment outcome of HT (Hyperthermia, 45°C for 30 min) on prostate cancer cell PC-3, it is still necessary to explore the mechanism of the synergistic effects of the combination treatment of FUS-induced cavitation and HT. Therefore, in this study, we investigated the therapeutic effect of combination treatment on prostate cancer cell line LNCap and PC-3, as well as the mechanisms of enhanced efficacy by combination treatment.

## Materials and methods

### Prostate cancer cell lines and cell culture

The human prostate cancer cell lines PC-3 and LNCap were purchased from the European Collection of Authenticated Cell Cultures (ECACC, Salisbury, United Kingdom). PC-3 is a cell line established from bone metastasis of grade IV prostatic adenocarcinoma from a 62-year-old male Caucasian and cells were cultured in Ham’s F-12 K (Kaighn’s) medium (Gibco, Thermo Fisher Scientific, Germany). LNCap is a cell line isolated from metastasis at the left supraclavicular lymph node of a 50-year-old patient with a confirmed diagnosis of metastatic prostate carcinoma and cells were cultured in RPMI 1640 medium (Gibco, Thermo Fisher Scientific, Germany). All cell culture media were supplemented with 10% (v/v) fetal bovine serum (FBS, Gibco, Thermo Fisher Scientific, Dreieich, Germany), 100 U/mL penicillin, and 100 mg/mL streptomycin (Biochrom GmbH, Berlin, Germany) and all cultures were maintained at 37°C with 5% (v/v) CO_2_ in humidified air. Cell culture mediums were changed every 2–3 days. For sub-cultivation and experiments, cells were routinely washed with phosphate-buffered saline (PBS) without Ca^+^, Mg^+^, and phenol red (Biozym Scientific GmbH, Germany) and detached using trypsin/EDTA (Biozym Scientific GmbH, Germany). Cells were routinely tested for *mycoplasma*.

### FUS *in vitro* system

**FIGURE 1:**
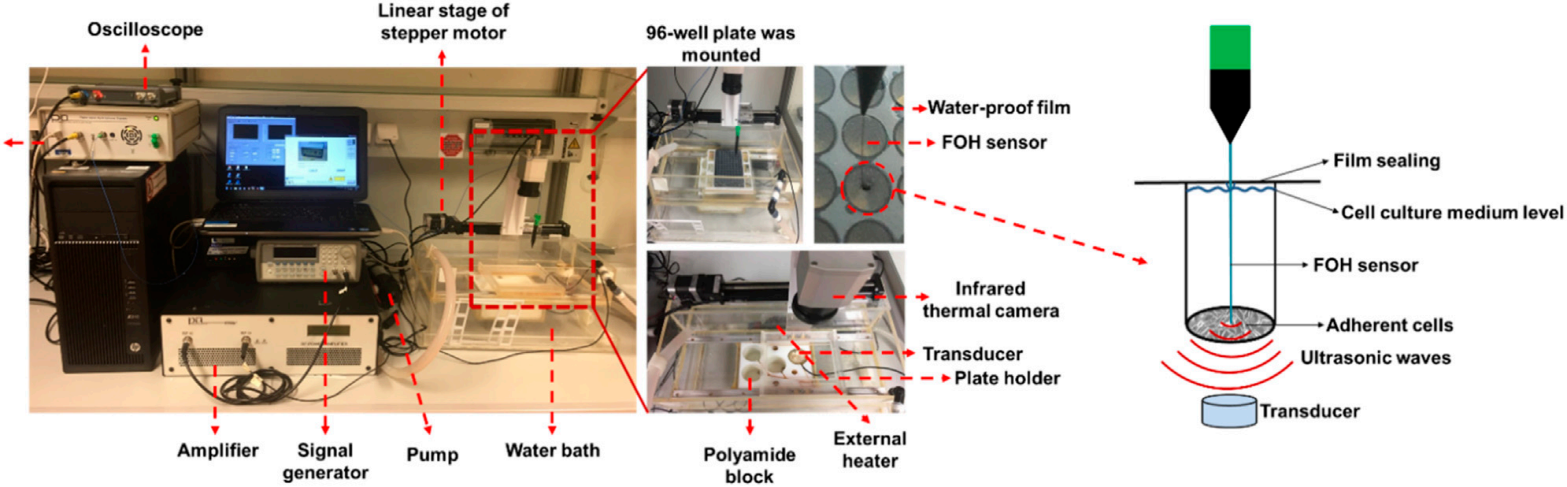
*In vitro* focused ultrasound (FUS) apparatus displaying experimental configuration for cavitation detection and FUS treatment. The FUS system is composed of a Perspex^®^ water bath compartment with pump and adjustable heater, a customized single-element FUS transducer at 1.467 MHz, a small polyamide block worked to detachably accommodate the transducer, a 3D-printed plate holder, and a stepper motor to move the 96-well plate. The continuous ultrasound waves were emitted from the transducer using the FUS signal generator and a radiofrequency power amplifier. A 96-well µclear plate was used for the FUS treatment of cells. To retain the cells in a sterile environment, the plate was protected with a water-proof film. A fiber-optic hydrophone system connected to an oscilloscope was utilized to calibrate the transducer and measure the dose of cavitation that occurs on the adherent cells. The right diagram shows the measurement position of the FOH sensor and the focused ultrasonic action site. A thermal camera was mounted to measure the temperature in real time. The LabView application was utilized to control the FUS treatment using a feedback loop to the thermal camera.

The *in vitro* FUS apparatus includes a Perspex^®^ water bath compartment, where the ultrasound source (transducer) and a 96-well cell culture plate located in a 3D-printed plate holder were placed. Degassed water was used as the transport medium that transferred ultrasonic waves to the cells, and also contributed to the creation of an environment with stable temperature during the treatment. A self-priming water pump (Lei Te Co., Ltd., Guangdong, China) was employed for the circulation of degassed water to prevent bubble formation beneath the plate from interfering with the FUS wave propagation. The circulating water passed through an external heater (Hydor, Salisbury, United Kingdom) to hold the water temperature at 34°C. A small polyamide block inside the water bath was used to detachably accommodate the customized single FUS transducer. The FUS transducer was made from Perspex^®^ tubes with geometrically-focused piezoceramic bowls positioned at the top of each tube with a frequency of 1.467 MHz. Adapted lengths of transducers were designed to precisely position the focus spot at the bottom of the 96-well plate. Various waveforms could be generated by a FUS signal generator (33120A, Agilent Technologies, Edinburgh, United Kingdom) and amplified by an A075 RF power amplifier (A075, Electronics and Innovation, Rochester, NY, United States). An X-slide linear stage connected to a programmable VXM motor controller and a NEMA 17 stepper motor (all VELMEX Inc., Bloomfield, NY, United States) were the main components of the motion system, which was used to move the 96-well plate for precise positioning of the focal regions at wells in different lines. Four starting positions of transducers on the polyamide block were alternated to sonicate selected wells in different columns of a 96-well plate. An infrared thermal camera (Optris PI450, Optris GmbH, Berlin, Germany) was mounted above the 96-well plates to monitor the real-time temperature in the wells during FUS treatments (Figure 1).

### FUS-Cav treatment of prostate cancer cells

Ultrasound penetrable 96-well plates (Greiner Bio-One, Frickenhausen, Germany) with µ-clear bottom were used for the FUS treatment of prostate cancer cells. Cancer cells were seeded at a concentration of 6,000–10,000 cells/well in 100 µL corresponding cell culture medium to reach 80%–100% of cell confluency at the desired time point post-treatment. The seeding was performed 24–48 h before treatment. The 96-well plates for culturing of LNCap cells were coated with 40 μL/cm^2^ poly-L-lysine solutions for 30 min at 37°C and washed twice with distilled water (Song and Khera, 2014) to improve the adherence of LNCap cells. Before sonication, up to 420 µL/well of cell culture medium was added in the wells and the 96-well plate was sealed with Titer Top^®^ film (Sigma-Aldrich, Munich, Germany). To separate the FUS-induced thermal and mechanical effects, an infrared thermal camera was mounted above the 96-well plate to monitor the real-time temperature in the wells during FUS treatments. When the temperature in the wells reached 39°C, the sonication stopped until the temperature decreased to the baseline of 34°C. In prior work (Hu et al., 2020), the FOH system was used to determine the cavitation dose at various focused acoustic intensities of 129, 344, 539, 1,136, and 1704 W/cm^2^. While accounting for the consistency and stability of the cavitation events at the focused acoustic field, a FUS treatment protocol (Hu et al., 2020) was designed to induce the cavitation on the adherent cancer cells: a segmental FUS treatment (FUS-Cav) at the acoustic intensity of 1136 W/cm^2^ and an active sonication duration of 40 s. The cavitation dose of FUS-Cav was 62.6 mV*s (stable cavitation dose: 16.33 ± 4.29 mV*s; inertial cavitation dose: 46.27 ± 17.17 mV*s). The active sonication duration of each segment was 0.86 s and the treatment duration was 126.7 s with a temperature of 36.50°C ± 1.53°C.

### HT treatment with water bath and protocol of combination treatment for prostate cancer cells

**FIGURE 2:**
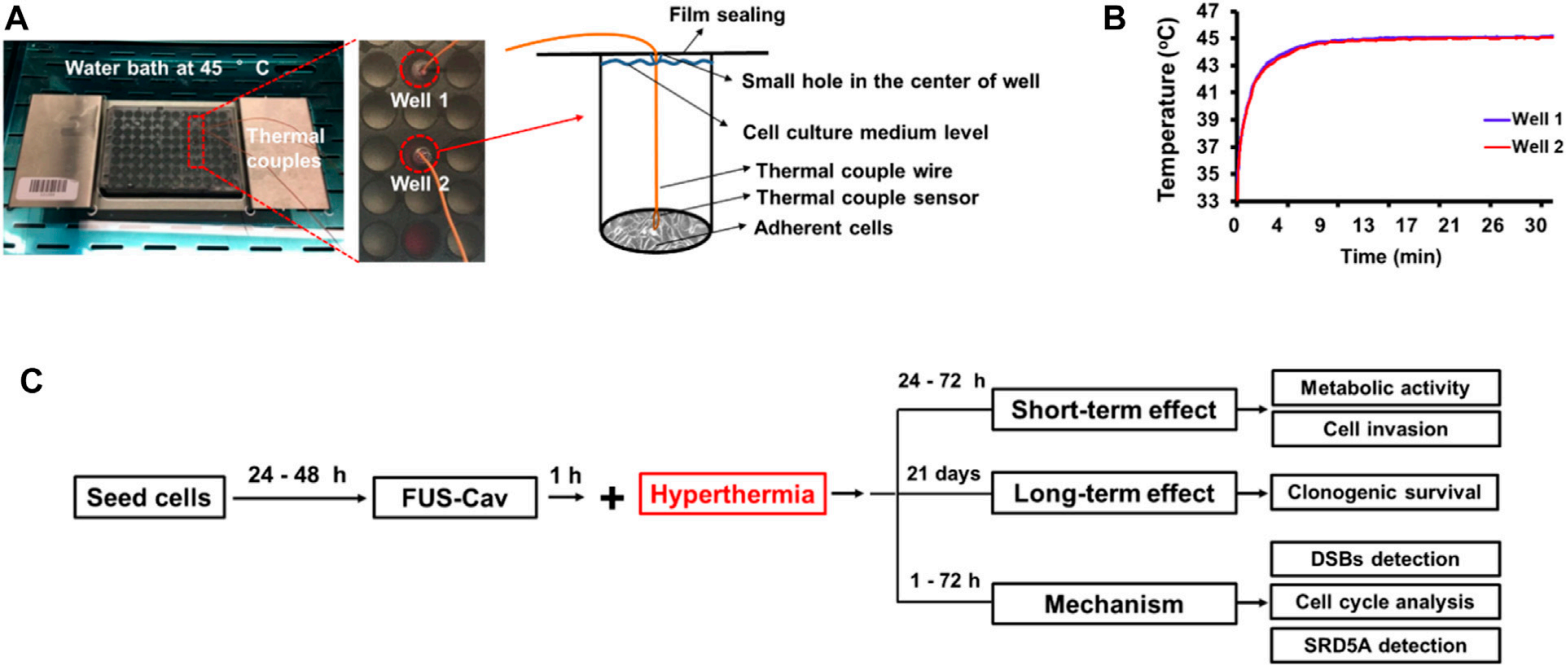
Experimental setup of water bath hyperthermia with 96-well plate at 45°C. **(A)** The real-time temperatures in two reference wells were measured with inserted thermal couples close to monolayer cells. **(B)** Temperature curves in two reference wells. **(C)** The timeline of the combined treatment and biological experimentation is depicted in the flow chart for the experimental plan.

In order to investigate whether cavitation treatment could benefit from HT, cancer cells were treated with conventional HT in a water bath (Perspex International, Lancashire, United Kingdom). Cells were seeded at a density of 6,000–10,000 cells/well in a 96-well plate to reach 80%–100% of cell confluency at the desired time point post-treatment. To maintain the sterile environment and prevent evaporation of the cell culture medium, 96-well plates were sealed with Titer Top^®^ films before water bath HT treatment and then carefully placed in a pre-warmed water bath. Type T PTFE-insulated Copper-Constantan precision fine wire thermocouples (diameter 0.07 mm, Pico Technology, St Neots, United Kingdom) were used to measure the temperature inside two reference wells (Figure 2A), and a Pico data logger was used to record real-time temperature and collect data (Figure 2B). Based on the literature (Cihoric et al., 2015) and preliminary experiments (Hu et al., 2020), water bath HT treatment was performed at the temperature of 45°C for 30 min. In order to examine the additive effects of FUS-Cav to HT, water bath HT treatment was performed 60 min after FUS-Cav. Measurements of metabolic activity and cell invasion were utilized to assess the short-term effect 24–48 h after treatment, and a clonogenic assay was used to evaluate the long-term effect 21 days after treatment. DSBs detection, cell cycle analysis, and measurement of SRD5A expression were conducted to investigate the underlying mechanism of FUS-Cav induced additive effect to HT treatment (Figure 2C).

### Evaluation of cell ability to reproduce with clonogenic assay

To examine the reproductive ability of a single cell in the long-term after different treatment regimes, clonogenic assay was performed according to the procedure reported by Franken et al. (Franken et al., 2006). Cell suspensions were harvested from 96-well plates with 100 μL trypsin/EDTA (×1) per well immediately after treatment and were seeded with a density of 500–1,000 cells/well into 6-well plates in triplicates. The 6-well plates were incubated for 21 days to allow colony formation, while the cell culture medium was changed twice per week. Colonies were gently rinsed with PBS twice before fixation with ice-cold methanol/acetone (1:1, V/V) for 5 min, afterward stained with 0.5% crystal violet solution in water for 30 min at room temperature and washed with deionized water to remove the unbound stain. Colonies in dried plates were scored if they exceeded a threshold number of 50 cells.

### Cell invasion assay

The potential of prostate cancer LNCap and PC-3 cells to migrate was evaluated by the *in vitro* Transwell^®^ invasion assay (Yu et al., 2013). The Transwell^®^ chamber system (Corning, New York, United States) consists of a Transwell^®^ insert mounted on the 24-well plate. The upper and lower chambers are divided by a polycarbonate porous membrane pre-coated with 100 μg/cm^2^ matrigel (Corning, New York, United States) at 37°C for 4 h. Cells were harvested from 96-well plates immediately post-treatment (FUS-Cav, HT, FUS-Cav + HT). Approximately 1 × 10^5^ cells were resuspended in 100 μL serum-free cell culture medium and seeded in the upper Transwell^®^ chamber. 600 µL medium was supplemented with 10% FBS as a chemo-attractant source in the lower chamber. After the incubation at 37°C for 48 h for the Transwell^®^ chamber system, a sterile cotton swab was used to remove the non-invaded cells on the upper surface of the polycarbonate porous membrane. The invaded cells on the lower surface of the membrane were fixed with 600 μL of methanol (Carl Roth, Karlsruhe, Germany) and stained with 0.1% crystal violet (Sigma-Aldrich, Munich, Germany) for 15 min at room temperature. The invaded cells were visualized with a microscope (Zeiss, Axioobserver), and five bright light images of random fields of view were taken at 200-fold magnification in each Transwell^®^ insert. The stained invaded cells were counted in ImageJ. All data were normalized to untreated control, which was set to 100%.

### Determination of impact on cell metabolic activity

To evaluate the short-term effects of the various treatments on the cellular metabolic activity of the human prostate cancer cell line LNCap and PC-3, the WST-1 assay was conducted 24, 48, and 72 h post-treatment. The cellular enzyme of mitochondrial dehydrogenases cleaved WST-1 reagent (tetrazolium salt) to formazan dye in the sample, quantification of formazan dye was directly correlated to the number of metabolically active cells in the culture medium. Based on the manufacturers’ instructions, the cell culture medium was discarded and cells were incubated with 100 µL fresh culture medium containing 10% WST-1 reagent (Carl Roth, Karlsruhe, Germany) in the 96-well plates at 37°C for 30 min. The absorbance of the formazan product was measured at 435 nm with a reference wavelength of 680 nm using a microplate reader (BioTek Instruments, Inc., Bad). All data were normalized to untreated control which was set as 100%.

### Detection of DSBs

To investigate the DSBs induced by FUS, HT or the combination treatment, γH2A.X assay was conducted 1 and 24 h after each treatment. Cell culture medium in 96-well plate was aspirated and discarded from each well, and cells were fixed with 4% formaldehyde for 10 min at 37°C. The cells were chilled on ice for 1 min, the fixative was discarded and cells were washed 3 times with ×1 PBS. The 90% methanol was added to each well to permeabilize cells on ice for 30 min, and cells were washed 3 times with ×1 phosphate-buffered saline (PBS) again. Non-specific bindings of antibodies were blocked with blocking buffer (0.5% bovine serum albumin solution (BSA, Cell Signalling Technology, Danvers, MA, United States, 100 µL/well) in PBS) at room temperature for 10 min. Following removing the blocking buffer, cells were incubated with 50 µL/well phospho-histone H2A.X (Ser139) rabbit primary monoclonal antibody (#9718, Cell Signalling Technology, Danvers, MA, United States) at the concentration of 1:400 diluted with blocking buffer at room temperature for 1 h. Cells were washed 3 times with anti-body free blocking buffer, and incubated with 50 µL/well of fluorescently conjugated secondary antibodies (Anti-Rabbit IgG (H + L), F (ab')2 Fragment (Alexa Fluor^®^ 594 Conjugate); #8889, Cell Signalling Technology, Danvers, MA, United States) at the concentration of 1:1,000 diluted with blocking buffer for 30 min at room temperature in the dark. After the incubation, the secondary antibody solutions were discarded and cells were washed 3 times with blocking buffer. Cell nuclei were stained with the nuclear stain 4, 6-diamidino-2-phenylindole (Fluoromount-G™ including DAPI, Thermo Fisher Scientific, Darmstadt, Germany) for 5 min. The foci representing DSBs were visualized at ex 561/em 594 nm and cell nuclei were stained by DAPI at ex 358/em 461 nm using a fluorescence microscope with 400-fold magnification. Semi-quantitative analysis was performed to calculate the mean numbers of stained foci per cell nucleus by the ImageJ software. All nuclei were counted in each image (80–120 nuclei).

### Cell cycle analysis detection

Nicoletti assay was performed 72 h after each treatment to explore the impact of FUS, HT or the combination treatment on cell cycle phase distribution and apoptosis-induced DNA fragmentation. After the trypsinization with 100 µL trypsin/EDTA per well, cell suspensions were harvested to the 1.5 mL microtube from a 96-well plate, cells were washed twice with PBS and fixed using 70% ethanol overnight at –20°C. Afterward, cells were washed twice with PBS again and incubated at 37°C for 20 min, with 60 µL RNaseA solution (Sigma-Aldrich GmbH, Munich, Germany) at a concentration of 0.1 mg/mL diluted with PBS. Next, the propidium iodide solution (PI, Sigma-Aldrich GmbH, Munich, Germany) at a concentration of 50 μg/mL in PBS was used to stain the DNA content of cells at 4°C for 5 min and cells were analyzed by flow cytometry (AttuneNxT, Thermo Fisher Scientific, Darmstadt, Germany).

### Visualization of SRD5A distribution

The impact of FUS-induced cavitation and HT on the SRD5A enzyme in prostate cancer cell lines was investigated using immunofluorescence microscopy. Cells in the 96-well plate were incubated at 37°C for 24 h after treatments. Cells in a 96-well plate were fixed on ice with 100 µL/well of 4% formaldehyde (Carl Roth, Karlsruhe, Germany) in PBS for 15 min, and the cells were permeabilized with 0.1% Triton^®^ X-100 (Carl Roth, Karlsruhe, Germany) in PBS for 10 min. Non-specific bindings of the antibodies were blocked by the blocking buffer (4% FBS in PBS) for 1 h at room temperature. Cells were incubated overnight with blocking buffer containing 2 μg/mL 5α-reductase-1 primary antibody (Anti-SRD5A1 antibody produced in rabbit; Sigma-Aldrich, Munich, Germany) or 5-reductase-3 primary antibody (Anti-SRD5A3 antibody produced in rabbit; Sigma-Aldrich, Munich, Germany) at 4°C. After washing four times with PBS, cells were incubated with 2 μg/mL secondary antibodies (Anti-Rabbit IgG (H + L), F (ab')2 Fragment (Alexa Fluor^®^ 594 Conjugate); #8889, Cell Signalling Technology, Danvers, MA, United States) in blocking buffer for 3 h at room temperature in the dark. The secondary antibody was removed, and the cell nuclei were stained with the nuclear stain 4, 6-diamidino-2-phenylindole (DAPI) for 5 min. Immunofluorescence in the absence of primary antibodies was used as the negative control. The expressions of SRD5A1 and SRD5A3 proteins labeled by Alexa Fluor^®^ 594 were visualized at ex 561/em 594 nm and cell nuclei were stained by DAPI at ex 358/em 461 nm using a fluorescence microscope with 400-fold magnification.

### Quantification for the reduction of SRD5A proteins with flow cytometry

Flow cytometry was performed to quantify the SRD5A positive cells. Cell suspensions were harvested in a 1.5 mL microtube from a 96-well plate 24 h incubation after treatments. Cell supernatants were discarded after centrifuging at 2000 rpm, for 5 min. The cells were fixed on ice with 4% formaldehyde solution in PBS for 15 min and permeabilized with 0.1% Triton ^®^ X-100 in PBS for 10 min. The non-specific binding of antibodies was blocked with a blocking buffer at room temperature for 1 h. The cells were incubated with 2 μg/mL 5α-reductase-1 primary antibody (Anti-SRD5A1 antibody produced in rabbit) or 5α-reductase-3 primary antibody (Anti-SRD5A3 antibody produced in rabbit) dissolved in blocking buffer overnight at 4°C. Next, cells were washed four times with PBS and incubated with 2 μg/mL secondary antibodies (Anti-Rabbit IgG (H + L), F (ab')2 Fragment (Alexa Fluor^®^ 594 Conjugate)) in a blocking buffer for 3 h at room temperature in the dark. Samples without incubation of primary antibody were used as the background control, and cells with higher fluorescence intensity than the background group were the fluorescent dye positive cells, the percentage of which indicated the overall SRD5A level 24 h after all treatments. Cell doublets and debris were excluded from the analysis of forward-scattered light (FSC) versus side-scattered light (SSC) using flow cytometry (AttuneNxT, Thermo Fisher Scientific, Darmstadt, Germany). Analysis of the percent dye positive cells was performed on at least 20,000 single cells. All data were normalized to untreated control which was set to 100%.

### Cell sonoporation investigation

Sonoporation is defined as the recoverable perforation of the cell membrane created by FUS-induced stable cavitation. In order to investigate this phenomenon on cell membranes post-FUS exposure, PI was employed as a probe of cell membrane integrity in this study. PI cannot penetrate the intact cell membranes of living cells (Van Wamel et al., 2006) but is permeant to the sonoprated cells due to the perforation of the cell membranes. After the cell membrane is restored from sonoporation, PI remains inside the cells and stains the cell nucleus. CellMask™ Green Plasma Membrane Stain (Thermo Fisher Scientific, Darmstadt, Germany) was used to visualize cell membranes in this experiment. Cancer cells were seeded at a density of 5,000 cells/well in an ultrasound-permeable 96-well plate with a µ-clear bottom 24 h before sonication. Cells were treated with the FUS-Cav protocol as described above for 40 s in the cell culture medium, and the cells were gently washed with 100 μL of PBS. PI (Cayman Chemical, Ann Arbor, Michigan, United States) at a final concentration of 1 μg/mL and CellMask™ at a final concentration of 5 μg/mL were added to the cell culture medium before or 30 min after FUS-Cav treatment. PI-stained cell nuclei were visualized immediately at the excitation/emission at 535/617 nm, and cell plasma membranes were stained with CellMask™ at ex 522/em 535 nm using a fluorescence microscope and ZEN2.3 software. Since the focused spot of FUS exactly covers one well bottom of the 96-well plate, five random-field fluorescence images were taken at 200-fold magnification. Cells stained with PI and CellMask™ (approximately 150 cells in each field) were calculated to quantify the percentage of PI-positive cells.

### Statistical analysis

The results of all measurements, including the survival fraction (clonogenic assay), cell invasion (transwell assay), metabolic activity (WST-1 assay), DNA double-strand breaks (H2A.X assay), cell cycle (Nicoletti assay), SRD5A visualization and quantification (immunofluorescence assay with microscopy and flow cytometry), and sonoporation efficiency (PI uptake assay), were expressed as mean ± SEM (Standard Error of the mean) of three independent experiments in two replicates. One-way ANOVA and the Tukey test for *post hoc* analysis were used to evaluate the significant differences between the mean values in any two groups. The non-parametric Mann-Whitney test was used in the statistical analysis of the clonogenic survival data in SPSS statistical software version 24. Statistical significance was defined as a *p*-value ≤ 0.05.

## Results

### FUS-Cav increases the effects of HT by reducing the clonogenic survival of prostate cancer cells

**FIGURE 3:**
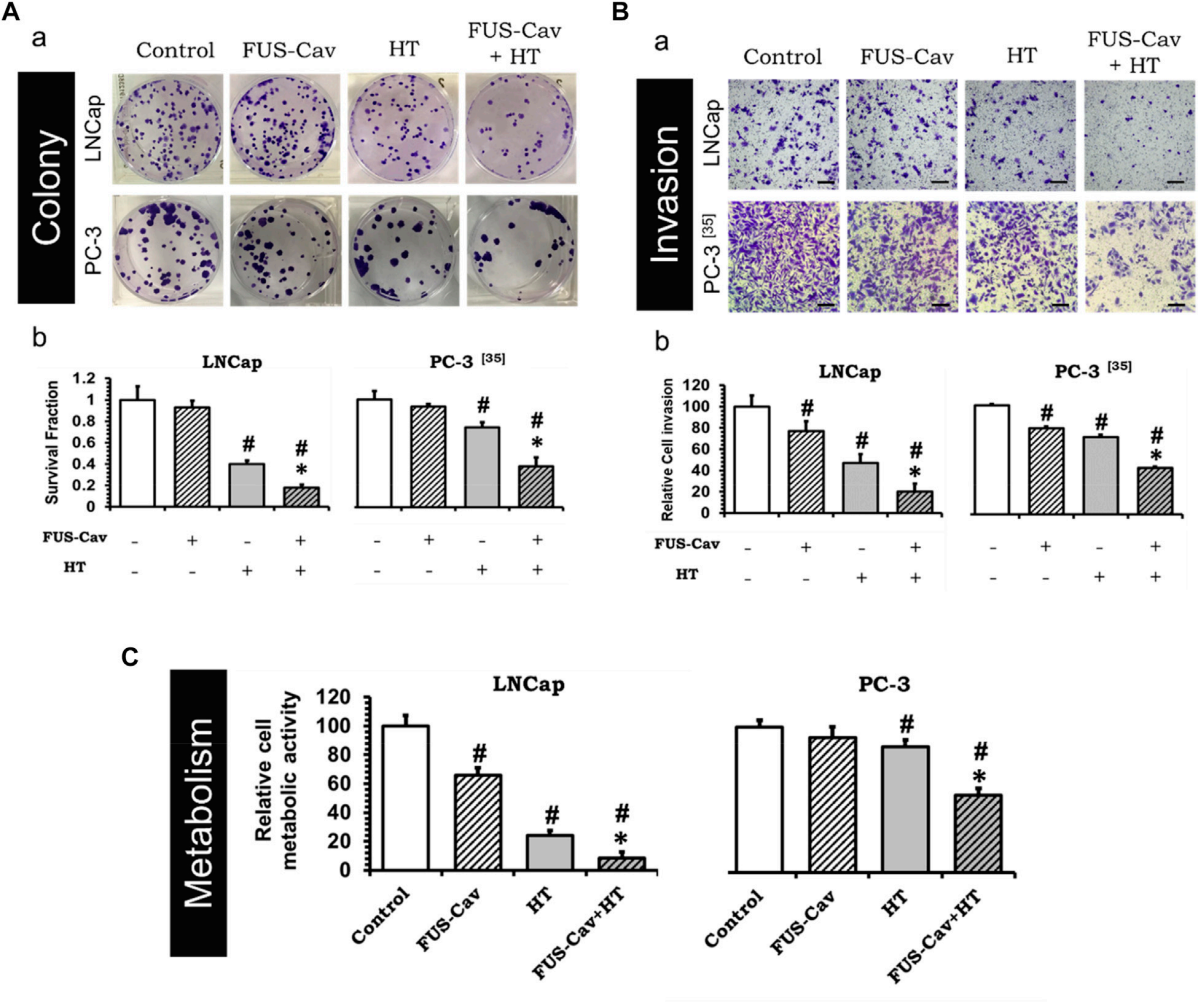
FUS-Cav (1136 W/cm^2^, 40 s) demonstrated additive effect to HT (45°C, 30 min). **(A)** a: Representative images of colony formation in LNCap and PC-3 cells 21 days post-treatment. Cell survival fraction was calculated as the counted colonies divided by the product of the seeding number and the plating efficiency. b: Cell survival fraction diagrams of LNCap and PC-3 (Hu et al., 2020) cells, suggesting the ability of a single cell to grow into a colony after various treatments. **(B)** a: Representative microscopy images of Transwell^®^ assay in LNCap and PC-3 (Hu et al., 2020) cells 48 h post-treatment. Scale bar = 100 μm. b: Relative cell invasion of LNCap and PC-3 (Hu et al., 2020) cells derived from semi-quantitative analysis of the Transwell^®^ assay revealing cell invasive potential 48 h post-treatment. **(C)** Relative cellular metabolic activity was detected by WST-1 assay 72 h (for LNCap) or 48 h (for PC-3) post-treatment. Data were normalized to untreated control, which was set to 100%, and relative values are presented as mean ± SEM, *n* = 6, *significantly different from HT (*p* ≤ 0.05), ^**#**^significantly different from control (*p* ≤ 0.05).

To investigate whether the treatment of short FUS-induced cavitation owns the long-term additional benefits to HT, the clonogenic survival fraction (SF) of cancer cells was assessed based on the number of cell colonies (Figure 3Aa) post-treatment. Although FUS-Cav alone only showed a limited impact on the clonogenic survival of LNCap cells, a significant decrease in SF was observed after the combination of FUS-Cav and HT in comparison to all single treatment groups. The effect on clonogenic survival following HT (SF: 0.40 ± 0.030) was enhanced by combining FUS-Cav (FUS-Cav + HT) revealing a 2.2-fold reduction of SF to 0.18 ± 0.028 (Figure 3Ab). For another prostate cancer cell line PC-3, the long-term increasing effects of FUS-Cav to HT were reported in our previous research (Hu et al., 2020): the combination of FUS-Cav and HT results in a 2-fold (SF: 0.37 ± 0.080) decrease of clonogenic survival compared to HT alone (0.74 ± 0.042) (Figure 3Ab).

### FUS-Cav supports HT to diminish cell potential to invade and metabolic activity of prostate cancer cells

The short-term additive effects induced by FUS-Cv to HT were investigated *via* the evaluation of LNCap and PC-3 cell invasion (Figure 3Ba) 48 h post-treatment. Similar to the results of long-term effects evaluation, FUS-Cav treatment also showed a significant additive effect on HT in the short term. The FUS-Cav alone leads to a significant loss of cell potential to invade (relative cell invasion of FUS-Cav: 77.19 ± 9.25%) compared to control, and the cavitation also enhanced the effects of HT, reducing the relative cell invasion from 46.87 ± 8.42% (HT) to 20.30 ± 7.16% (FUS-Cav + HT) (Figure 3Bb). Comparable effects were observed in the PC-3 cell line as described in detail previously (Hu et al., 2020): the combination treatment of FUS-Cav and HT leads to a significant decline of cell relative invasion (46.67 ± 1.17%) in comparison to single HT (70.73 ± 2.14%) (Figure 3Bb).

Another approach to evaluate the short-term additive effects induced by FUS-Cv to HT was WST-1 assay, which was performed 24, 48, and 72 h after each treatment. For both prostate cancer cell lines, the relative cell metabolic activities in the combination groups (FUS-Cav + HT) were significantly decreased at each incubation time point compared to the single treatment groups. Nevertheless, the magnitude of the decline varies depending on the incubation time. For LNCap cells, the relative cell metabolic activities were reduced from 97.62 ± 16.54% (24 h), 28.17 ± 4.38% (48 h), and 24.00 ± 3.60% (72 h) in the single treatments (HT alone) to 52.60 ± 13.43% (24 h), 19.08 ± 10.57% (48 h), and 8.54 ± 4.34% (72 h) in the combination groups (FUS- Cav + HT), respectively (Figure 3C). And for PC-3 cells (Hu et al., 2020), the relative cell metabolic activities declined from 81.60 ± 7.92% (24 h), 86.26 ± 4.84% (48 h), and 78.38 ± 10.56% (72 h) in the single HT treatments to 64.09 ± 1.84% (24 h), 53.20 ± 21.49% (48 h), and 66.76 ± 11.28% (72 h) in the combination treatment of FUS-Cav and HT (Figure 3C).

### Boost of DSBs by the combination treatment of FUS-Cav and HT

**FIGURE 4:**
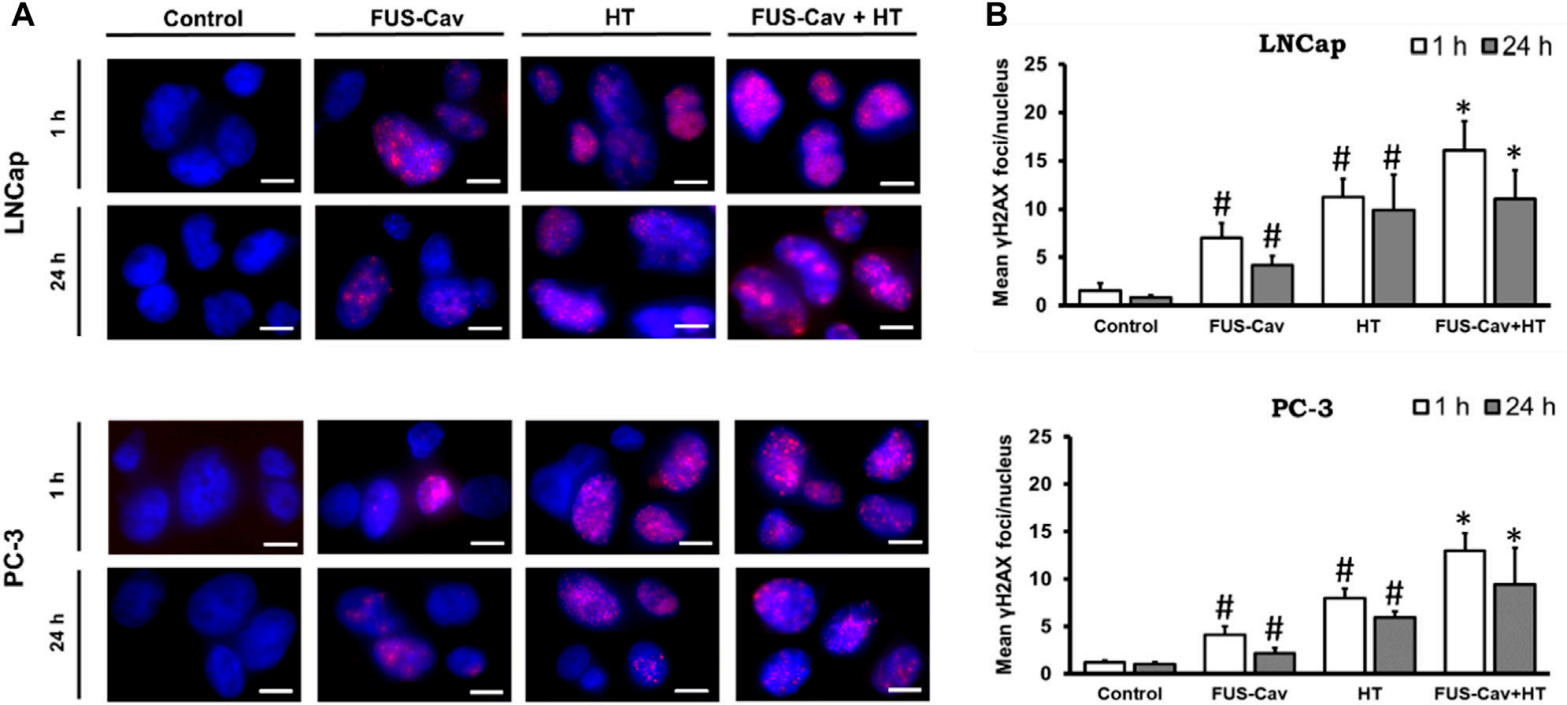
DSBs increased when FUS and HT were combined as opposed to when they were administered separately. **(A)** H2A.X foci (Alexaflour568 red) in the cell nucleus (blue) after single FUS or single HT is shown in representative microscopic fluorescence images, with the combined treatment (FUS + HT) displaying a greater amount of H2A.X foci than every single treatment. Scale bar = 10 µm. **(B)** Semi-quantitative analysis of H2A.X foci at 1- and 24- hours post-treatment. Data were presented as mean ± SEM, *n* = 6, *significantly different from HT (*p* ≤ 0.05), #significantly different from control (*p* ≤ 0.05).

The formation of γH2A.X represented an early cellular response event against DSB and was used as a biomarker to monitor DNA damage and repair. The effects of FUS-induced cavitation and its combined therapy with HT on the mechanism of sensing and repairing DNA damage were evaluated by counting the number of γH2A.X foci at 1 and 24 h after treatment in this study. Representative fluorescent microscopy images of stained γH2A.X foci (Figure 4A) display low levels of stained initial (1 h) and residual (24 h) foci in both cell lines in the untreated groups, with foci numbers ranging only from 0.85 to 1.53 foci/nuclei. Higher numbers of initial and residual γH2A.X foci were observed in the LNCap cell nucleus (initial foci number: 7.03 foci/nuclei; residual foci number: 4.19 foci/nuclei) than PC-3 cell (initial foci number: 4.15 foci/nuclei; residual foci number: 2.18 foci/nuclei) after a single FUS-Cav treatment (Figure 4B). The number of initial foci detected in LNCap (1.4-fold) and PC-3 (1.6-fold) cells was significantly enhanced after combining FUS and HT (LNCap: 16.07 foci/nuclei; PC-3: 12.97 foci/nuclei) compared to HT alone (LNCap: 11.22 foci/nuclei; PC-3: 8.00 foci/nuclei) (Figure 4A). Residual lesions, defined as foci scoring 24 h after treatment, revealed a similar trend to the result of initial lesions, with a slight decline in the number of foci compared to initial lesions (Figure 4A). Hence the highest numbers of stained initial and residual foci in both cell lines were observed in the combined group (FUS-Cav + HT).

### Sub-G1 apoptosis and G2/M phase arrest induced by the combination treatment of FUS-Cav and HT

**FIGURE 5:**
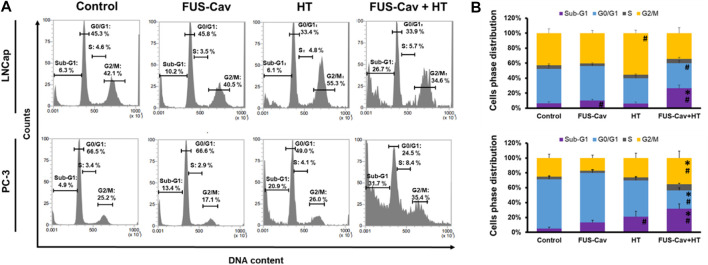
The combination of FUS-Cav and HT impacts the cell cycle phase in apoptotic sub-G1 and G2/M. **(A)** Representative flow cytometry results exhibit the percentage of cells in various cell cycles following each treatment. **(B)** Graphical columns show the cell cycle distribution of LNCap and PC-3 cells 48 h post-treatment. The count of cells in each cell cycle was presented as a percentage of the total cell amount. Data were presented as mean ± SEM, *n* = 6, *significantly different from HT (*p* ≤ 0.05), #significantly different from control (*p* ≤ 0.05).

Flow cytometry was performed to analyze the regulation of cell cycle distribution and sub-G1 fraction representing apoptotic cells 24 h after the combination treatment of FUS-Cav and HT (Figure 5A). For LNCap cells, single HT treatment showed a significant accumulation of cells in the G2/M phase compared to the untreated control but no change in the Sub-G1 phase. Interestingly, single FUS-Cav (10.22 ± 1.60) led to a significantly increased percentage of cells in the Sub-G1 phase compared to untreated cells (6.28 ± 1.94) (Figure 5B). And the combination treatment of FUS-Cav and HT exhibited a dramatic accumulation of cells in Sub-G1, the percentage of cells arrested in the Sub-G1 phase increased from 6.28 ± 1.94% in untreated cells and 6.13 ± 1.93% in the HT group to 26.68 ± 4.38%. This was accompanied by a concomitant decline in the percentage of cells in the G0/G1 phase (Figure 5B). For PC-3 cells, a significant enhancement of the percentage of cells in the Sub-G1 phase was observed in the treatment of HT combined with FUS-Cav (FUS-Cav + HT: 31.7 ± 6.40%) compared to single treatment and untreated control (FUS-Cav: 13.35 ± 2.75%; HT: 20.88 ± 7.31%; Untreated control: 4.89 ± 2.00%). Notice that there was a significant increase in the percentage of cells at the G2/M phase from 26.02 ± 6.47% in HT alone to 35.37 ± 9.30% in HT combined with FUS-Cav, accompanied by a decrease in the percentage of cells in the G0/G1 phase (Figure 5B).

### FUS-Cav enhances the effects of HT by inhibiting the SRD5A protein level in prostate cancer cell lines

**FIGURE 6:**
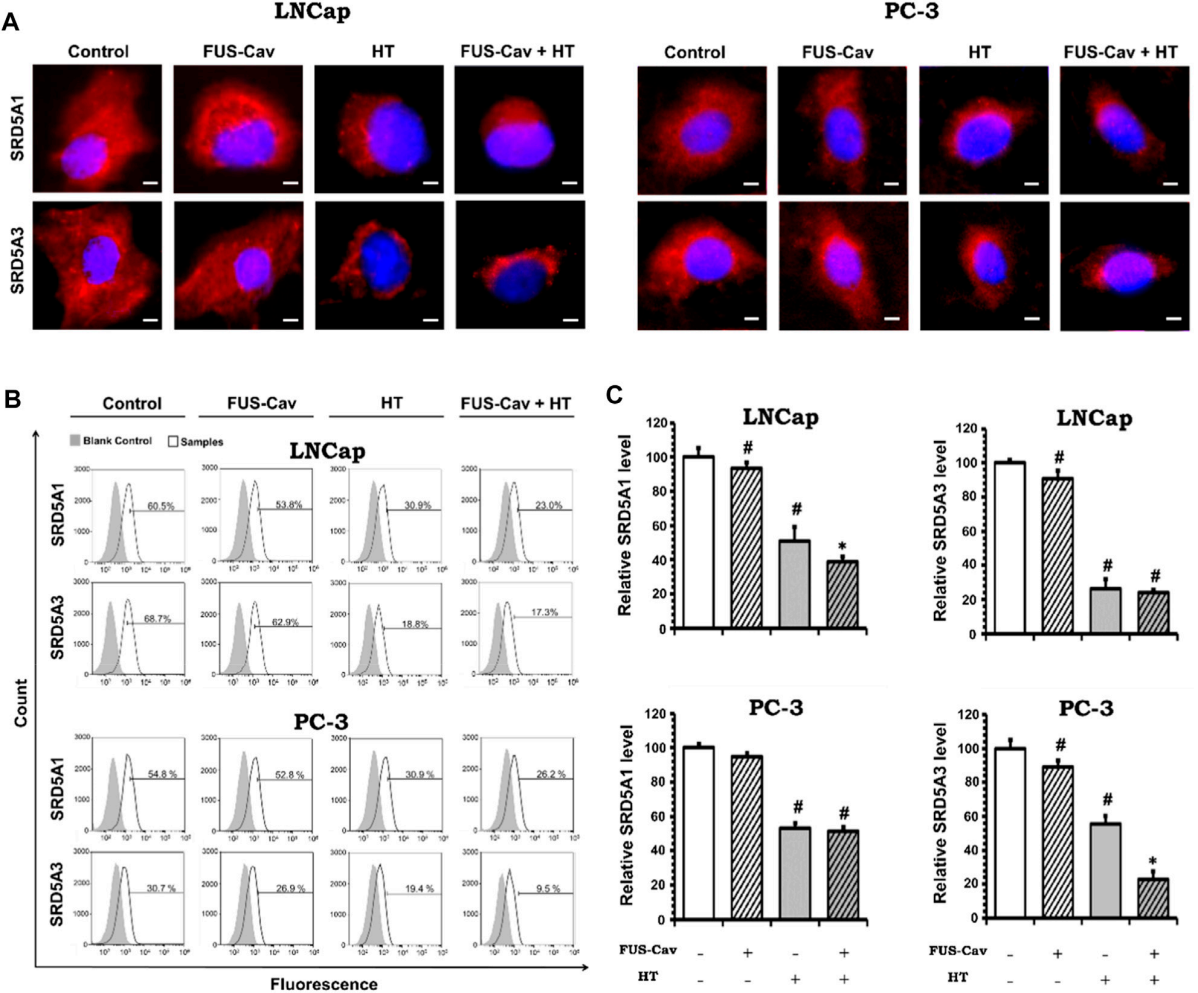
Effects of FUS-Cav to water bath HT decreasing the SRD5A distribution and expression were associated with cancer cell type. **(A)** Representative fluorescence microscopy images for LNCap and PC-3 cells showing the distribution of SRD5A1 and SRD5A3 proteins in the cytoplasm 24 h post-treatment. Scale bar = 5 µm. **(B)** Flow cytometry results exhibit the percentage of immunofluorescence-positive cells in all collected LNCap and PC-3 cells for analysis: histograms of immunofluorescence-positive cells showing the SRD5A level (percentage of fluorescence-positive cells indicated in each plot) after each treatment. The fluorescence-negative cells immune-stained in the absence of a primary antibody were set as the background control. **(C)** Statistical results of quantitative analysis with flow cytometry indicate SRD5A1 and SRD5A3 levels in LNCap and PC-3 cells 24 h post-treatment. Data were normalized to untreated control, which was set to 100%, and relative values are presented as mean ± SEM, *n* = 6, *significantly different from HT (*p* ≤ 0.05), #significantly different from control (*p* ≤ 0.05).

After various treatment regimes, fluorescent microscopy was performed to visualize the subcellular localization of immunofluorescence-tagged SRD5A1 and SRD5A3 in the prostate cancer PC-3 and LNCap cell lines. Figure 6A shows the Alexa Fluor^®^ 594-tagged SRD5A1 and SRD5A3 protein, and the fluorescence is distributed diffusely throughout the cytoplasm. In the fluorescent microscopy images for both PC-3 and LNCap cell lines, the distribution of SRD5A1 and SRD5A3 proteins were downregulated 24 h post single treatment of water bath HT. The effects of water bath HT treatment on reducing the SRD5A level were seemingly enlarged by adding short FUS-Cav treatment in terms of the visualization of SRD5A protein distributions (Figure 6A). In order to quantify the percentage of dye-positive cells in the total number of cells collected for analysis, flow cytometry was performed for the fluorescence-activated cell sorting after each treatment. Figure 6B shows the percentage of cells with immunofluorescence-tagged SRD5A1 or SRD5A3 protein for PC-3 and LNCap cell lines 24 h post-treatment. Figure 6C demonstrates the relative level of these two isozymes to untreated control in both cell lines after various treatments.

For the LNCap cell line, the relative SRD5A1 level was slightly decreased to 91.19 ± 2.98% by single treatment of FUS-Cav compared to untreated control (100 ± 5.39%). Nevertheless, FUS-Cav strengthened the impacts of combination treatment of FUS-Cav + HT, significantly reducing the relative SRD5A1 level from 51.21 ± 6.47% (HT) to 38.28 ± 3.76% (FUS-Cav + HT). FUS-Cav treatment alone significantly reduced the SRD5A3 level to 87.93 ± 4.58% compared to the untreated control. However, the combination treatment of FUS-Cav and water bath HT resulted in a decrease in SRD5A3 level from 26.78 5.03% (HT) to 23.32 1.76% (FUS-Cav + HT), indicating that FUS-Cav had no significant additive effects on water bath HT (Figure 6C).

For the PC-3 cell line, the relative SRD5A1 level was significantly diminished to 52.94 ± 2.84% by the single treatment of water bath HT compared to the untreated sample (100 ± 2.22%). Single FUS-Cav did not show significant suppressive effects on the expression of SRD5A1 compared to untreated control, and the combinatory treatment of FUS-Cav and water bath HT was not able to significantly reduce the SRD5A1 level compared to single treatment of water bath HT as well, indicating FUS-Cav had no additive effects to water bath HT suppressing the SRD5A1 expression. Single treatment of FUS-Cav induced a slight decline in SRD5A3 expression. The relative SRD5A3 level was significantly decreased by the combinatory treatments to 22.87 ± 4.88% (FUS-Cav + HT) compared to single water bath HT (55.70 ± 4.70%), denoting a significant additive effect of FUS-Cav to water bath HT in reducing the SRD5A3 expression (Figure 6C).

### FUS-Cav induces sonoporation in prostate cancer cells

**FIGURE 7:**
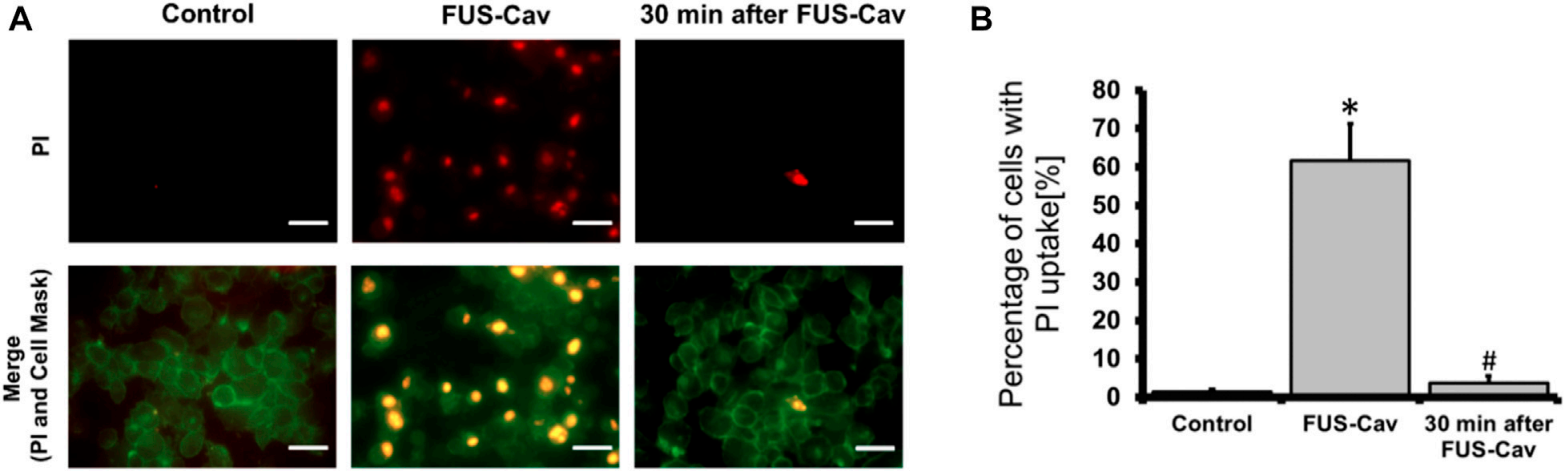
FUS-Cav induced sonoporation in LNCap cells. **(A)** Representative fluorescence microscopy images for LNCap cells showing an increase in red PI fluorescence during FUS-Cav; red: PI-stained cell nucleus; green: CellMask™ stained cell membranes, scale bar = 30 µm. **(B)** Semi-quantitative analysis of PI-positive percentage representing the quantity of sonoporated LNCap cells. Data were normalized to total cell number as 100% and relative values presented as mean ± SEM, *n* = 6, *significantly different from control (*p* ≤ 0.05), #significantly different from FUS-Cav (*p* ≤ 0.05).

The sonoporation phenomenon induced by FUS-Cav was investigated exemplarily in prostate cancer cells. PI, which initially cannot penetrate the intact cell membrane, can pass through the pores created temporarily in the cell membrane by sonoporation and stain the cell nucleus. Therefore, PI staining was employed as an indicator to explore the phenomenon of sonoporation, and CellMask™ staining was used to visualize cell membranes (Figure 7A). Since the area of one well of the 96-well plate was exactly covered by the dimension of the focal field, the fluorescent images were taken randomly in the visualized view of the wells. Compared to untreated control, FUS-Cav treatment immediately led to an enhanced percentage of LNCap cells with a PI stained nucleus (Figure 7A) suggesting the occurrence of sonoporation. In the semi-quantitative results (Figure 7B), the percentage of PI-positive cells was significantly enhanced to 61.7% in LNCap cells immediately after exposure to FUS-Cav, and only 3.7% PI-positive cells were observed 30 min post-treatment suggesting the recovery of sonoporation in LNCap cells. The sonoporation effects induced by FUS-Cav in PC-3 cell line were reported in previous research (Hu et al., 2020): FUS-Cav treatment induced sonoporation effects (PI-positive) were observed in 49.9% of PC-3 cells, and the percentage of PI-positive cells is only 4% left 30 min after FUS-Cav indicating the resealing of cell membranes in PC-3 cells.

## Discussion

The mechanisms of FUS/HIFU in medical sectors are mostly separated into i) thermal and ii) mechanical effects., with the cavitation effects being the emphasis of research and application in mechanical effects. Clinically, the intense heat generated by HIFU is utilized non-invasively and accurately to ablate prostate tumors under the guidance of MRI (Zhang et al., 2021). FUS allows for targeted treatment, causing thermal tissue coagulation, necrosis, and heat shock by raising the temperature by 55°C–80°C (Bakavicius et al., 2022). The majority of preclinical applications of cavitation effects have been focused on drug delivery (Xia et al., 2021; Wang et al., 2022), histotripsy (Xu et al., 2021), lithotripsy and application of anti-vascular effects (Tung et al., 2011; Kwok et al., 2013; Daecher et al., 2017). Cavitation is not regarded as having a favorable impact in the clinical application of HIFU thermal ablation of prostate cancers due to the lack of relevant studies demonstrating the synergistic effect between the two concomitant FUS-induced mechanisms i.e., cavitation and thermal effects. A PCI (passive cavitation imaging) system is utilized to monitor the broadband emissions to avoid cavitation-induced inadvertent tissue injury (Izadifar et al., 2019). *In vitro*, a short FUS shot (with cavitation) can support standard hyperthermia (HT) to reduce cell clonogenicity, metabolic activity, and cell potential to invade in human prostate cancer (PC-3), glioblastoma (T98G), and head and neck cancer (FaDu) cells, according to our previous study (Hu et al., 2020). The mechanisms of treatment could be linked to cavitation-induced sonoporation, which has been shown to induce several anti-proliferative effects on cancer cells but no more molecular mechanisms have been identified (Feril and Kondo, 2004; Miller and Dou, 2009; Karshafian et al., 2010; Zhong et al., 2011; Chen et al., 2013; Saliev et al., 2013). In this study, the effect of FUS-induced cavitation in combination with HT in the treatment of prostate cancer was further investigated using another prostate cancer cell line LNCap, and the therapeutic mechanism was evaluated from the perspectives of cell membrane disruption, DSBs, cell cycle arrest, and inhibiting the AR signal pathway of prostate cancer cells.

**FIGURE 8:**
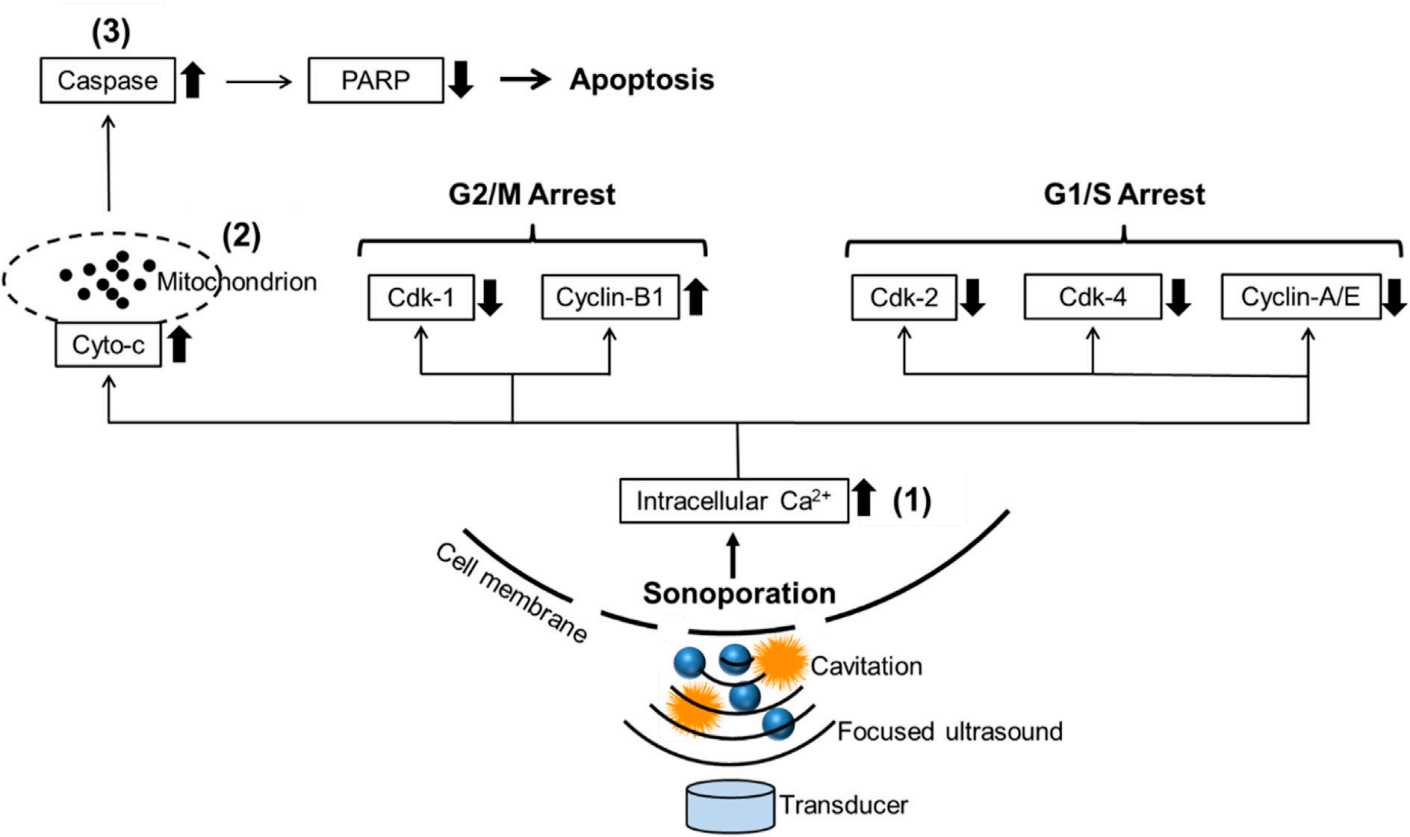
Schematic diagram reveals the potential mechanism of sonoporation-induced cell apoptosis and cell-cycle arrest. The transduction of intracellular signaling molecules involves 1) intracellular Ca^2+^ signaling system, 2) mitochondrion biology, and 3) apoptosis signaling pathway. Cyto-c: cytochrome c, PARP: polyadenosine diphosphate ribose polymerase, Cdk: Cyclin-dependent kinases. Adapted from (Zhong et al., 2011).

Currently, a lot of research about cavitation mainly focuses on its effects on cell membranes. Cavitation was reported to induce the deformation, damage, or sonoporation on the membrane of cancer cells and was usually used to deliver therapeutic agents (e.g., drugs or gene fragments) to targeted cells (Zolochevska et al., 2011; Wang et al., 2013; Yu et al., 2016). Sonoporation is a unique effect induced by FUS on cells: when the cavitation occurs near cell membranes, the extraction and contraction of the gas-filled cavities create the pores on the cell membrane temporarily (Tu and Yu, 2022). PI, a fluorescent dye with a molecular diameter of 0.8 nm, was used to investigate the sonoporation effect *in vitro* (Van Wamel et al., 2006). Pores created by sonoporation were reported to be 110 ± 40 nm in size, allowing PI molecules to pass through the cell membrane (Zhou et al., 2009). Sonoporated cells reseal the cell membrane within minutes after being exposed to ultrasound. However, the self-repairing of the cell membrane does not signify that sonoporation has no impact on cell survival. Papers are reporting the anti-proliferative effects of sonoporation or cavitation on cancer cells (Miller and Dou, 2009; Karshafian et al., 2010; Zhong et al., 2011; Chen et al., 2013). Sonoporation-induced apoptosis of human leukemia cells was associated with the decreased expression of polyadenosine diphosphate ribose polymerase (PARP) protein, which is a pro-apoptotic marker correlated to the impairment of DNA repair functionality (Figure 8). Sonoporation was also found to suppress the expression of a variety of checkpoint proteins such as cyclin and Cdk (cyclin dependent kinase) which play a vital role in cell cycle progression and prolong the DNA synthesis, thereby inducing cell cycle arrest in leukemia cells (Figure 8) (Zhong et al., 2011; Chen et al., 2013). Similarly, the percentages of sub-G1 phase in both sonoporated prostate cancer cell lines (apoptotic cells) (Plesca et al., 2008) increased significantly after treatment with FUS-induced cavitation compared to untreated groups, according to our findings. Sonoporation triggered cell cycle arrest on prostate cancer cells predominantly in the sub-G1 phase of apoptotic cells, whereas leukemia cells mostly in the G2/M and G1/S phases, implying that the cavitation-induced sonoporation effect on the cancer cell cycle arrest is cell type-dependent. Furthermore, when FUS-generated cavitation is combined with thermal effects (45°C for 30 min) that belongs to another mechanism of FUS, a substantial accumulation of cells in the Sub-G1 phase was observed in prostate cancer cells compared to a single treatment of HT or FUS-Cav. This is believed to be one of the potential mechanisms underlying the combined effects of HT treatment and FUS-induced cavitation on the inhibition of cell metabolic activity and clonogenic survival in prostate cancer cells *in vitro*.

DSBs are randomly repaired in the absence of an intact template copy, wherefore they are considered one of the most dangerous forms of DNA damage. Incorrect gene repair can result in genomic rearrangements (deletions, translocations, fusions of DNA, etc.), leading to cell death potentially (Furusawa et al., 2014). Previous research on the mechanical effects of ultrasound on cells focused mainly on the impairment of cell membrane permeability and the consequent triggering of several biochemical responses, such as cell cycle arrest caused by sonoporation as mentioned above, as well as apoptosis (Zhong et al., 2011; Chen et al., 2013). However, it has been demonstrated that ultrasonic can penetrate the nuclear territory deeply, leading to alterations in genes and protein expression while also enhancing macromolecular localization. Previously, it was shown that DSBs occurred in pure DNA solution irradiated by ultrasound (Kondo et al., 1985). A neutral comet test was recently employed by Yukihiro Furusawa et al. to confirm the occurrence of DSBs in various leukemia cell lines exposed to ultrasonic irradiation *in vitro*. DNA damage was not highly associated with apoptosis and was primarily induced by the cavitation effect rendered by ultrasound waves penetrating deep into the nuclear territory (Furusawa et al., 2014). The occurrence of DSBs after the FUS-Cav treatment on prostate cancer cell lines (PC-3 and LNCap) was confirmed by the detection of DSBs events using γH2A.X as a biomarker, and the occurrence of DSBs in the nuclei of both prostate cancer cell lines was significantly increased when FUS-Cav was combined with thermal therapy compared to any single treatment in our study. This is in line with the findings of Yukihiro Furusawa, who suggested that cavitation may be a worrying sign as it shakes the safety of ultrasound in diagnostic applications, but maximized DNA damage induced by FUS-induced thermal effect combined with cavitation may eventually lead to cell death, which is beneficial for cancer eradication potentially. And unquestionably advantageous for the use of therapeutic ultrasound (i.e., HIFU) to treat tumors.

Zhou et al. (2012), reported that approximately half of the sonoporated KHT C (mouse fibrosarcoma) cells could not maintain long-term cell survival after the ultrasound-mediated MBs. The potential impacts of ultrasound mechanical effects also include changes in cell ultrastructure, division ability, chromosomal and cytogenetic effects, and functions (Izadifar et al., 2017). In our previous research (Hu et al., 2020), sonoporation occurred in more PC-3 cells (49.9%) than FaDu cells (23.3%) immediately after short FUS shots with cavitation (FUS-Cav) revealing that PC-3 cells are more susceptible to cavitation, which might be the biophysical mechanism at the cellular level here leading to lower survival of PC-3 compared to FaDu cells in the combination treatments. The extents and types of anti-proliferative effects induced by sonoporation vary depending on cancer cell types (Miller and Dou, 2009; Karshafian et al., 2010; Zhong et al., 2011; Chen et al., 2013). In the current investigation, FUS-Cav was able to induce sonoporation in 61.7% of LNCap cells, and more than 49.9% of PC-3 cells. Our findings suggest that LNCap is more susceptible to cavitation than PC-3, and when cavitation is combined with thermal effects on LNCap, it inhibits cell metabolic activity, clonogenic survival, and invasion potential to a greater extent than PC-3. Although FUS-induced cavitation was proved to induce sonoporation in prostate cancer cell lines and head and neck cancer cell lines in our studies, the subsequent anti-proliferative effects (e.g., cancer cell apoptosis, cell-cycle arrest or prolong of DNA-synthesis) are still required to be clarified in future research and validated in animal models.

In our experiment, the treatment of water bath HT at 45°C for 30 min led to a significant reduction of cell invasion of prostate cancer cells LNCap and PC-3 compared to untreated control. Short FUS shots with cavitation (FUS-Cav) also demonstrated a significant suppressive effect on the invasion of prostate cancer cells, the pre-treatment to LNCap and PC-3 cells with FUS-Cav significantly expanded the impact of the subsequent HT treatment to inhibit cell invasion, exhibiting the additive effects of FUS-Cav to HT on the suppression of prostate cancer cell potential to invade in our study. In some other research, the activation of the phosphatidylinositol-3-kinase/AKT (PI3K/AKT) signaling pathway was discovered to be responsible for the migration and invasion of prostate cancer cells (Zhou et al., 2017). Ogata et al. (2005); Ogata et al. (2011) described that the inhibition of invasiveness of small cell lung cancer cell line A594 was induced by the downregulation of the matrix metalloproteinase (MMP)-2, which was attributed to the inactivation of the PI3K/AKT signaling pathway. HT was previously shown to inhibit cancer cell invasion *via* the downregulation of metastatic-related proteins, MMP-2 and MMP-9 (Xie et al., 2011). In numerous studies, the expression of MMP-2/9 has usually associated with the PI3K/AKT signaling pathway (Ogata et al., 2005; Adya et al., 2008; Chen et al., 2009; Zhu et al., 2019), the suppression of the PI3K/AKT signaling pathway in prostate cancer cells could result in the downregulation of MMP-2/9 (Chien et al., 2010). It has been previously reported that the cavitation effects were able to hinder the invasion and migration of PC-3 cells *via* downregulation of the MMP-2/9 (Wei et al., 2014). Based on the literature reports, HT as well as cavitation could inhibit the invasion of cancer cells in varying degrees by hindering cancer metastatic-related proteins MMPs. We assume that the combination of cavitation with HT carries the potential to reduce the expression of MMPs compared to single treatments, which is supposed to be the underlying mechanism of cavitation-induced additive effects to HT on the inhibition of the prostate cancer cell potential to invade. The inhibition of MMPs might be associated with the inactivation of the PI3K/AKT signaling pathway.

5α-reductase (SRD5A) proteins were discovered to be associated with dihydrotestosterone generation and activation of the AR signaling pathway in the prostate. SRD5A2 was the predominant form of 5α-reductase in healthy prostate tissue and benign prostatic diseases. SRD5A1 and SRD5A3 were the primary isozymes in the prostate cancer cells, and suppression of SRD5A1 and SRD5A3 protein levels was reported to be a promising alternative therapy to block the AR signaling pathway and inhibit the growth of malignant prostate tumors (Uemura et al., 2008; Godoy et al., 2011). In our experiment, a single FUS-Cav treatment demonstrated a minor inhibitory effect on the expression of SRD5A1 and SRD5A3 in prostate cancer cells, and the combination treatment of FUS-Cav and water bath HT led to a significant reduction of SRD5A protein compared to single HT, but the suppressive effects of combination treatment for an isoform of SRD5A are cell-type dependent. FUS with cavitation supported HT to reduce SRD5A1 level but had no impact on SRD5A3 in the LNCap cell line. In contrast, FUS-Cav combined with HT resulted in the decrease of SRD5A3 level compared to single HT, but no additive effect to HT treatment in inhibiting SRD5A1. Type I and III of SRD5A coexist in prostate cancer cells to boost the AR pathway and support prostate cancer initiation and progression, thus downregulating either SRD5A1 or SRD5A3 may restrict the AR pathway and further inhibit the growth of prostate cancer cells (Uemura et al., 2008; Godoy et al., 2011). SRD5A protein was significantly decreased by FUS-Cav + HT treatment in prostate cancer cells, which might result in the inactivation of the AR signaling pathway and a reduction in cell survival.

In clinical practice, the leading cause of prostate cancer-related death is cancer metastasis (Li et al., 2014). Patients with metastatic prostate cancer have a poor quality of life and usually suffer from urinary retention and bone pain (Kiljunen et al., 2015). Cancer cell migration is the crucial step for cancer progression to a metastatic state (Dirat et al., 2015). It is necessary to find an approach to reduce the potential of cancer cells to invade for slowing the progression of prostate cancer. From our studies, short FUS shots with cavitation sensitize prostate cancer LNCap and PC-3 cells to HT, inhibiting not only the short- and long-term survival as mentioned above but also the cell potential to invade. It provides a novel strategy combining the use of HIFU-induced cavitation and thermal effects to reduce the potential to spread the prostate cancer cells through the body leading to cancer metastasis. The potential of FUS-induced cavitation as an HT therapy sensitizer was demonstrated by using an *in vitro* cell culture model in our prior study (Hu et al., 2020). Biological analyses at the cellular and molecular levels (e.g., apoptosis, DNA damage, cell cycle, and AR pathway) were performed in the current study to uncover the underlying mechanisms of cavitation-induced additive effect on HT. It is also worthwhile to mention the limitation of the current study. The current findings obtained by *in vitro* experiments warrant being validated by *in vivo* experiments, where the ultrasound parameters should be optimized in accordance with the tissues' properties for ultrasound propagation. Microbubble administration should be taken into consideration to generate constrained cavitation effects in animals or humans with reduced acoustic intensities. However, it should be noted that our *in vitro* study provided the first evidence that FUS-Cav sensitizes cancer cells to HT and revealed its underlying mechanisms, thereby offering a solid foundation for future *in vivo* and clinical studies.

## Conclusion

Our findings demonstrated the additive effect of FUS-induced cavitation when it was applied together with moderate heating, which was displayed in a reduction of the metabolic activity, invasiveness, and clonogenic survival of LNCap cells (a cell line with lymph node metastasis from prostate cancer). Compared with PC-3 cells (a cell line with bone metastases from prostate cancer) investigated in our previous study, FUS-Cav showed a stronger additive effect on HT treatment for the LNCap cell line in this study. Our findings further imply that the cavitation-induced sonoporation may be connected to the additive effects of FUS on HT, with potential mechanisms including the induction of DSBs, cell cycle arrest, and blocking of the AR signaling pathway in prostate cancer cells.

## Data Availability

The raw data supporting the conclusion of this article will be made available by the authors, without undue reservation.

## Abbreviations

AR, androgen receptor; Cdk, cyclin-dependent kinases; cyto-c, cytochrome c; DAPI, 4, 6-diamidino-2-phenylindole; DHT, dihydrotestosterone; DSBs, DNA double-strand breaks; FBS, fetal bovine serum; FDA, food and drug administration; FOH, fiber-optic hydrophone; FUS, focused ultrasound; FUS-Cav, short FUS shots with cavitation; HIFU, high-intensity focused ultrasound; HT, hyperthermia; MMP, matrix metalloproteinase; MR, magnetic resonance; MRI, magnetic resonance imaging; PARP, polyadenosine diphosphate ribose polymerase; PBS, phosphate-buffered saline; SEM, standard error of the mean; SF, clonogenic survival fraction; SRD5A, 5α-reductase; SRD5A1, type I 5α-reductase; SRD5A2, type II 5α-reductase; SRD5A3, type III 5α-reductase; T, testosterone; US, ultrasound.
