# Supplementary figures and images for: Ultrasound-induced cavitation renders prostate cancer cells susceptible to hyperthermia: Analysis of potential cellular and molecular mechanisms
Source: Front Genet. 2023 Apr 19;14:1122758. doi: 10.3389/fgene.2023.1122758 (PMC10154534; doi:10.3389/fgene.2023.1122758)

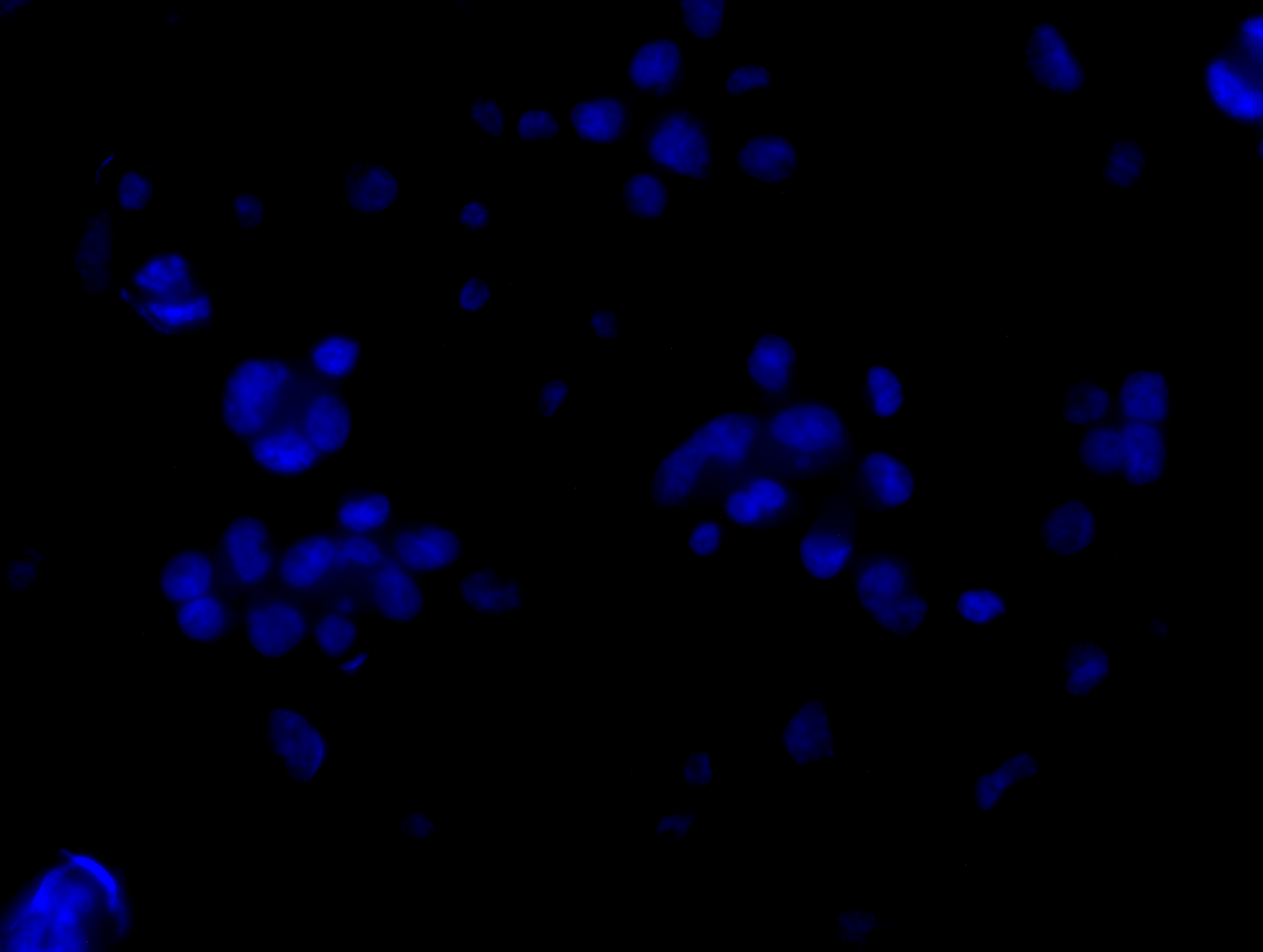

Supplement: Supplementary file 1 [file DataSheet3.ZIP › Figure 4a Microscopy images for LNCap and PC-3 cell DNA double strand breaks/Figure 4a Microscopy images for LNCap and PC-3 cell DNA double strand breaks LNCap 1h Control.tif]

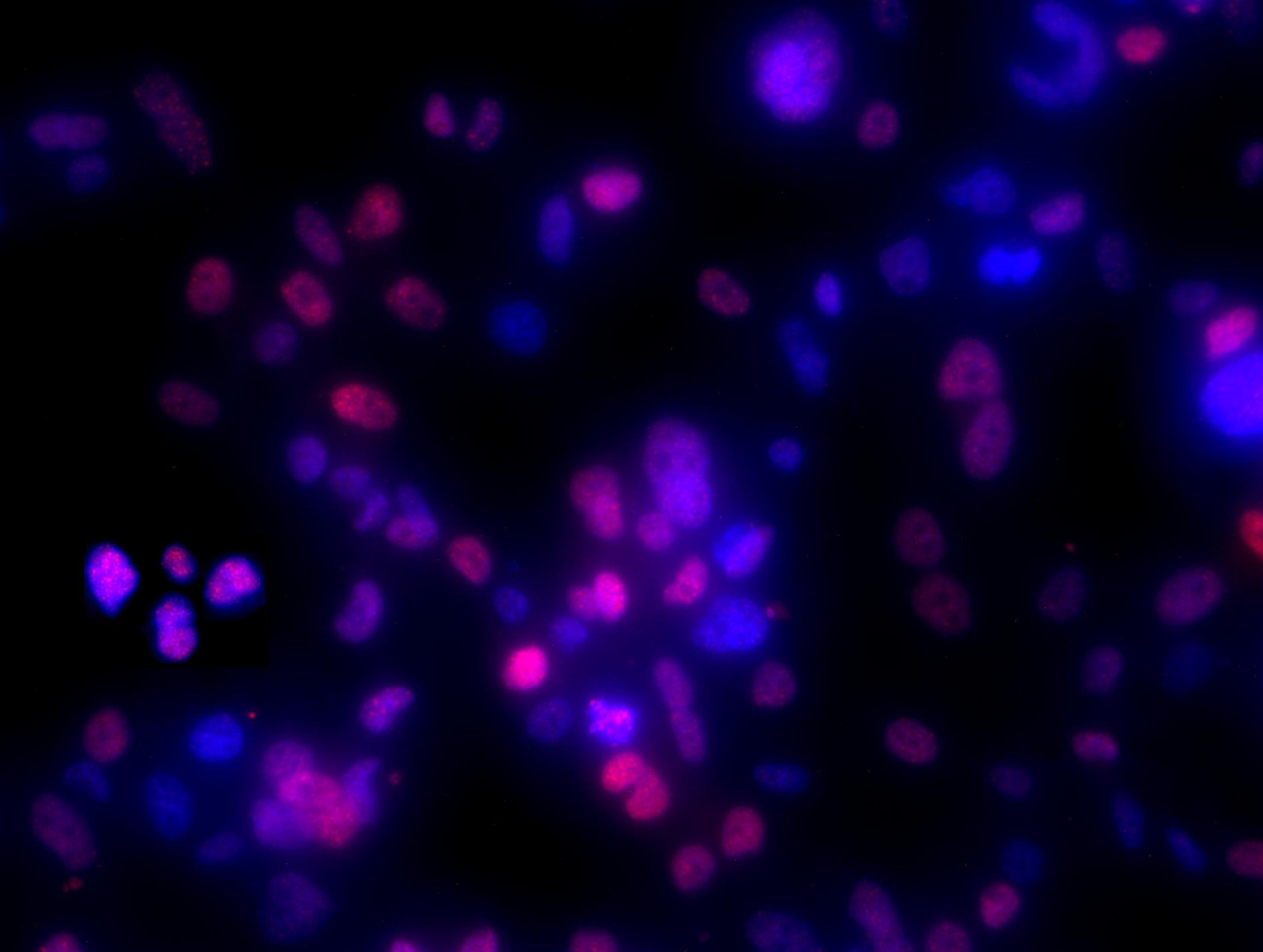

Supplement: Supplementary file 1 [file DataSheet3.ZIP › Figure 4a Microscopy images for LNCap and PC-3 cell DNA double strand breaks/Figure 4a Microscopy images for LNCap and PC-3 cell DNA double strand breaks LNCap 1h FUS-Cav + HT.tif]

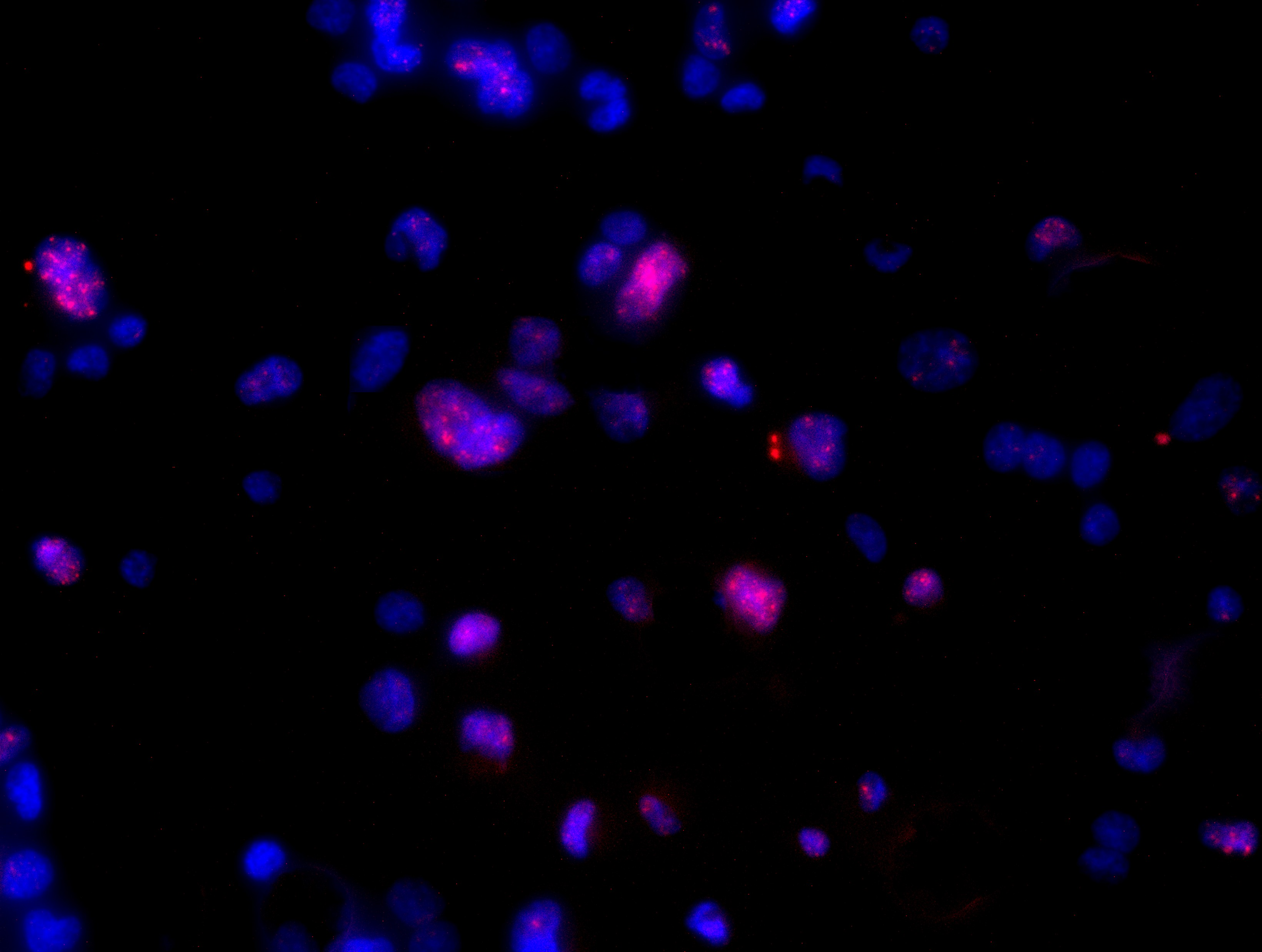

Supplement: Supplementary file 1 [file DataSheet3.ZIP › Figure 4a Microscopy images for LNCap and PC-3 cell DNA double strand breaks/Figure 4a Microscopy images for LNCap and PC-3 cell DNA double strand breaks LNCap 1h FUS-Cav.tif]

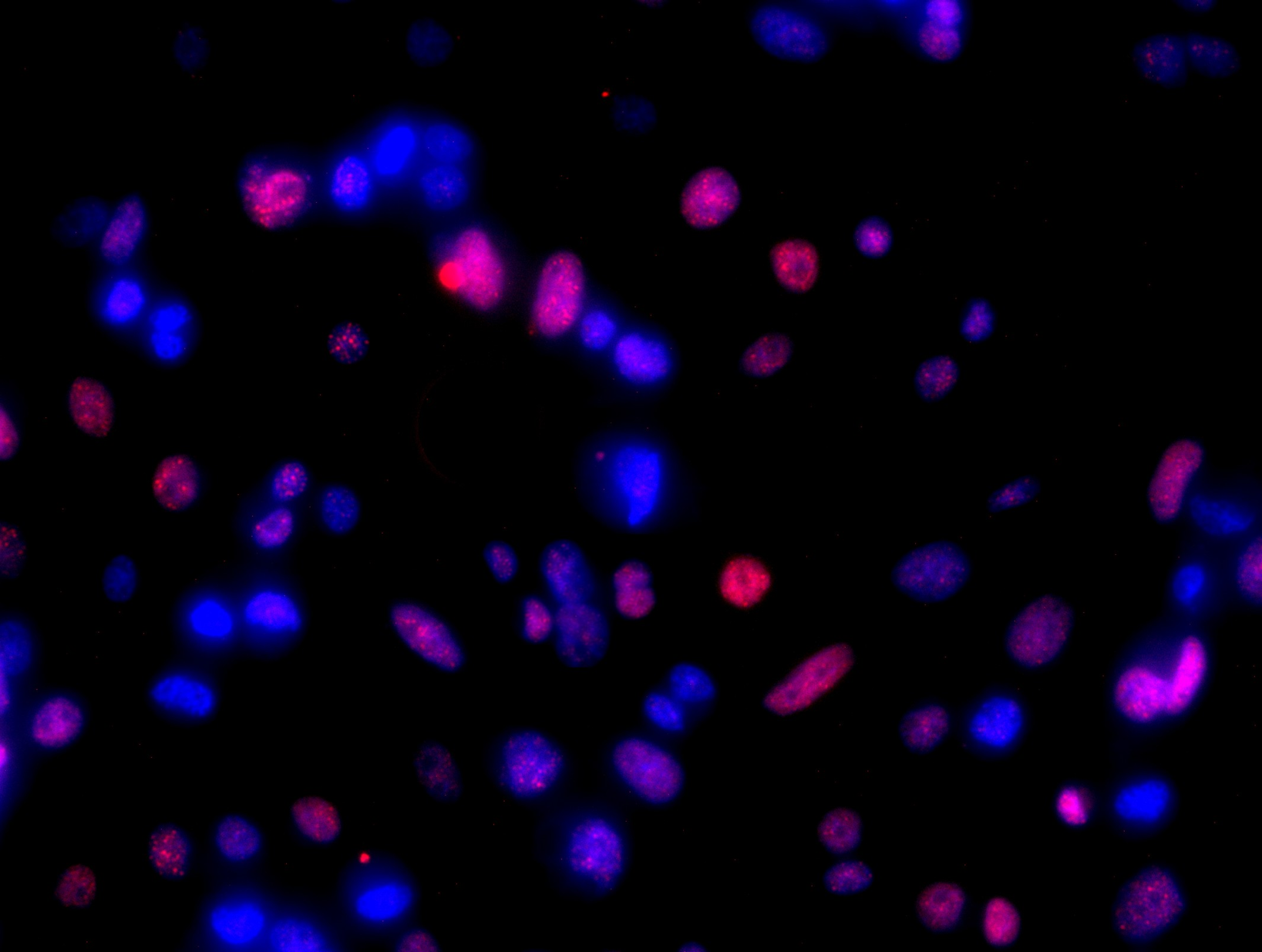

Supplement: Supplementary file 1 [file DataSheet3.ZIP › Figure 4a Microscopy images for LNCap and PC-3 cell DNA double strand breaks/Figure 4a Microscopy images for LNCap and PC-3 cell DNA double strand breaks LNCap 1h HT.tif]

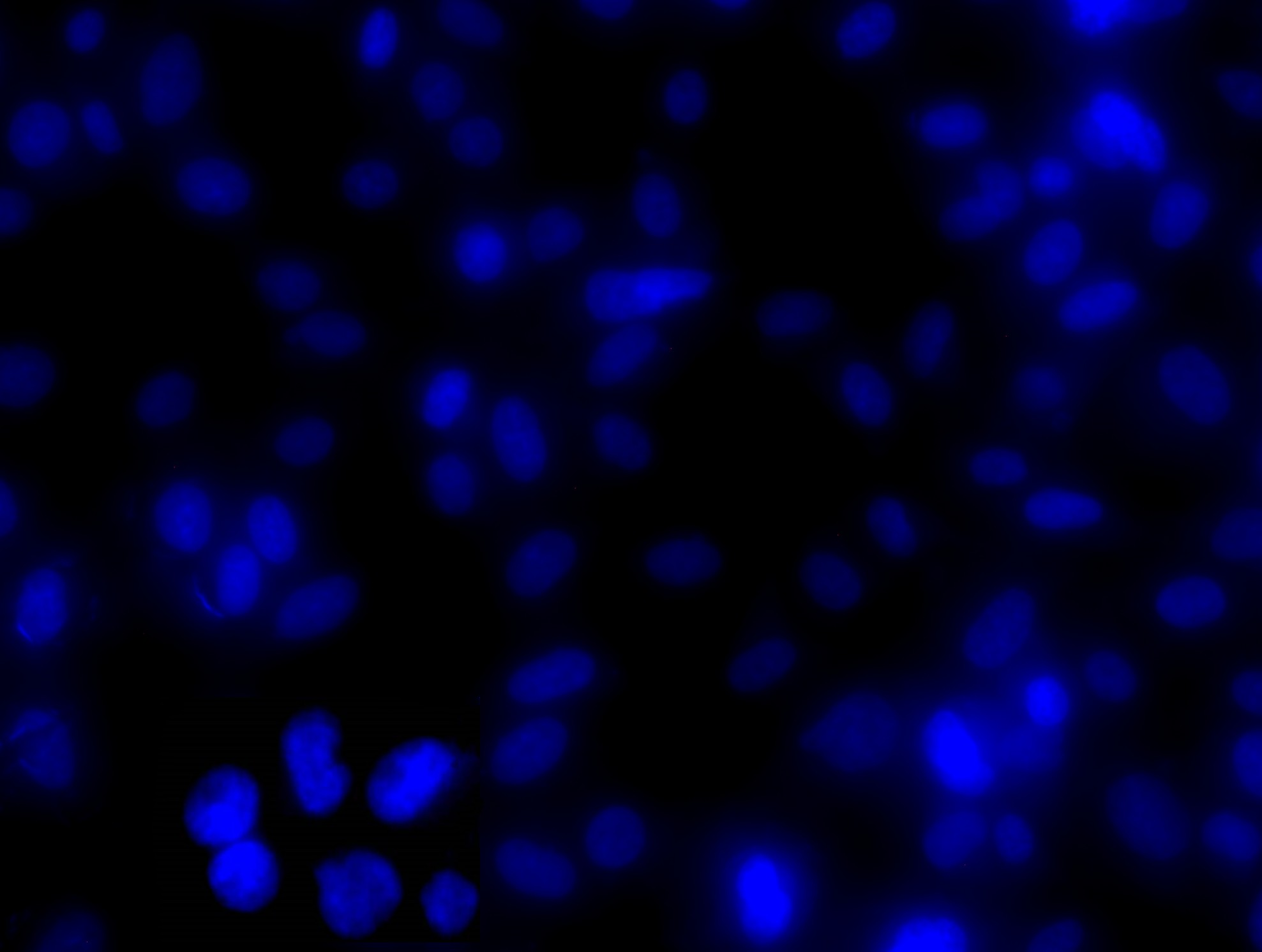

Supplement: Supplementary file 1 [file DataSheet3.ZIP › Figure 4a Microscopy images for LNCap and PC-3 cell DNA double strand breaks/Figure 4a Microscopy images for LNCap and PC-3 cell DNA double strand breaks LNCap 24h Control.tif]

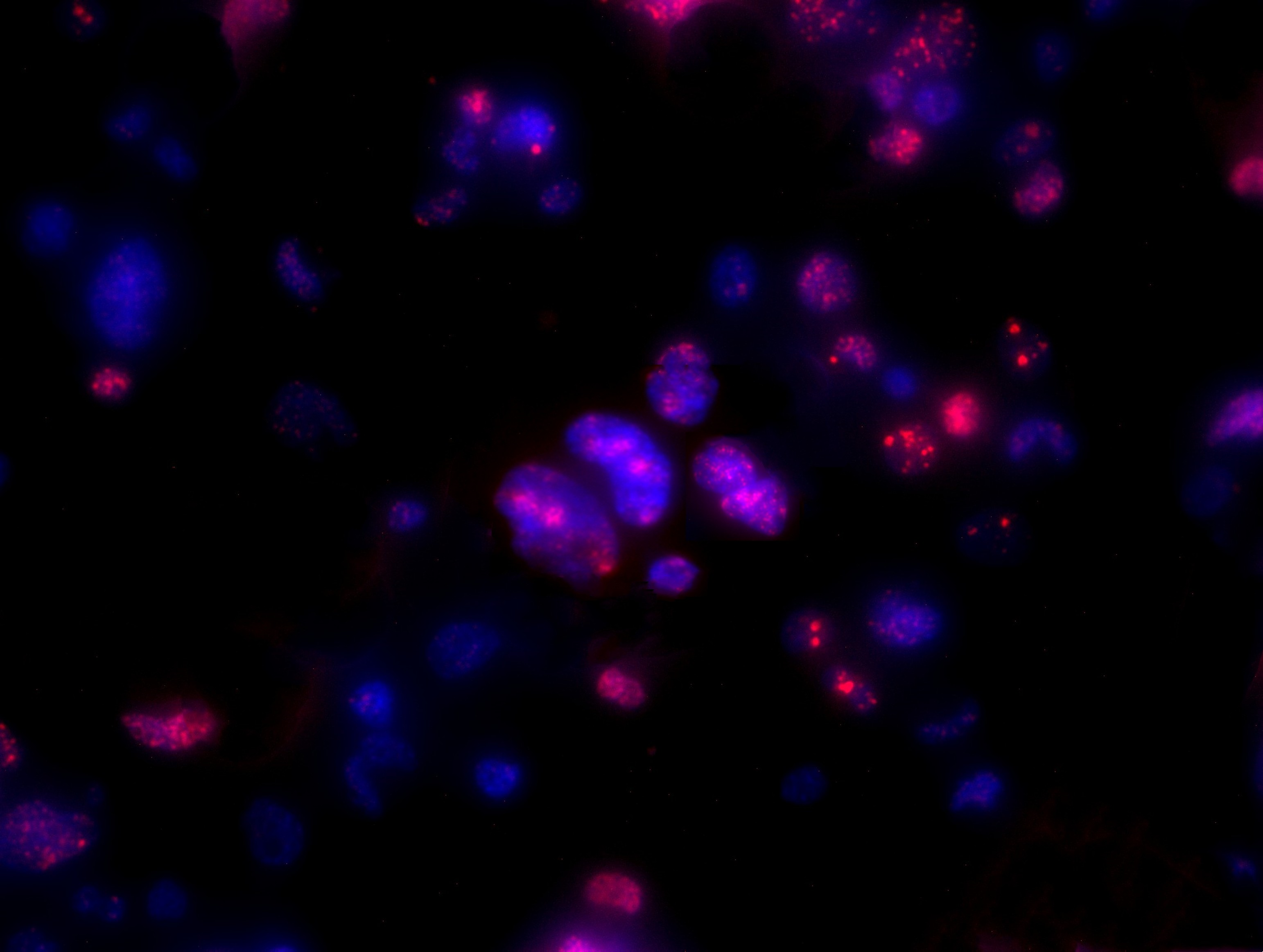

Supplement: Supplementary file 1 [file DataSheet3.ZIP › Figure 4a Microscopy images for LNCap and PC-3 cell DNA double strand breaks/Figure 4a Microscopy images for LNCap and PC-3 cell DNA double strand breaks LNCap 24h FUS-Cav + HT.tif]

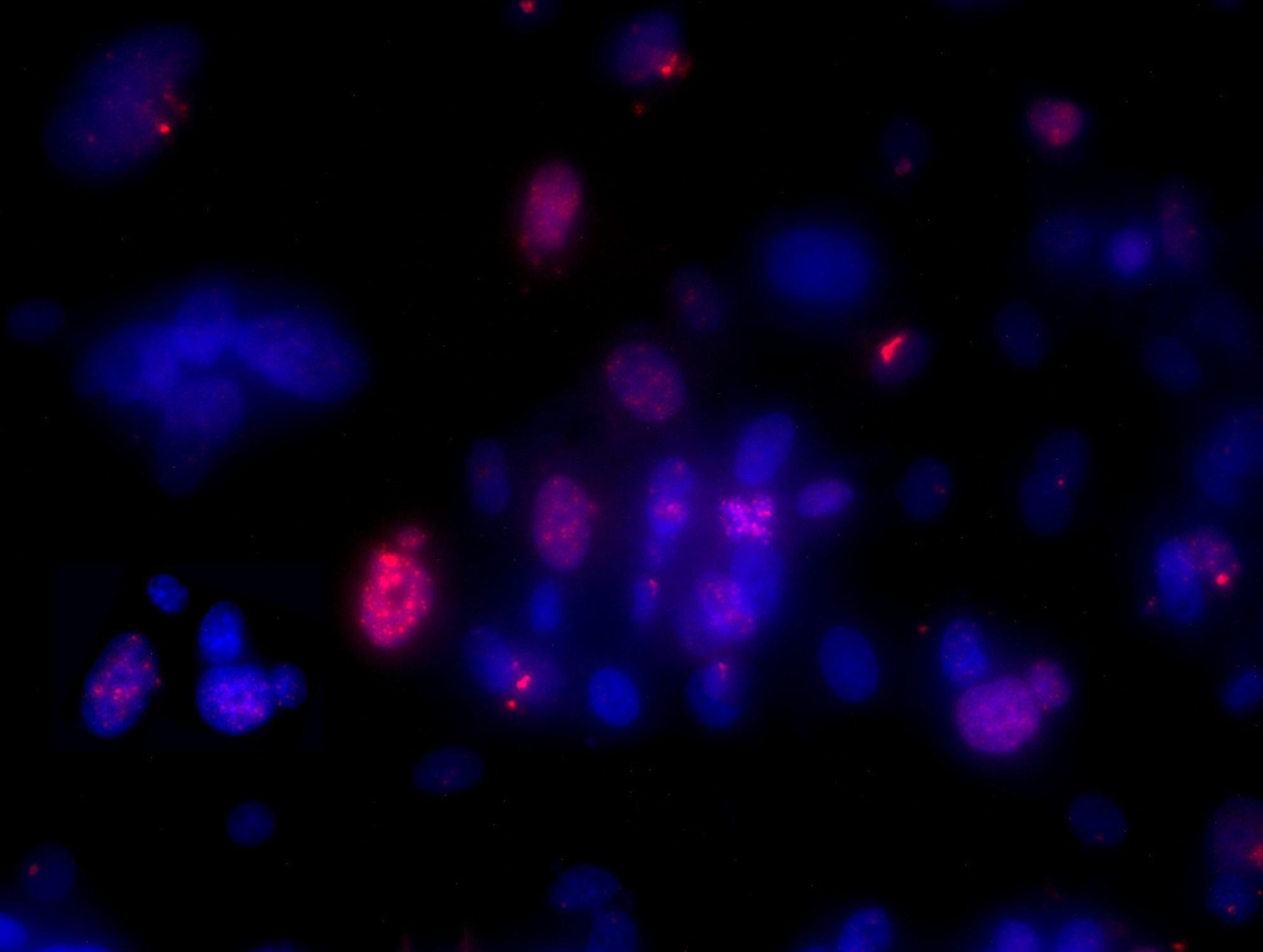

Supplement: Supplementary file 1 [file DataSheet3.ZIP › Figure 4a Microscopy images for LNCap and PC-3 cell DNA double strand breaks/Figure 4a Microscopy images for LNCap and PC-3 cell DNA double strand breaks LNCap 24h FUS-Cav.tif]

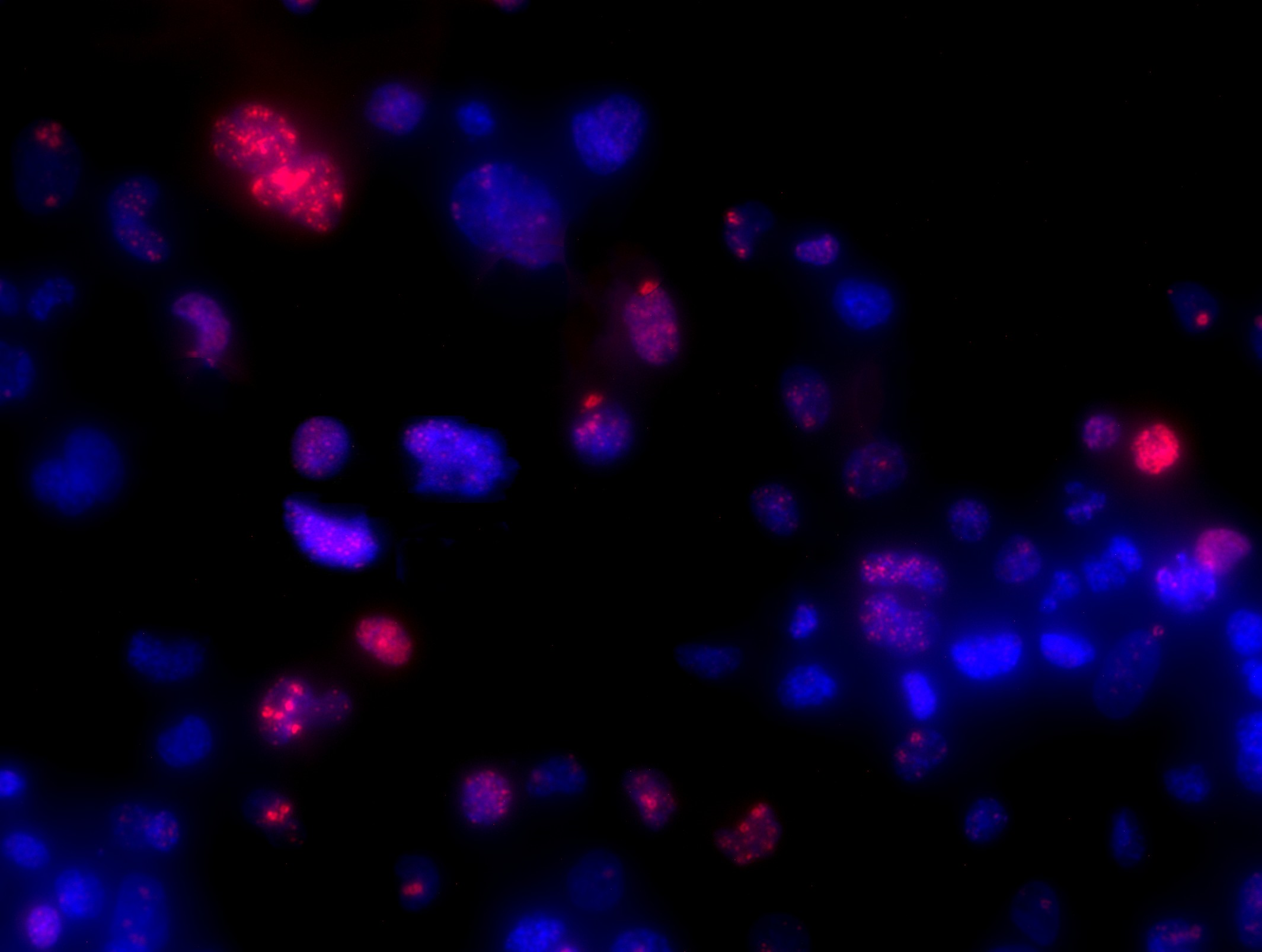

Supplement: Supplementary file 1 [file DataSheet3.ZIP › Figure 4a Microscopy images for LNCap and PC-3 cell DNA double strand breaks/Figure 4a Microscopy images for LNCap and PC-3 cell DNA double strand breaks LNCap 24h HT.tif]

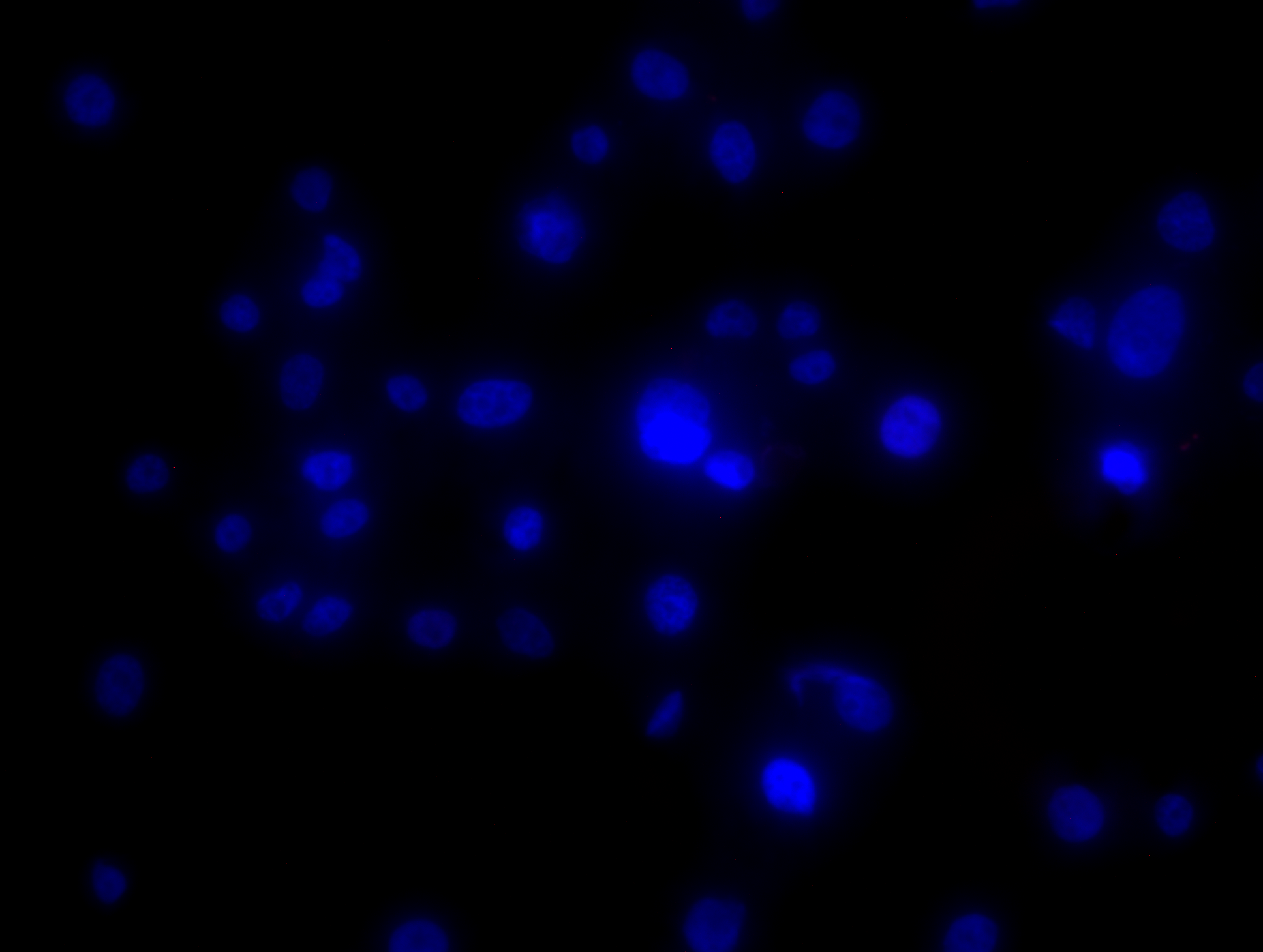

Supplement: Supplementary file 1 [file DataSheet3.ZIP › Figure 4a Microscopy images for LNCap and PC-3 cell DNA double strand breaks/Figure 4a Microscopy images for LNCap and PC-3 cell DNA double strand breaks PC-3 1h Control.tif]

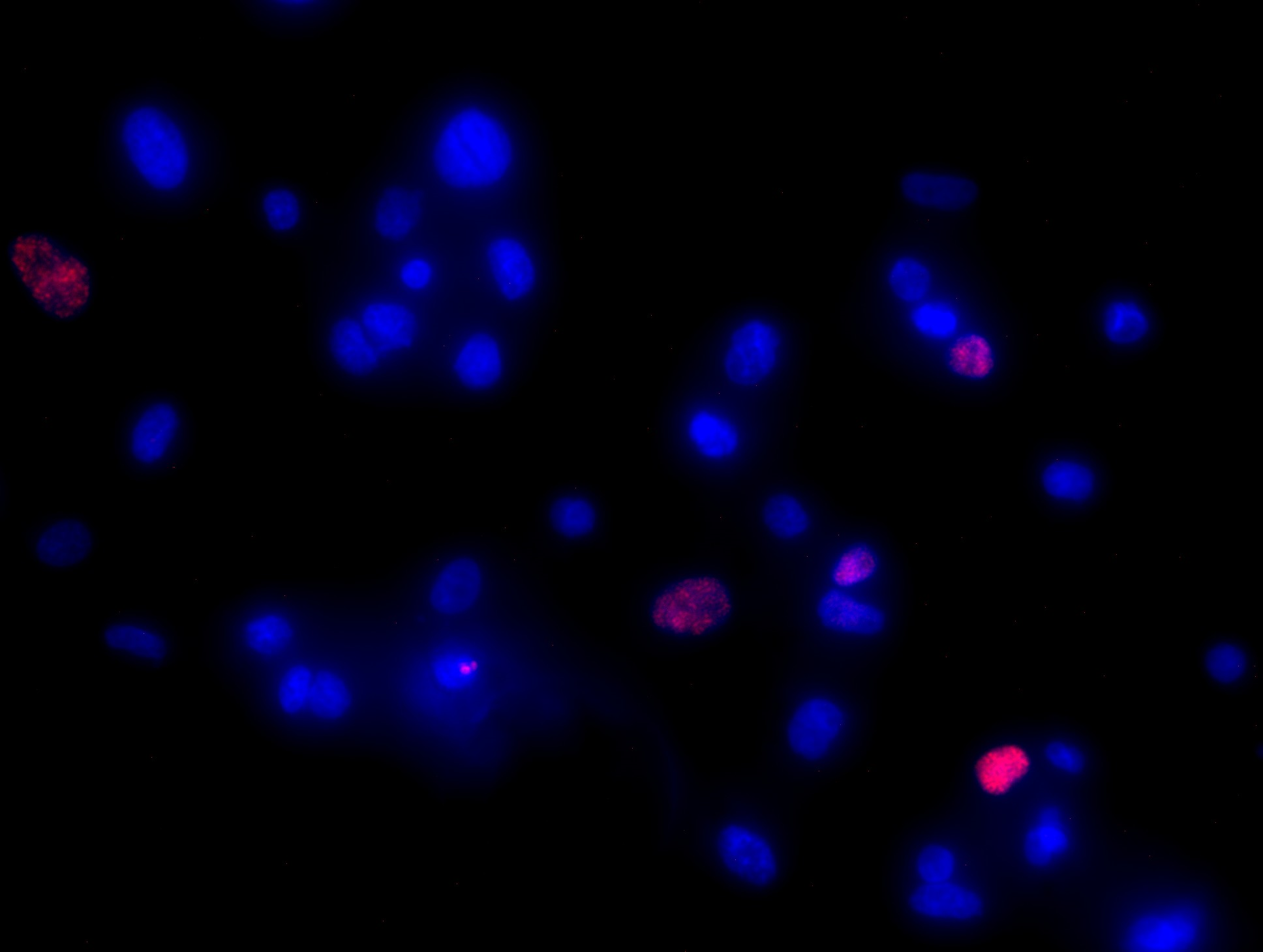

Supplement: Supplementary file 1 [file DataSheet3.ZIP › Figure 4a Microscopy images for LNCap and PC-3 cell DNA double strand breaks/Figure 4a Microscopy images for LNCap and PC-3 cell DNA double strand breaks PC-3 1h FUS-Cav.tif]

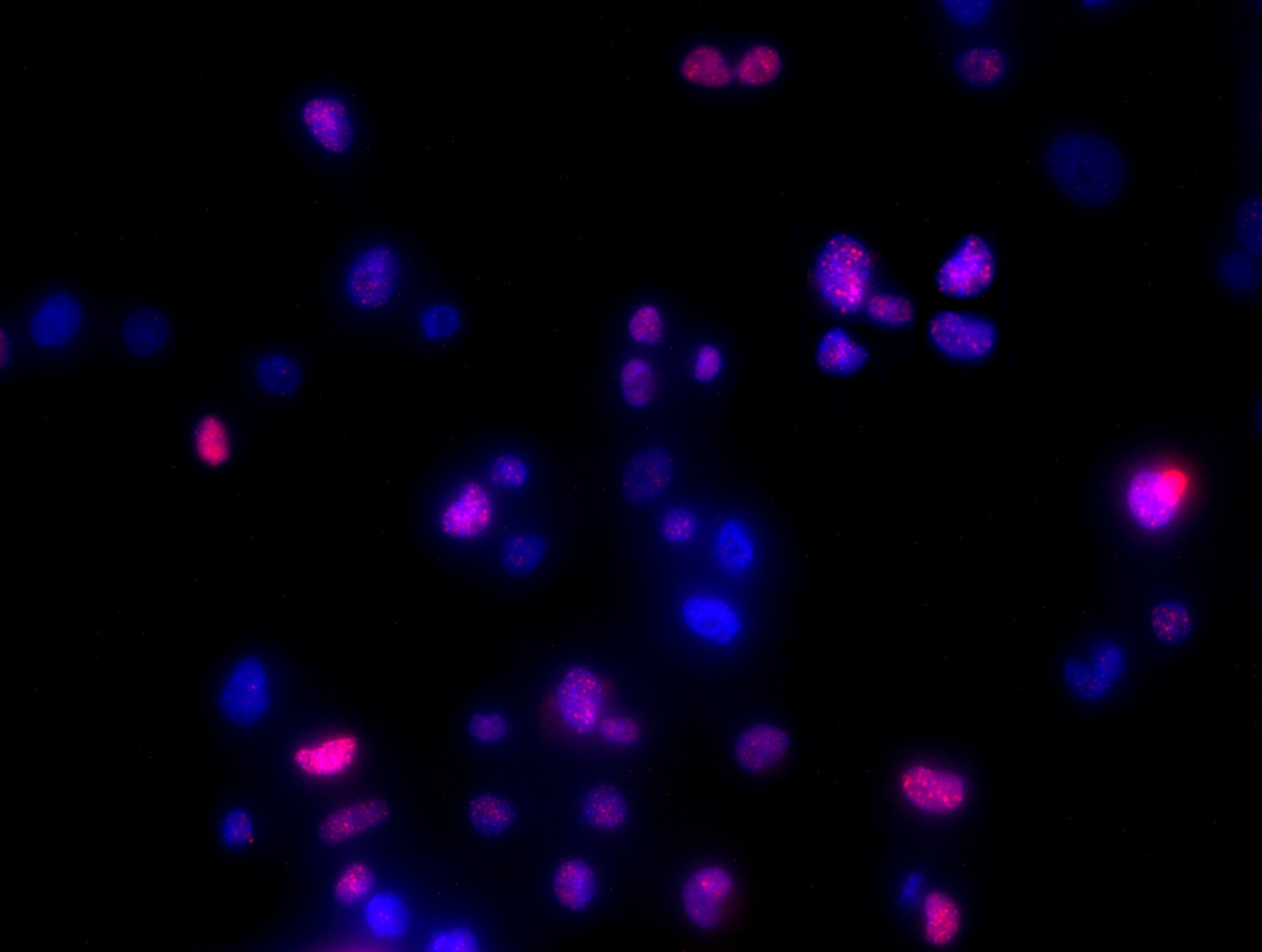

Supplement: Supplementary file 1 [file DataSheet3.ZIP › Figure 4a Microscopy images for LNCap and PC-3 cell DNA double strand breaks/Figure 4a Microscopy images for LNCap and PC-3 cell DNA double strand breaks PC-3 1h FUS-Cav+HT.tif]

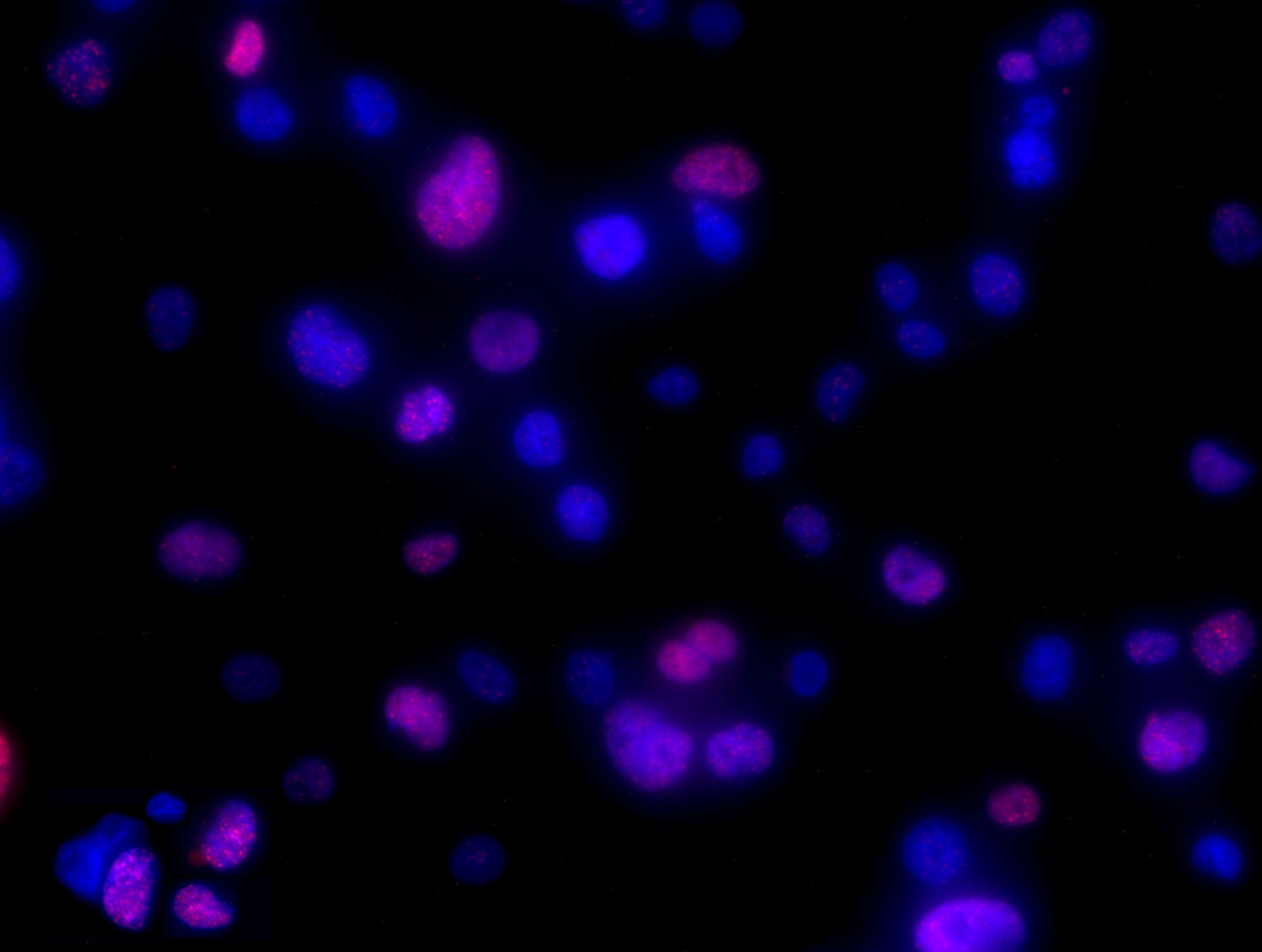

Supplement: Supplementary file 1 [file DataSheet3.ZIP › Figure 4a Microscopy images for LNCap and PC-3 cell DNA double strand breaks/Figure 4a Microscopy images for LNCap and PC-3 cell DNA double strand breaks PC-3 1h HT.tif]

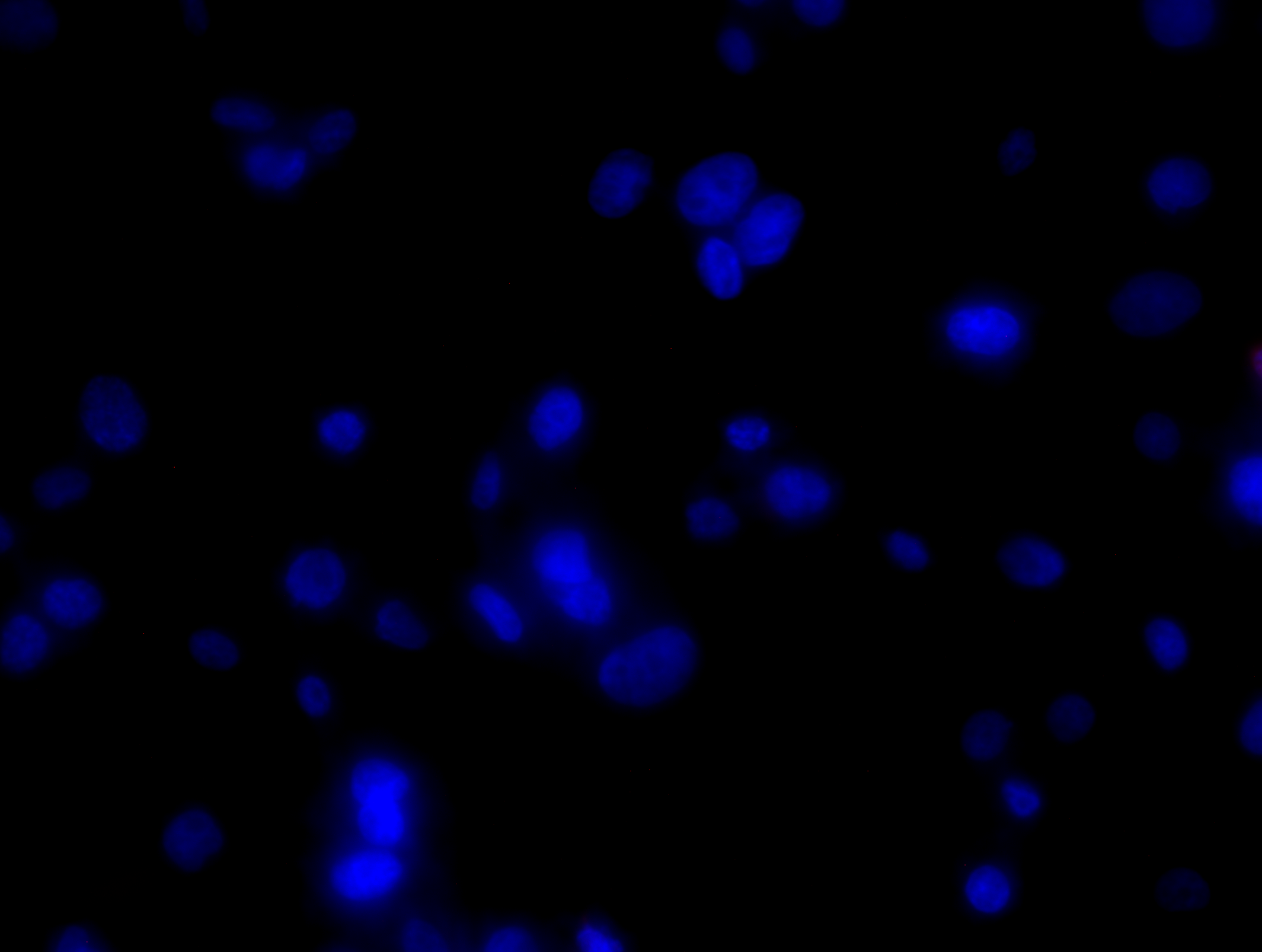

Supplement: Supplementary file 1 [file DataSheet3.ZIP › Figure 4a Microscopy images for LNCap and PC-3 cell DNA double strand breaks/Figure 4a Microscopy images for LNCap and PC-3 cell DNA double strand breaks PC-3 24h Control.tif]

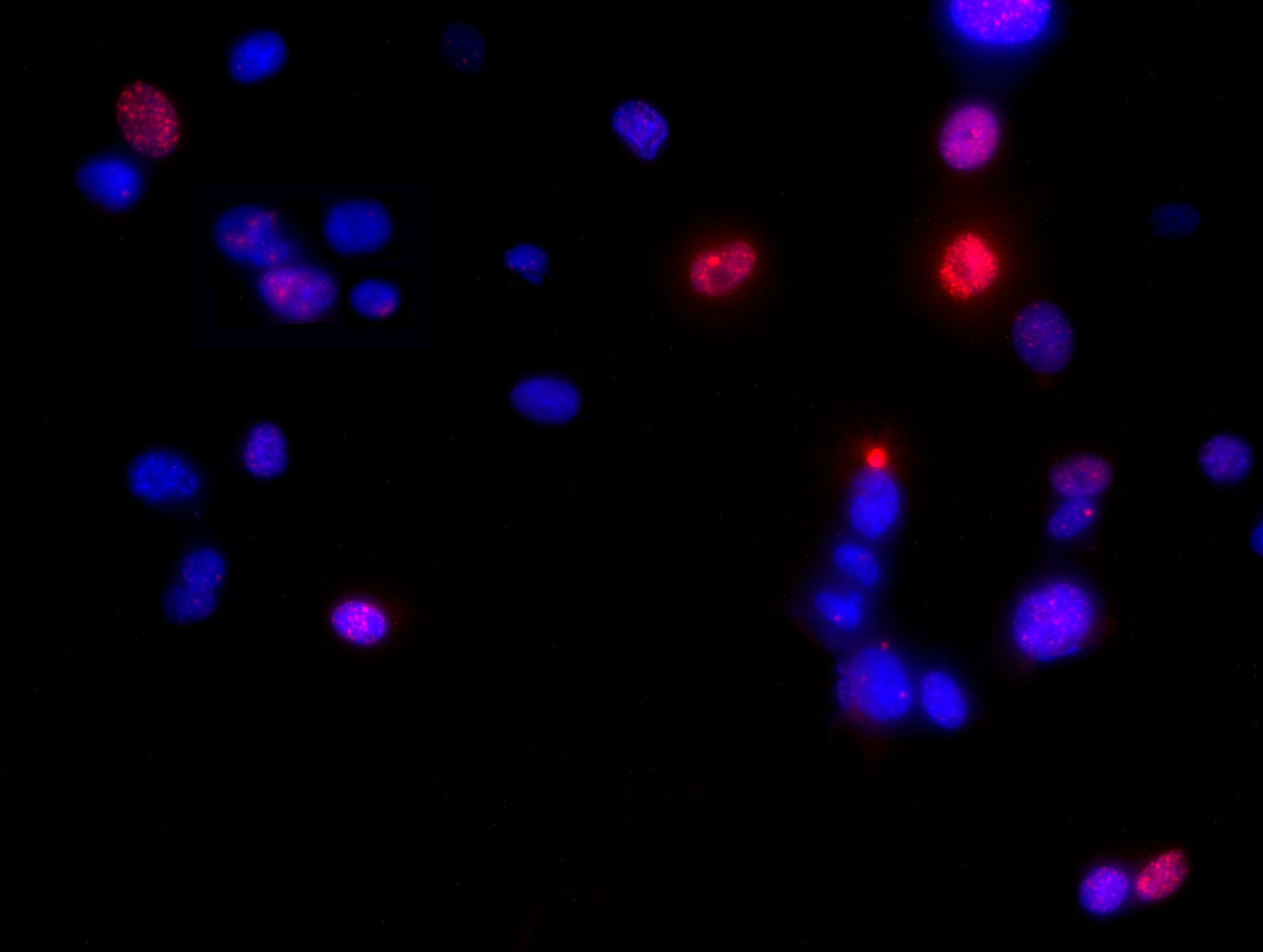

Supplement: Supplementary file 1 [file DataSheet3.ZIP › Figure 4a Microscopy images for LNCap and PC-3 cell DNA double strand breaks/Figure 4a Microscopy images for LNCap and PC-3 cell DNA double strand breaks PC-3 24h FUS-Cav.tif]

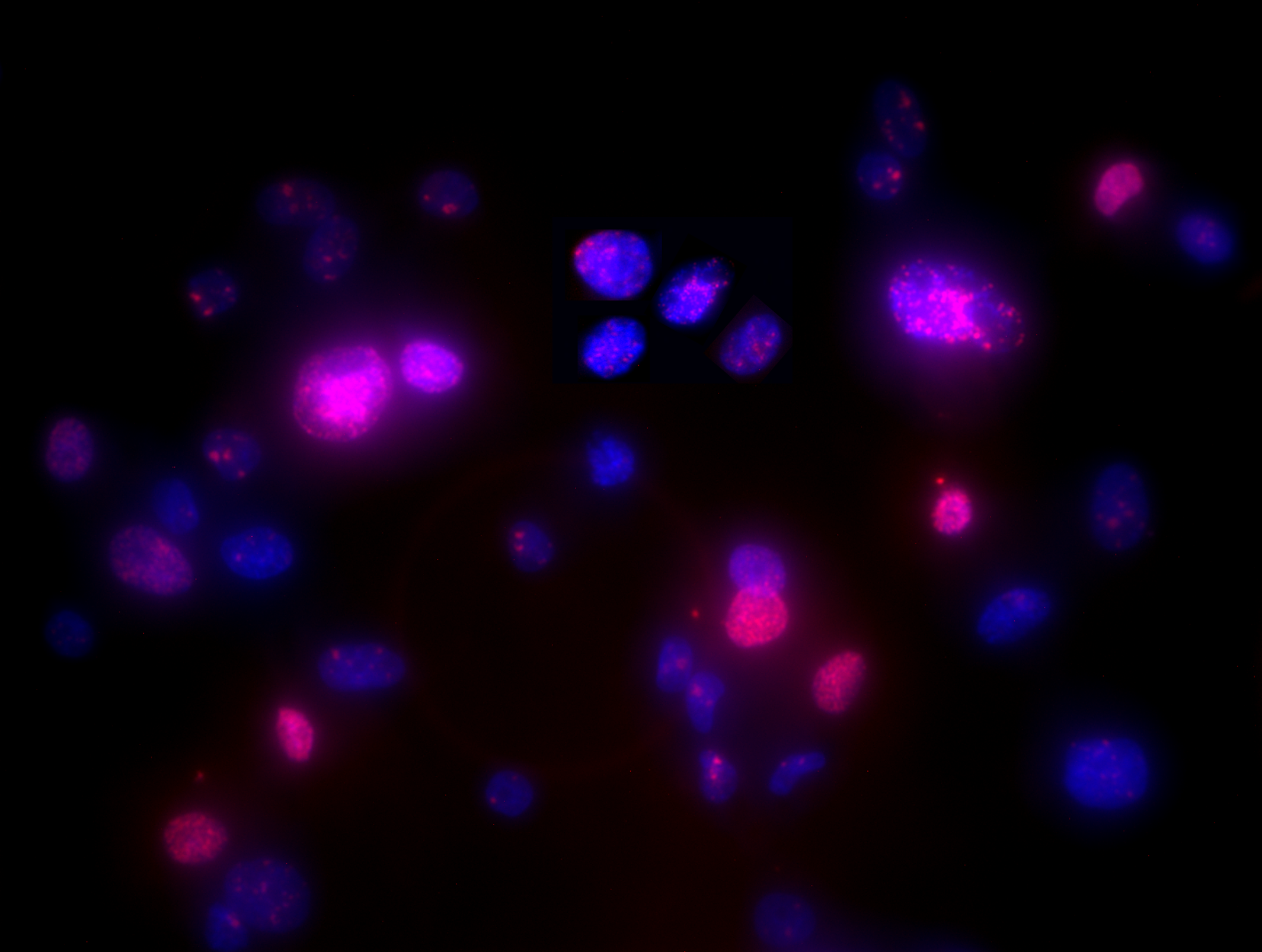

Supplement: Supplementary file 1 [file DataSheet3.ZIP › Figure 4a Microscopy images for LNCap and PC-3 cell DNA double strand breaks/Figure 4a Microscopy images for LNCap and PC-3 cell DNA double strand breaks PC-3 24h FUS-Cav+HT.tif]

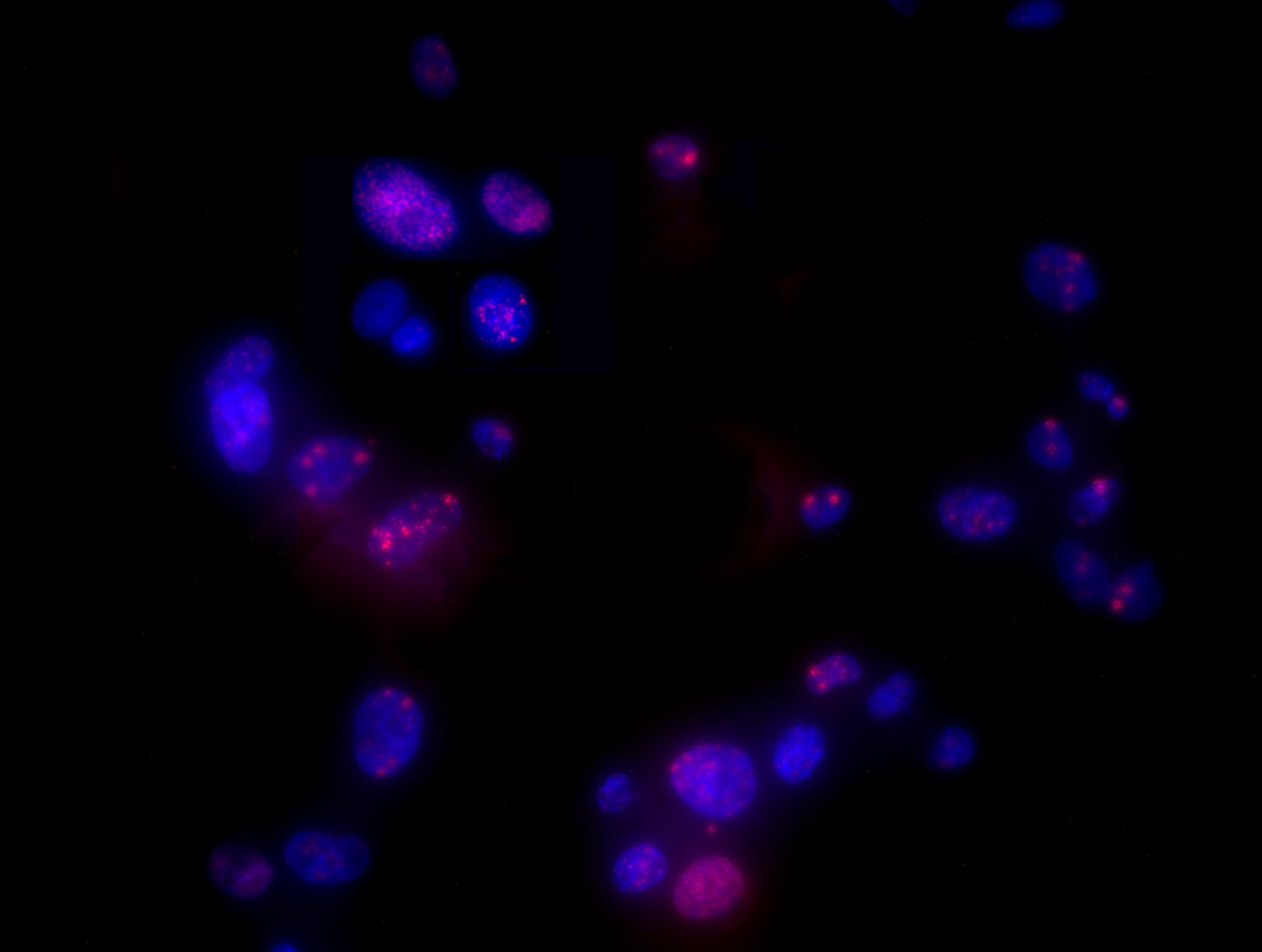

Supplement: Supplementary file 1 [file DataSheet3.ZIP › Figure 4a Microscopy images for LNCap and PC-3 cell DNA double strand breaks/Figure 4a Microscopy images for LNCap and PC-3 cell DNA double strand breaks PC-3 24h HT.tif]

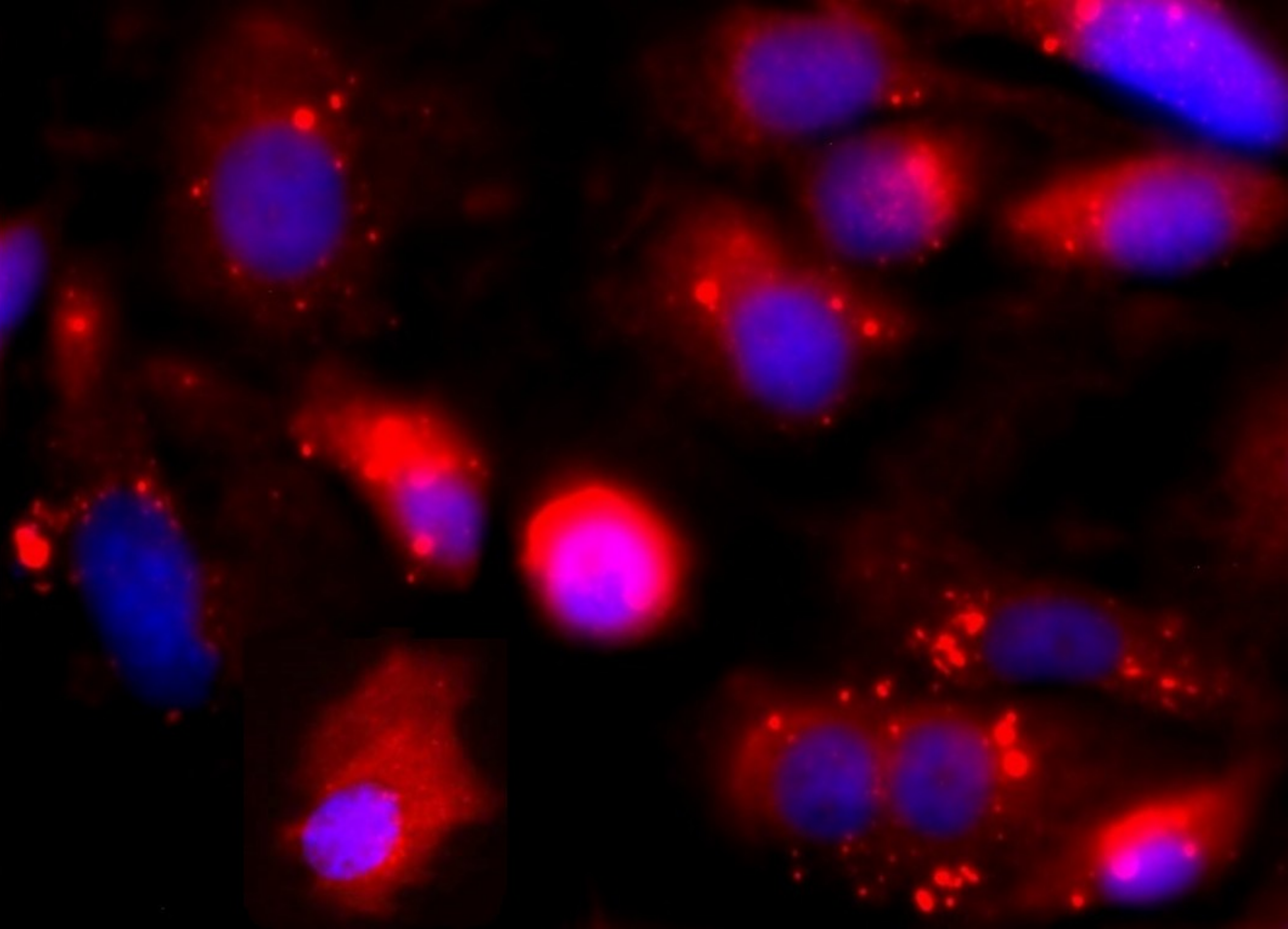

Supplement: Supplementary file 2 [file DataSheet4.ZIP › LNCap Control SRD5A 1.tif]

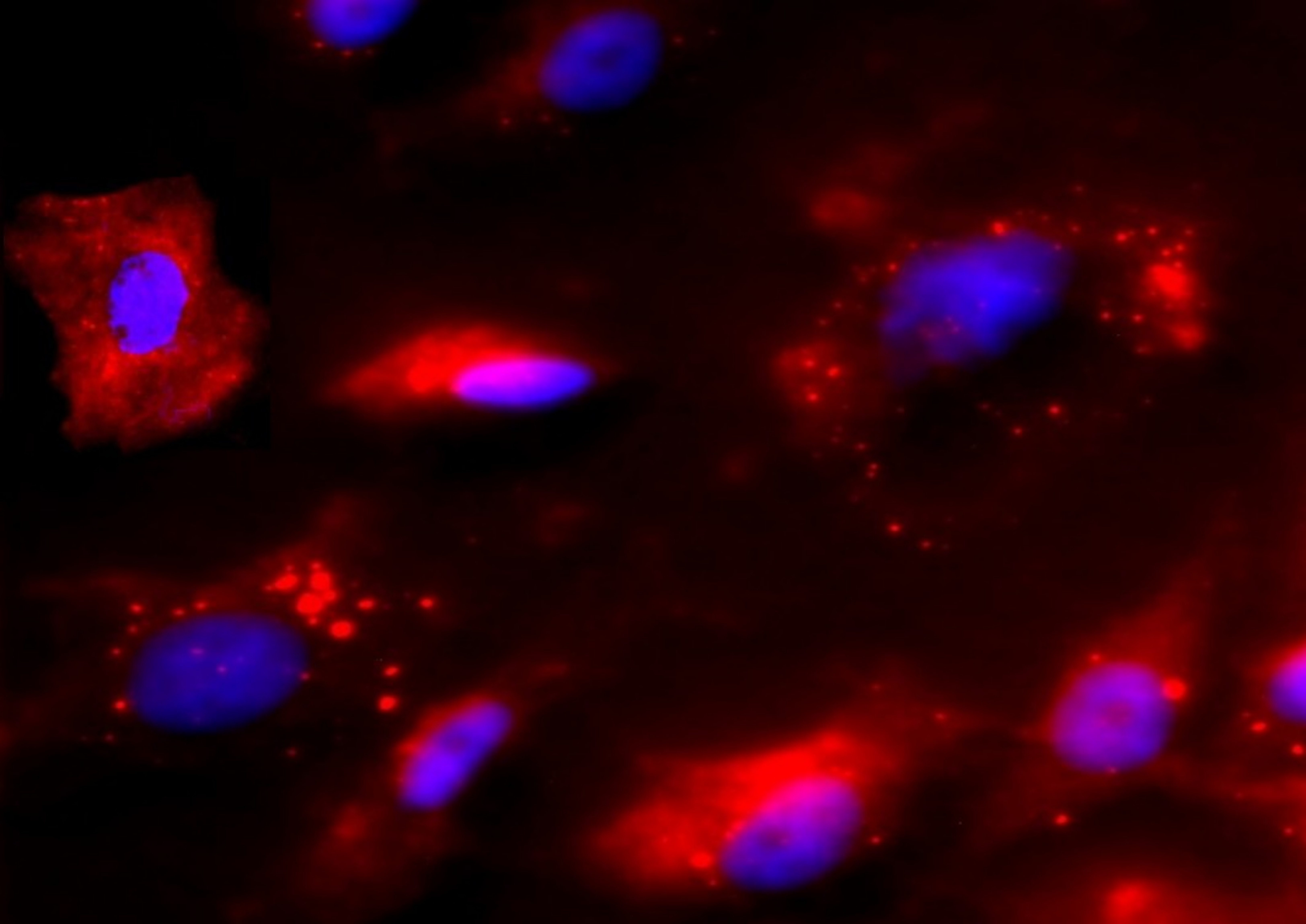

Supplement: Supplementary file 2 [file DataSheet4.ZIP › LNCap Control SRD5A 3.tif]

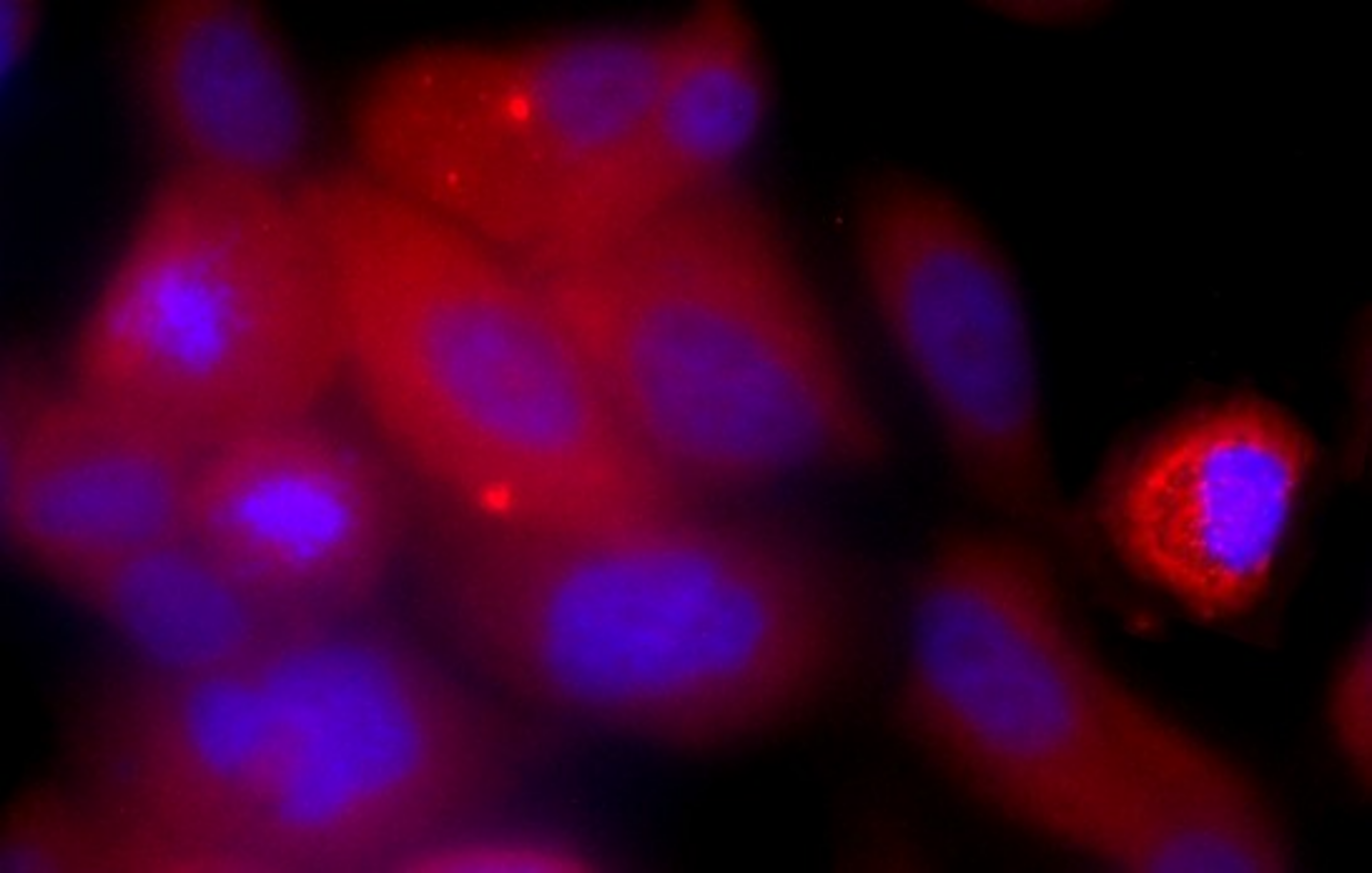

Supplement: Supplementary file 2 [file DataSheet4.ZIP › LNCap FUS-Cav SRD5A 1.tif]

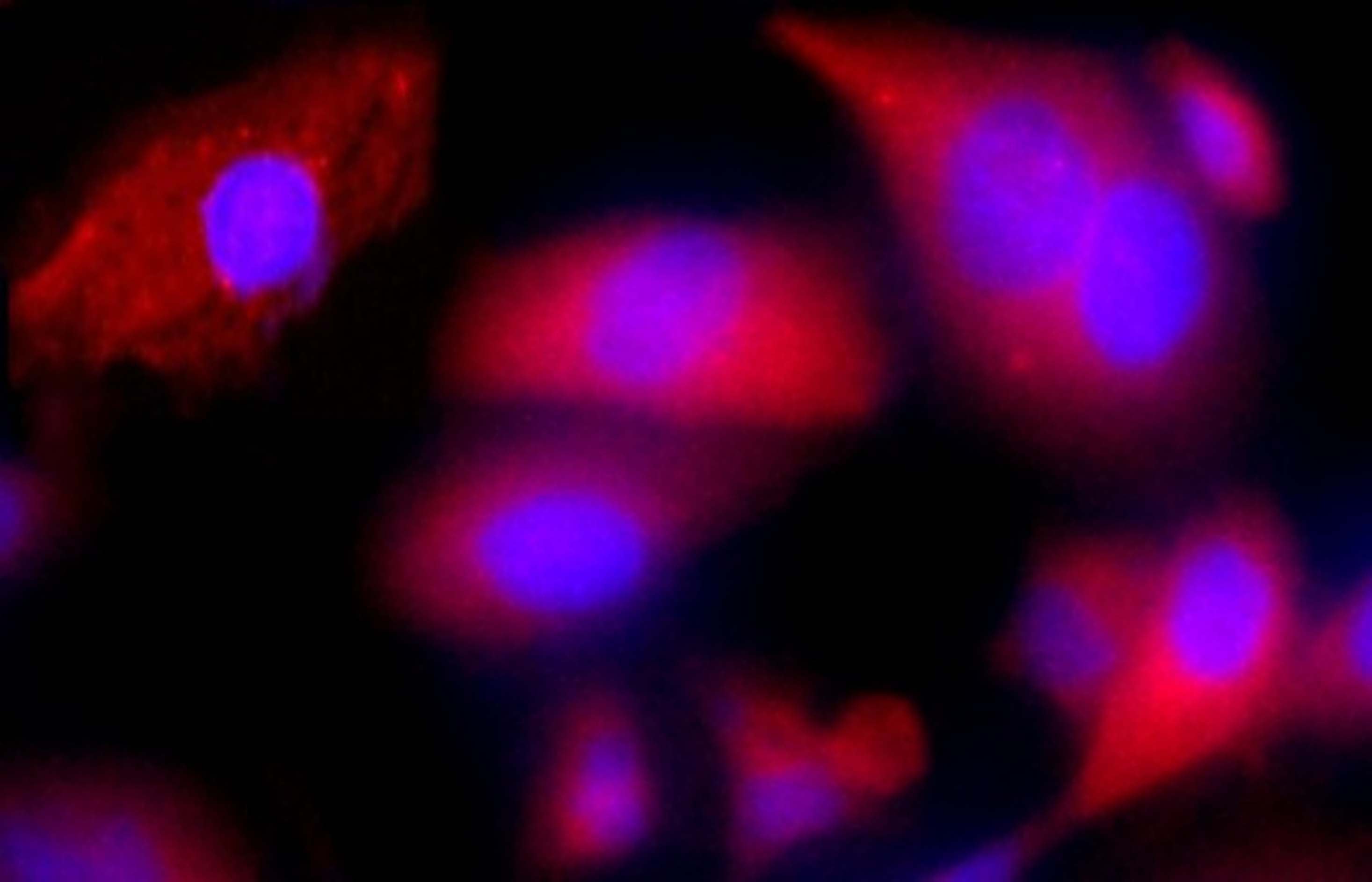

Supplement: Supplementary file 2 [file DataSheet4.ZIP › LNCap FUS-Cav SRD5A 3.tif]

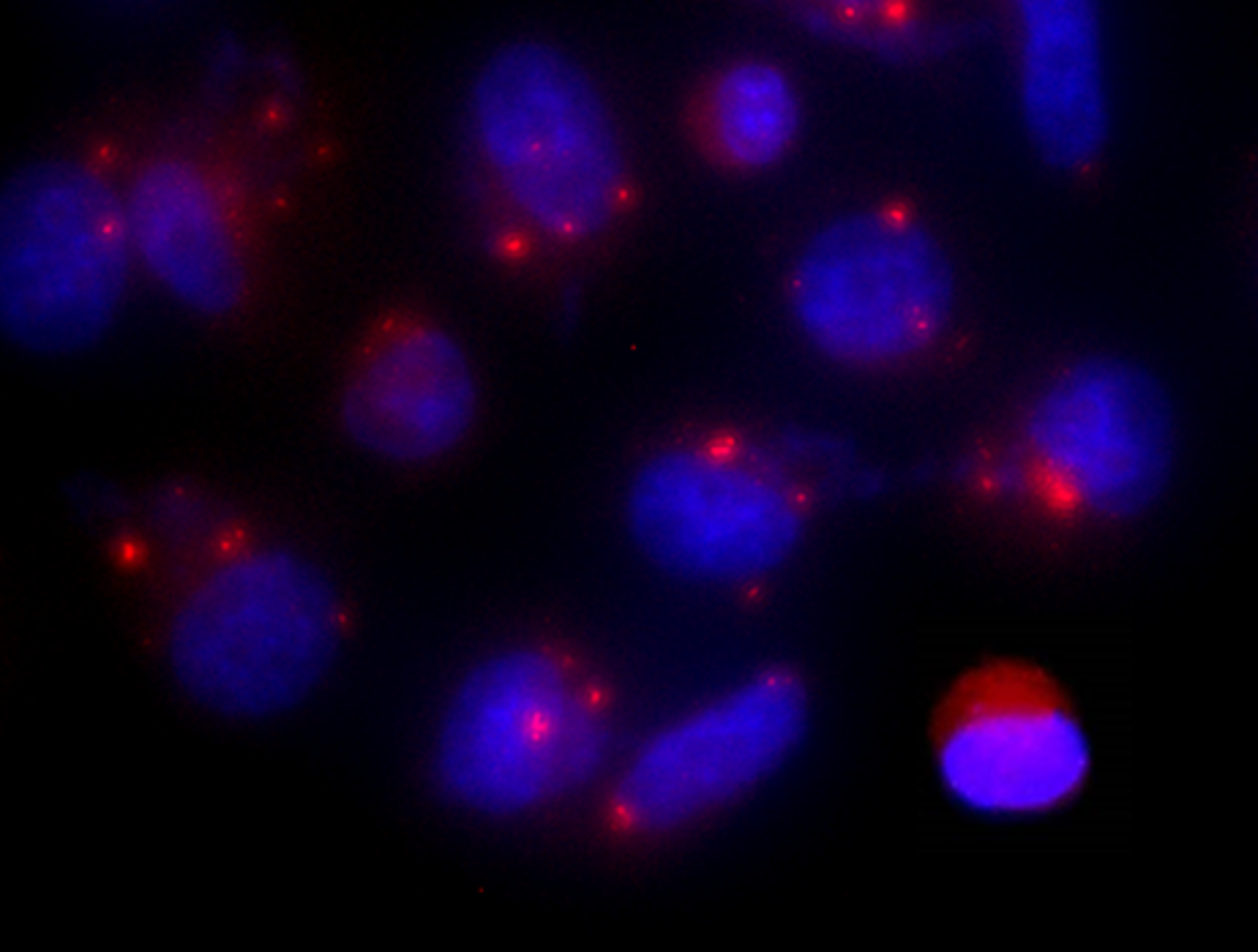

Supplement: Supplementary file 2 [file DataSheet4.ZIP › LNCap FUS-Cav+HT SRD5A 1.tif]

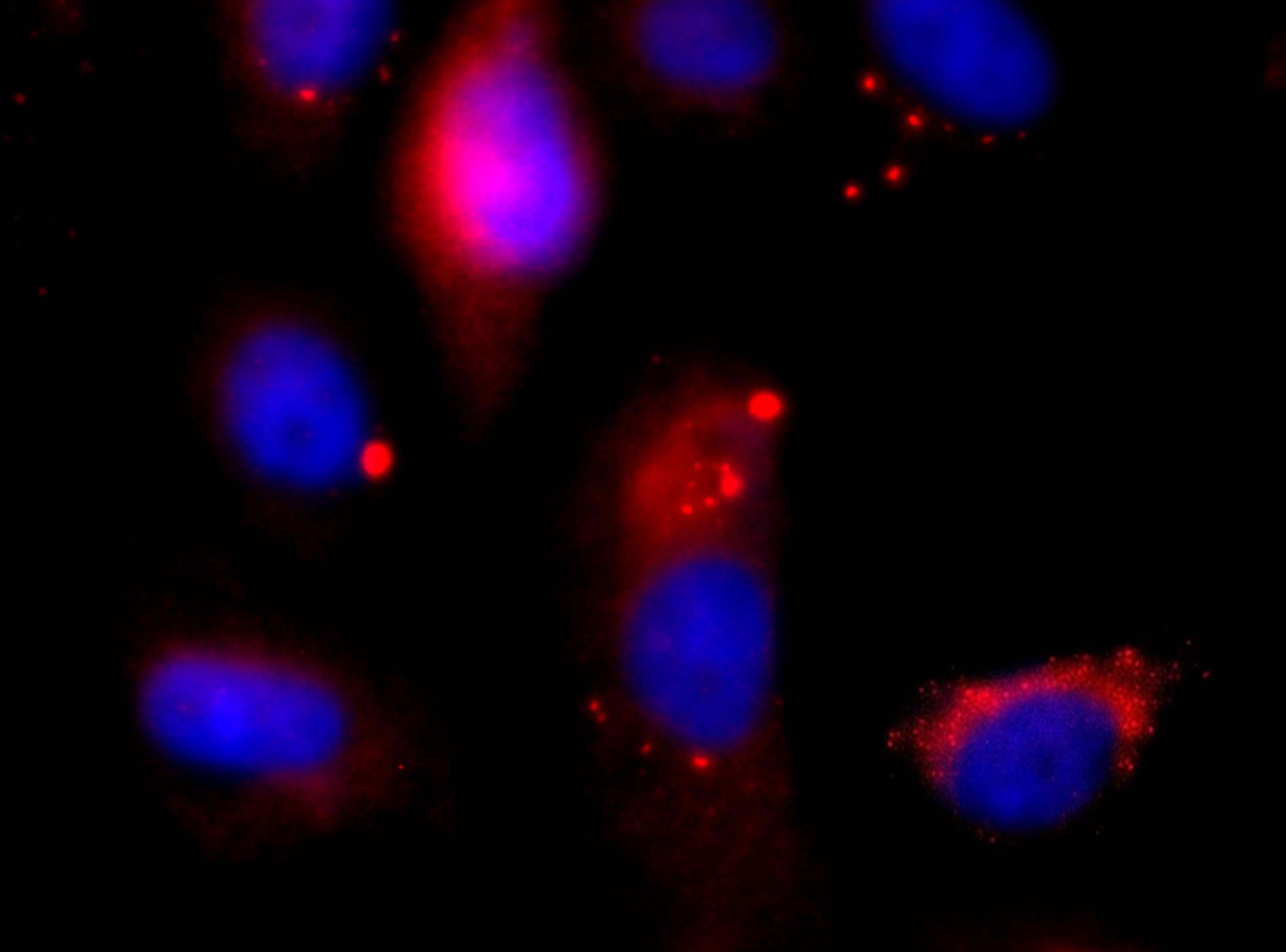

Supplement: Supplementary file 2 [file DataSheet4.ZIP › LNCap FUS-Cav+HT SRD5A 3.tif]

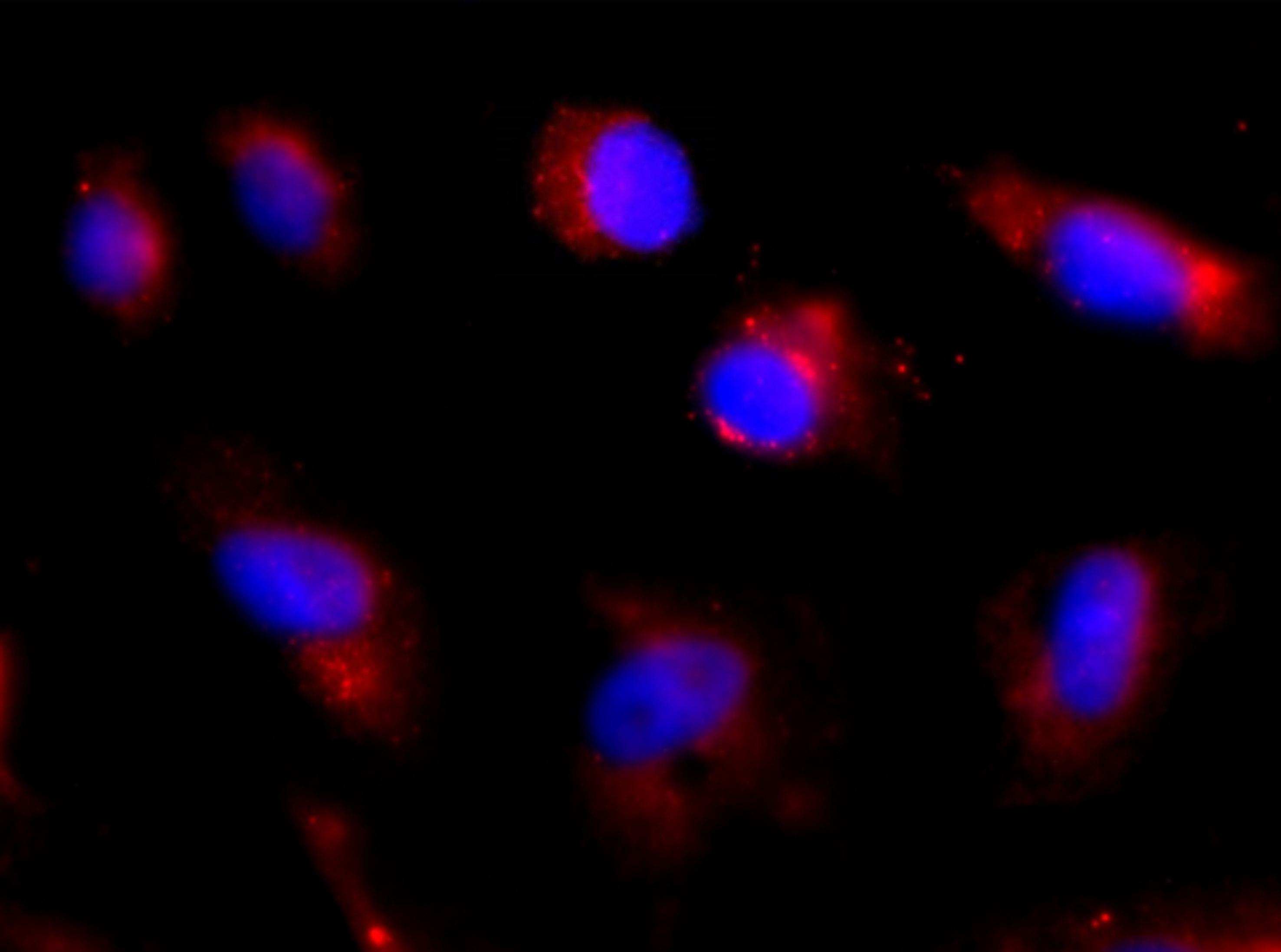

Supplement: Supplementary file 2 [file DataSheet4.ZIP › LNCap HT SRD5A 1.tif]

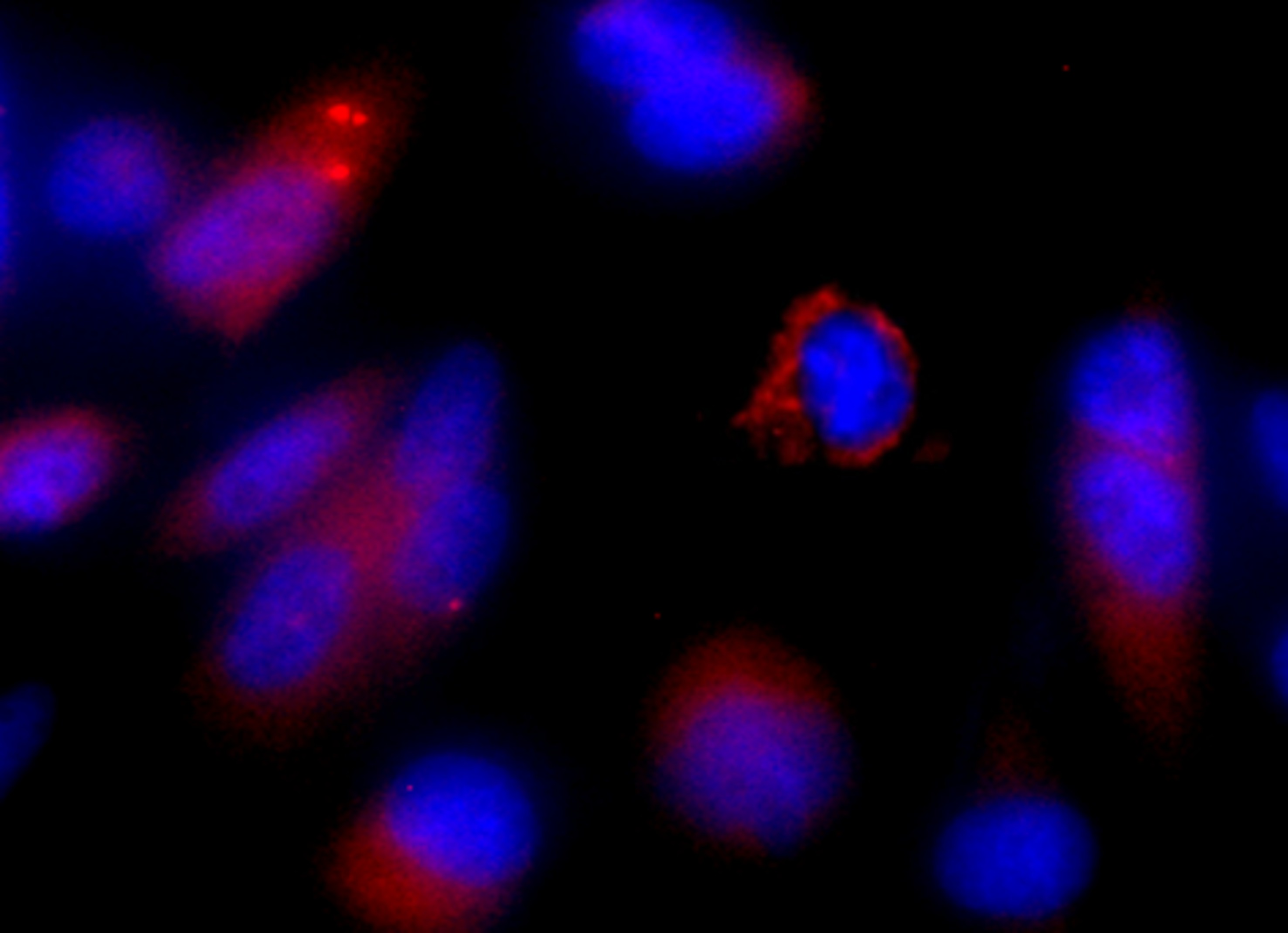

Supplement: Supplementary file 2 [file DataSheet4.ZIP › LNCap HT SRD5A 3.tif]

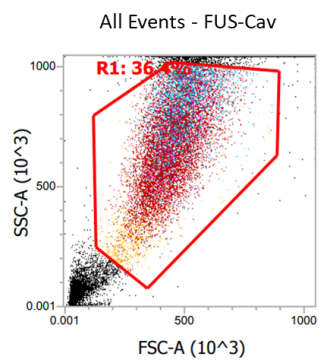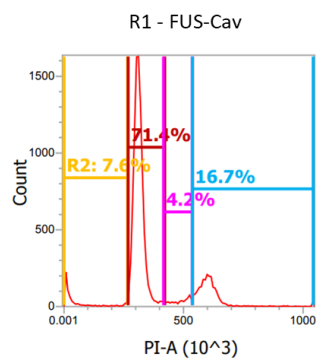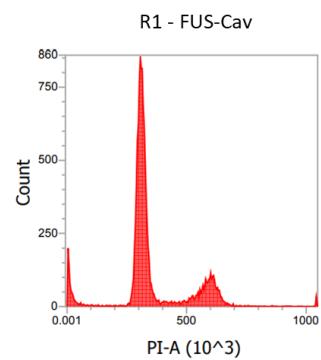

Supplement: Supplementary file 3 [file DataSheet1.ZIP › Figure 5a Representative flow cytometry results exhibit the percentage of cells in various cell cycles following each treatment/LNCap FUS-Cav/gating Cell cycle LNCap_FUS-Cav.pdf]

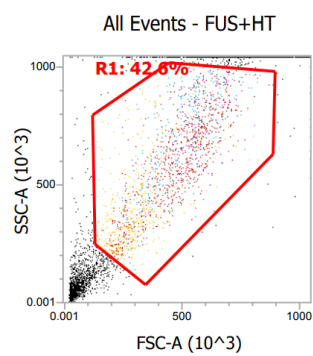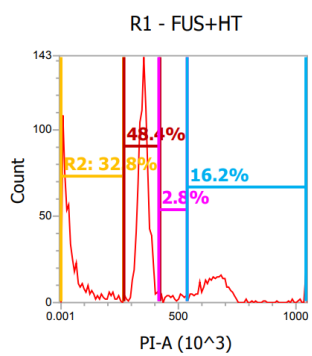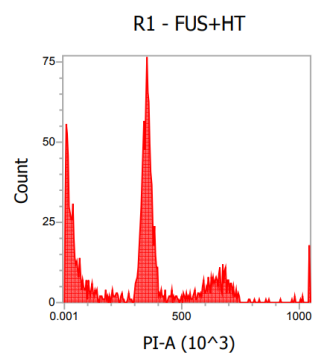

Supplement: Supplementary file 3 [file DataSheet1.ZIP › Figure 5a Representative flow cytometry results exhibit the percentage of cells in various cell cycles following each treatment/LNCap FUS-Cav+HT/gating LNCap FUS-Cav+HT.pdf]

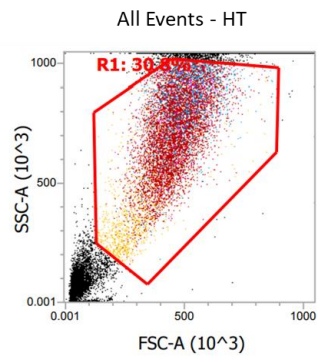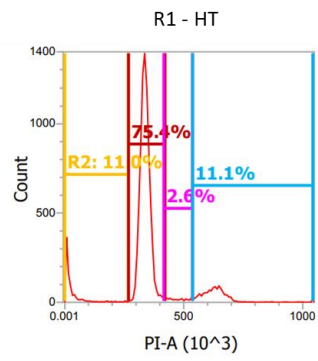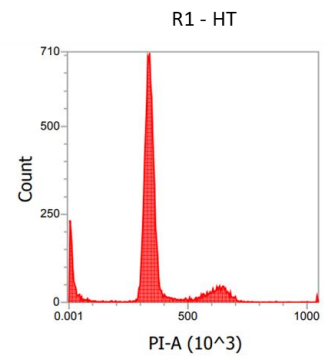

Supplement: Supplementary file 3 [file DataSheet1.ZIP › Figure 5a Representative flow cytometry results exhibit the percentage of cells in various cell cycles following each treatment/LNCap HT/gating Cell cycle LNCap_HT.pdf]

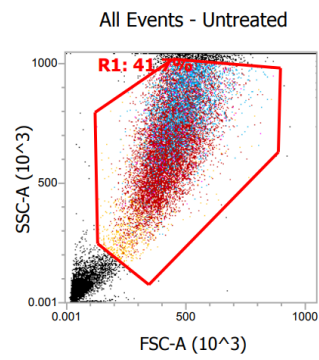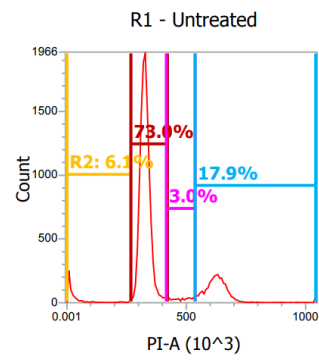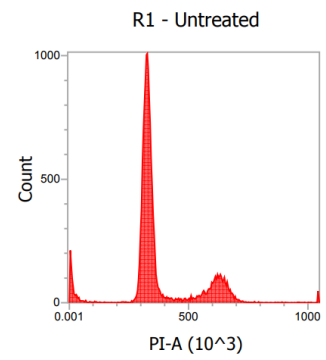

Supplement: Supplementary file 3 [file DataSheet1.ZIP › Figure 5a Representative flow cytometry results exhibit the percentage of cells in various cell cycles following each treatment/LNCap Untreated control/gating Cell cycle LNCap_Untreate.pdf]

All Events - FUS-Cav + HT

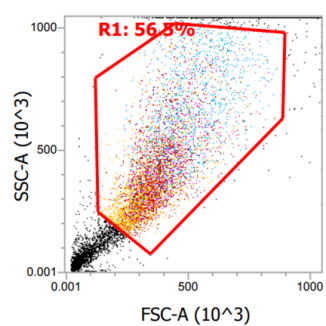

R1 - FUS-Cav + HT

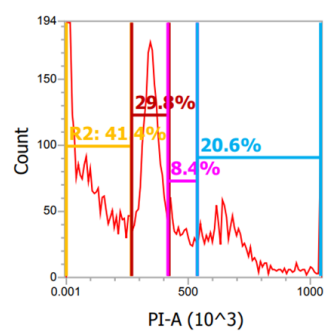

R1 - FUS-Cav + HT

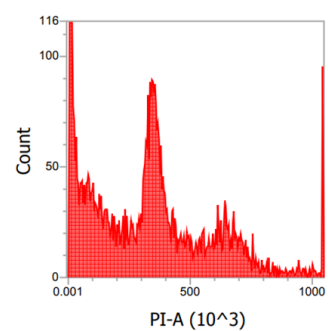

Supplement: Supplementary file 3 [file DataSheet1.ZIP › Figure 5a Representative flow cytometry results exhibit the percentage of cells in various cell cycles following each treatment/PC-3 FUS-Cav +HT/gating Cell cycle_PC-3_FUS+HT.pdf]

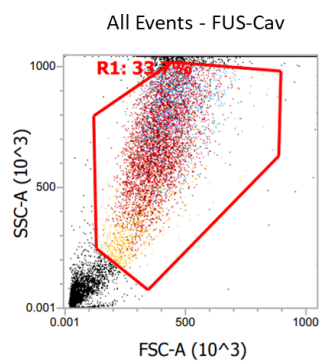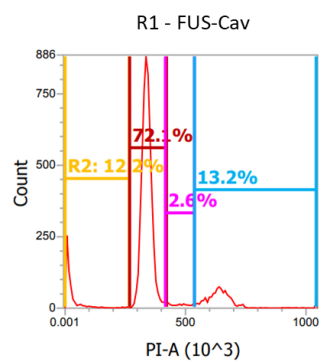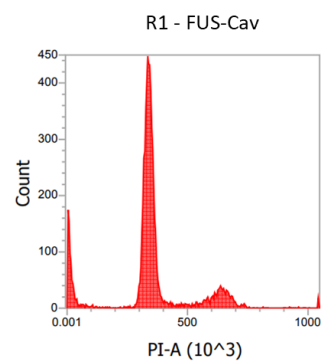

Supplement: Supplementary file 3 [file DataSheet1.ZIP › Figure 5a Representative flow cytometry results exhibit the percentage of cells in various cell cycles following each treatment/PC-3 FUS-Cav/gating Cell cycle_PC-3_FUS-Cav.pdf]

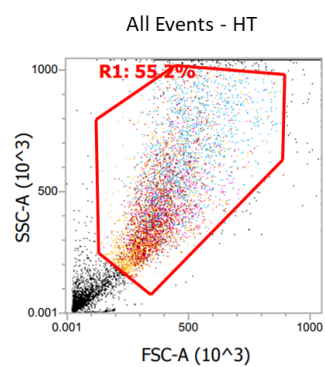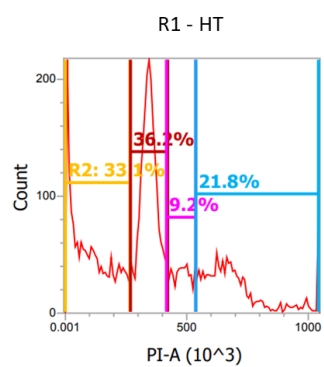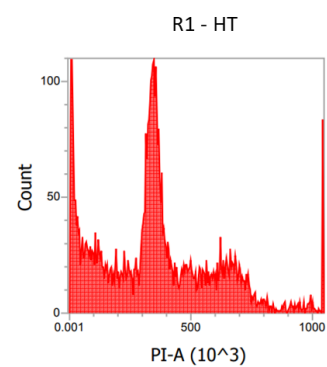

Supplement: Supplementary file 3 [file DataSheet1.ZIP › Figure 5a Representative flow cytometry results exhibit the percentage of cells in various cell cycles following each treatment/PC-3 HT/gating Cell cycle(4)_PC-3_HT.pdf]

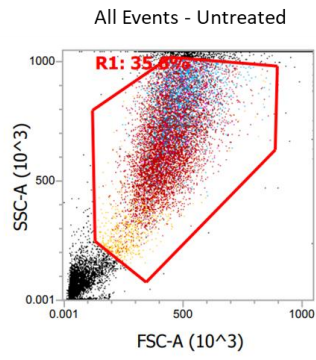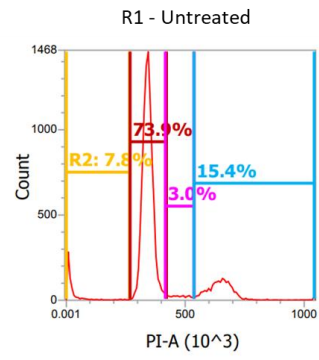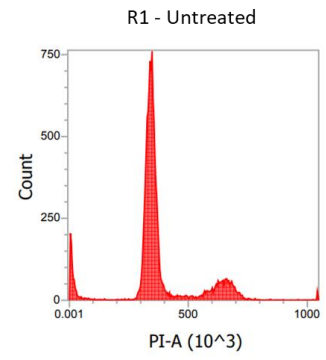

Supplement: Supplementary file 3 [file DataSheet1.ZIP › Figure 5a Representative flow cytometry results exhibit the percentage of cells in various cell cycles following each treatment/PC-3 Untreated control/gating Cell cycle_PC-3_Untreated.pdf]

## Control

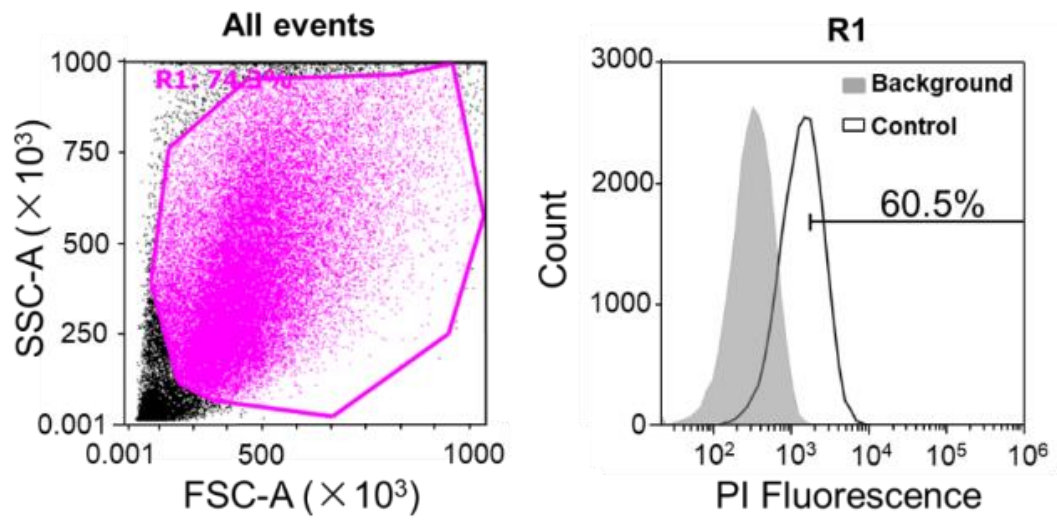

## FUS-Cav

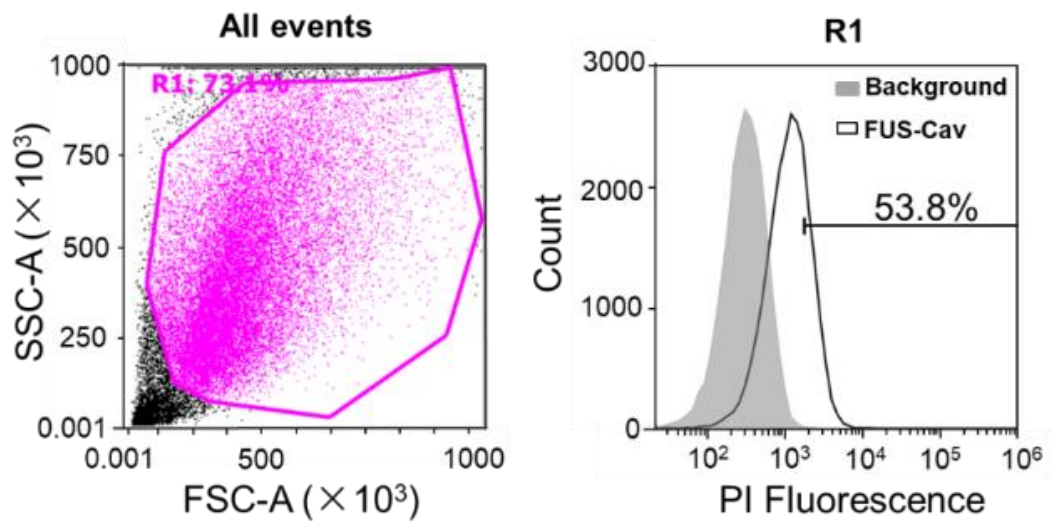

## HT

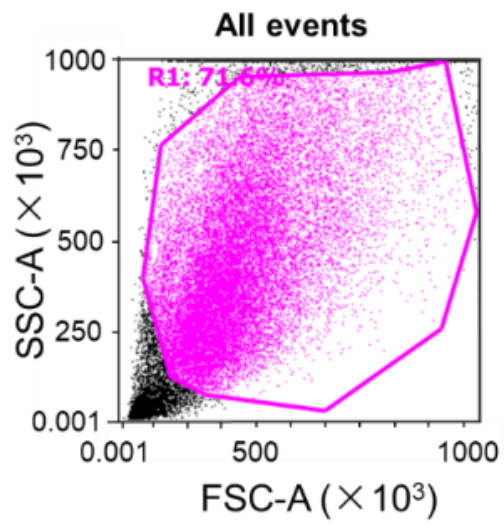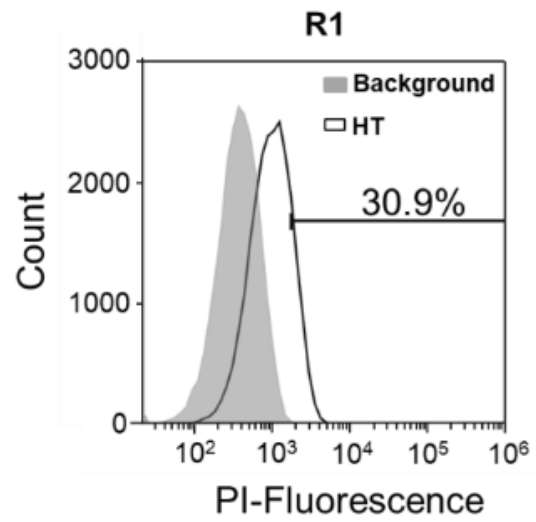

## FUS-Cav + HT

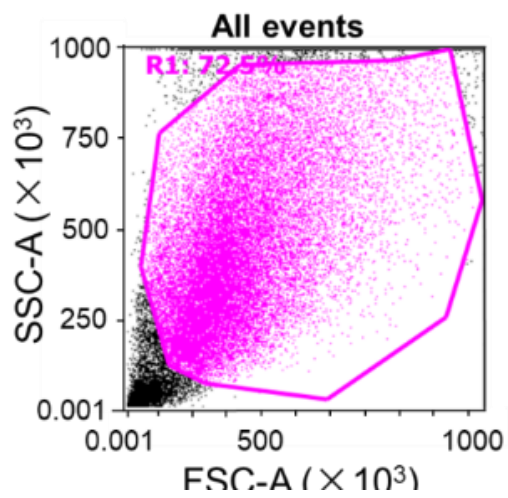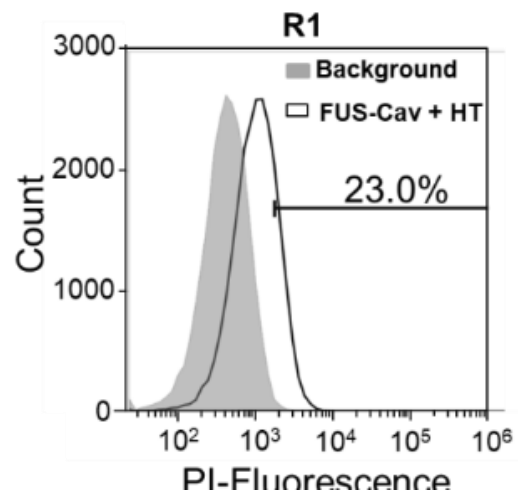

Supplement: Supplementary file 3 [file DataSheet1.ZIP › Figure 6b Flow cytometry results exhibit the percentage of immunofluorescence-positive cells/LNCap SRD5A 1/gating LNCap SRD5A 1.pdf]

## Control

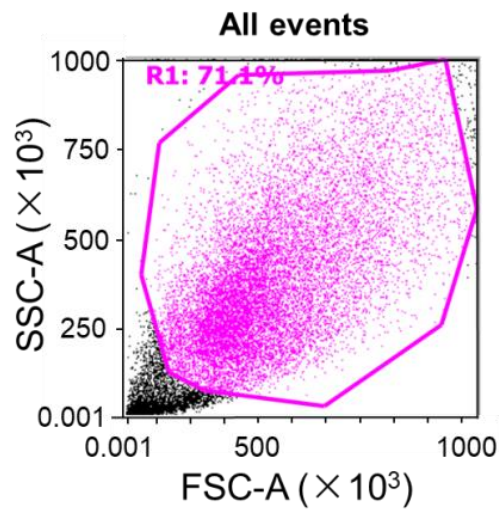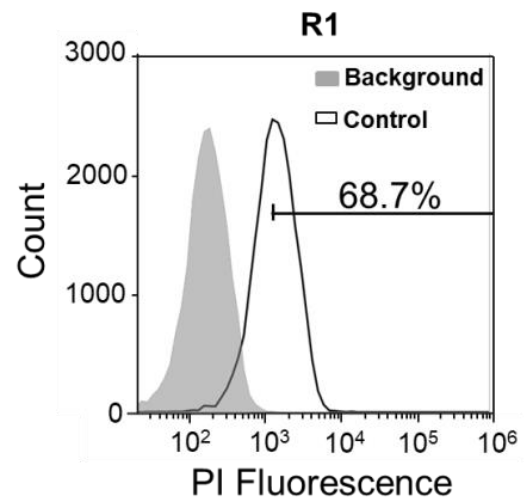

## FUS-Cav

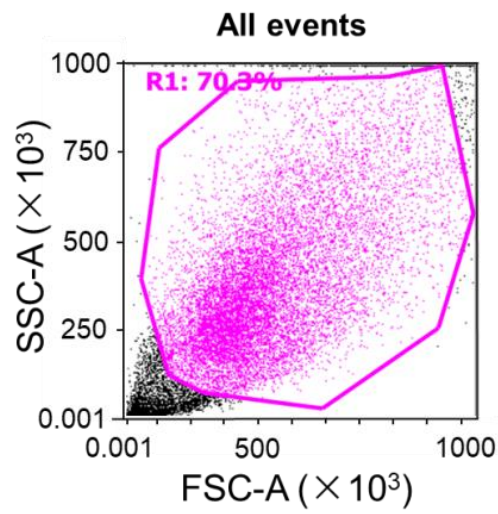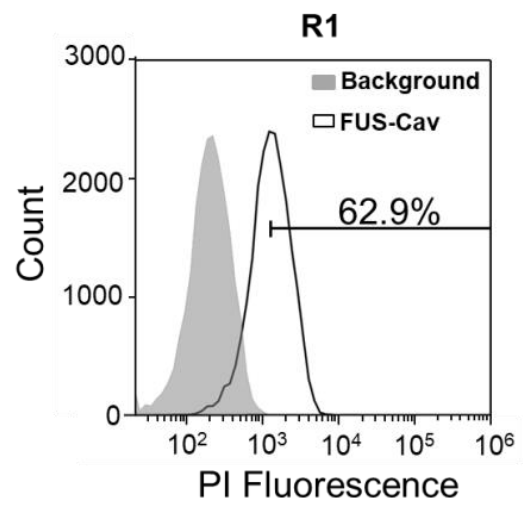

## HT

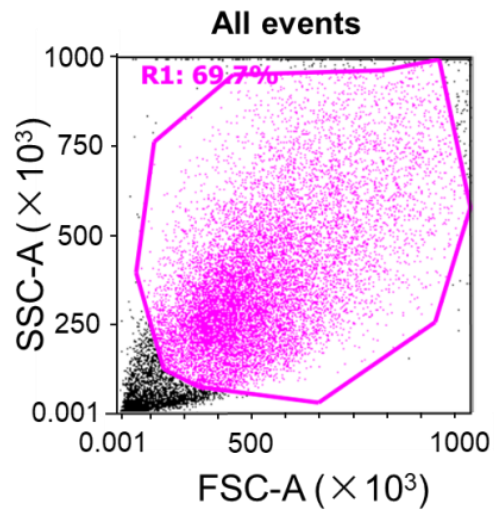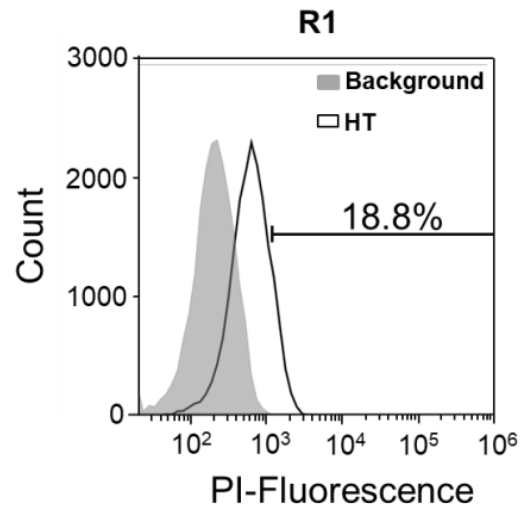

## FUS-Cav + HT

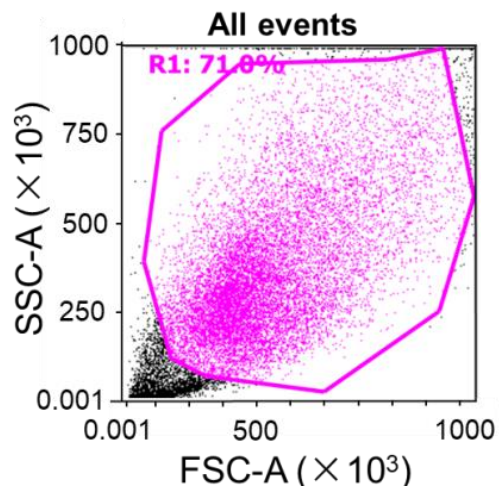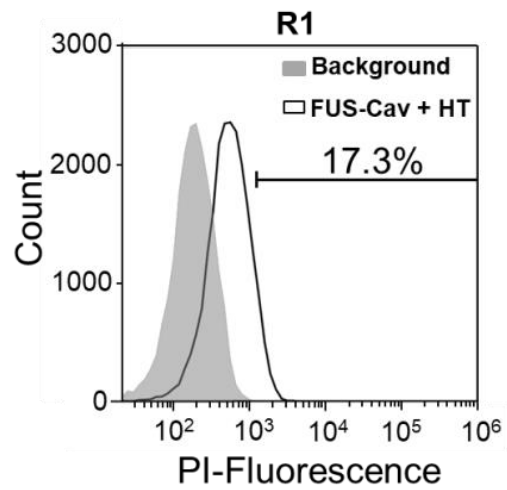

Supplement: Supplementary file 3 [file DataSheet1.ZIP › Figure 6b Flow cytometry results exhibit the percentage of immunofluorescence-positive cells/LNCap SRD5A 3/gating LNCap SRD5A 3.pdf]

## Control

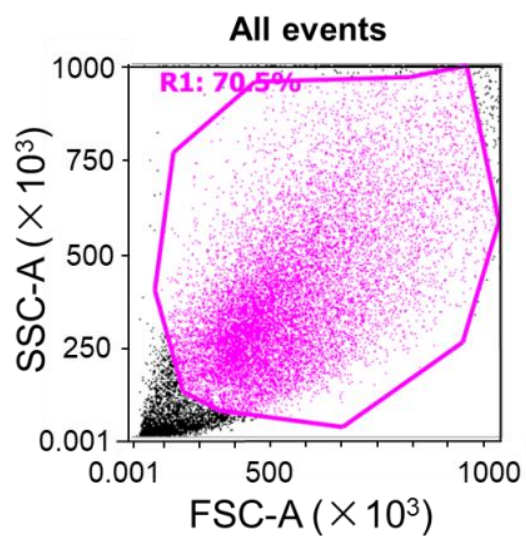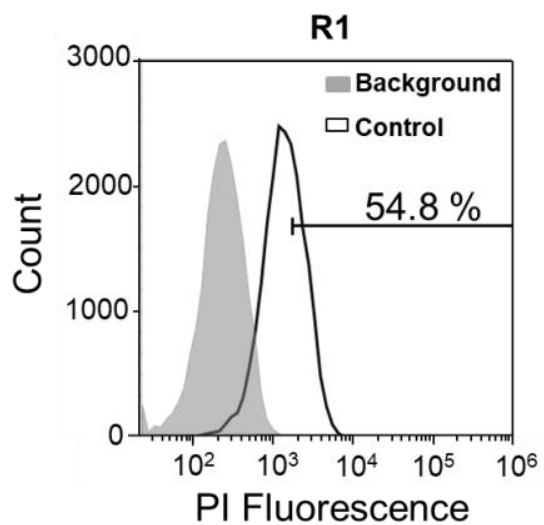

## FUS-Cav

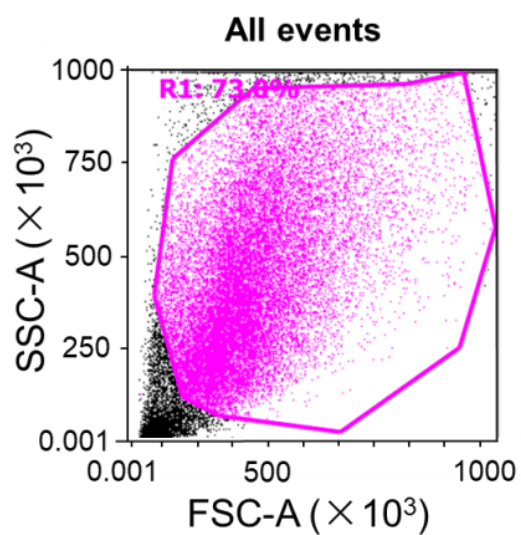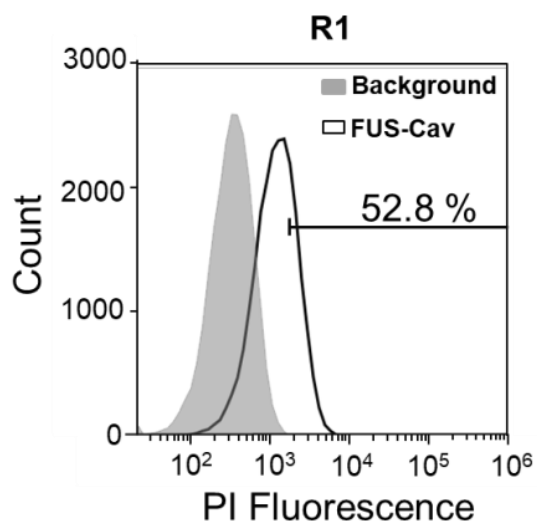

## HT

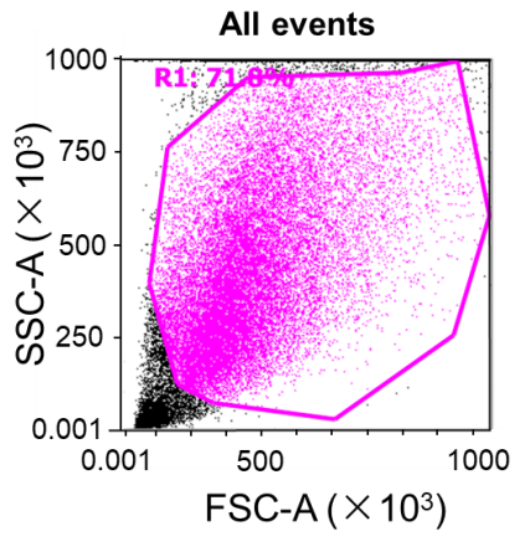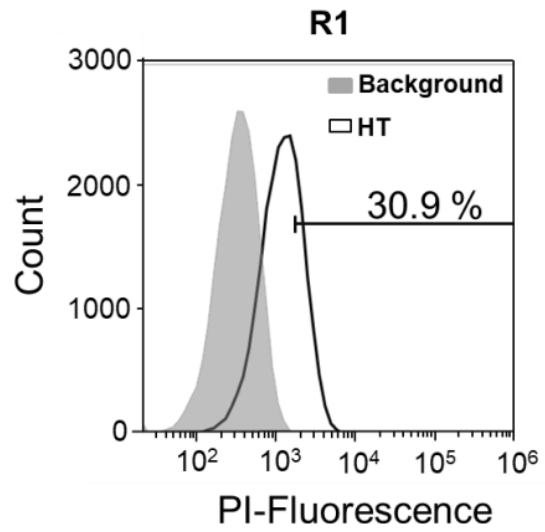

## FUS-Cav + HT

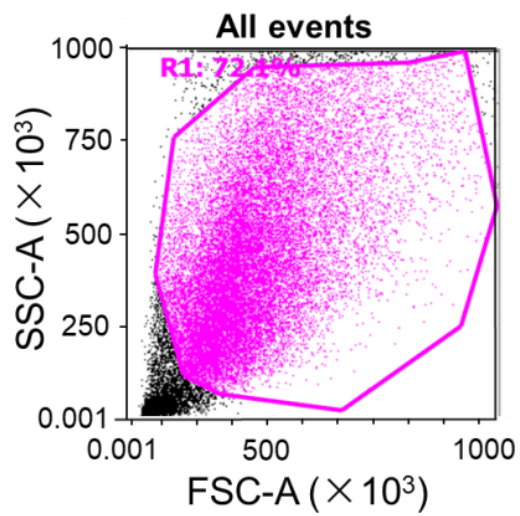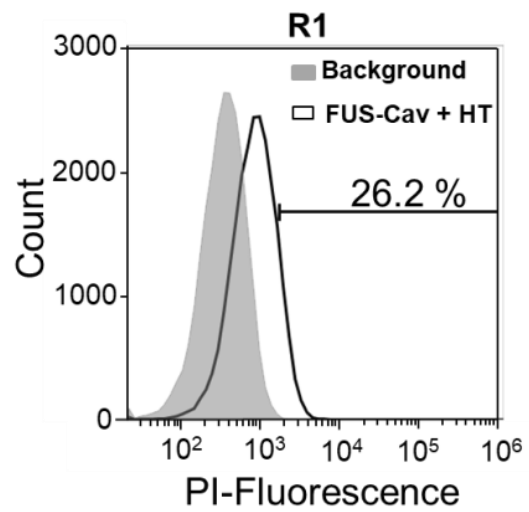

Supplement: Supplementary file 3 [file DataSheet1.ZIP › Figure 6b Flow cytometry results exhibit the percentage of immunofluorescence-positive cells/PC-3 SRD5A 1/gating PC-3 SRD5A 1.pdf]

## Control

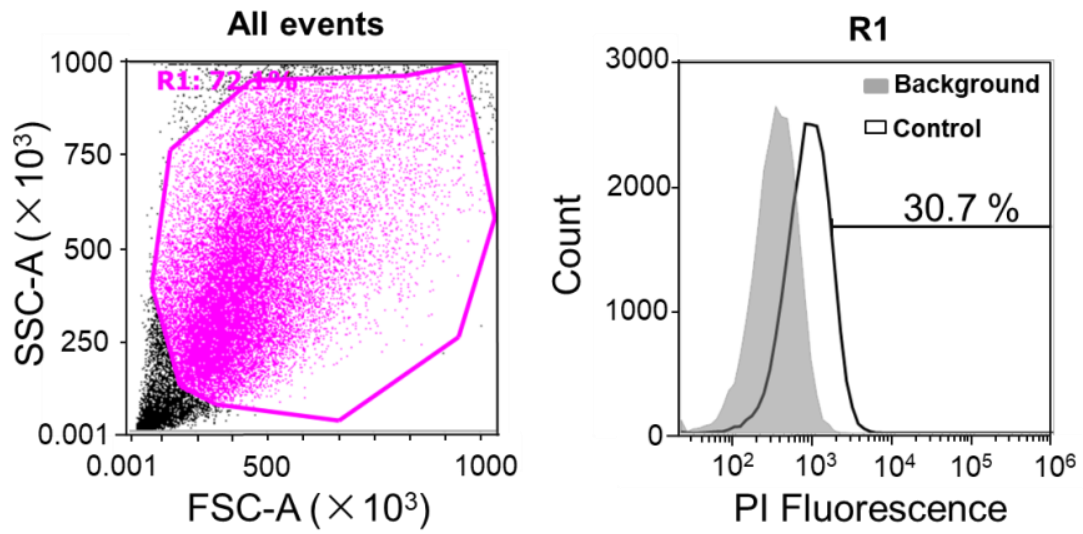

## FUS-Cav

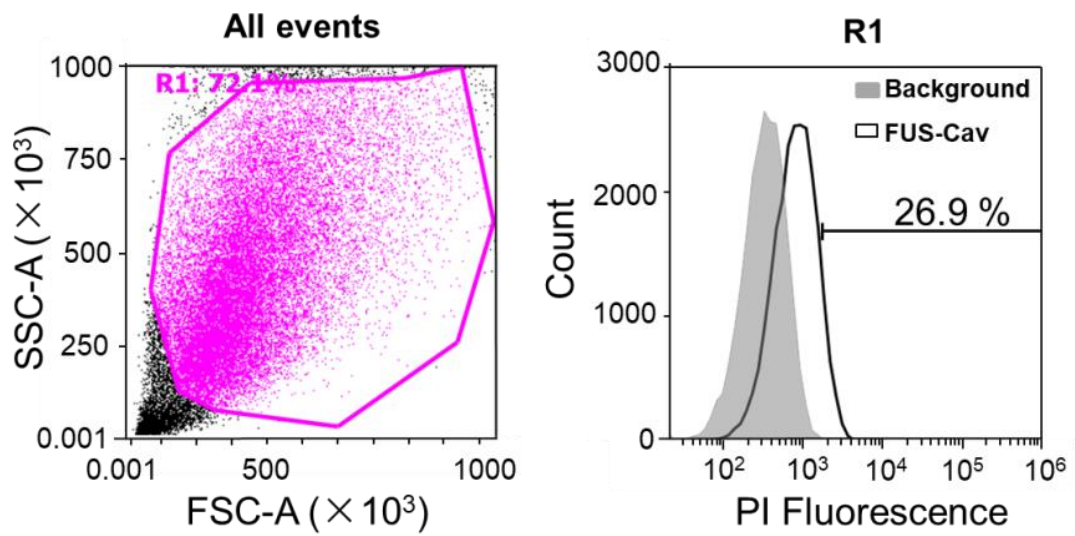

## HT

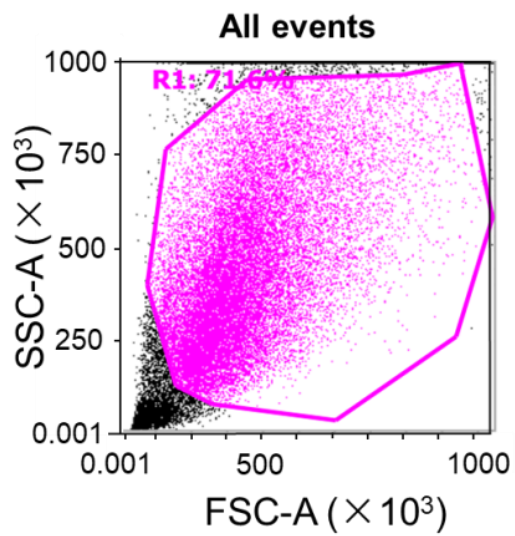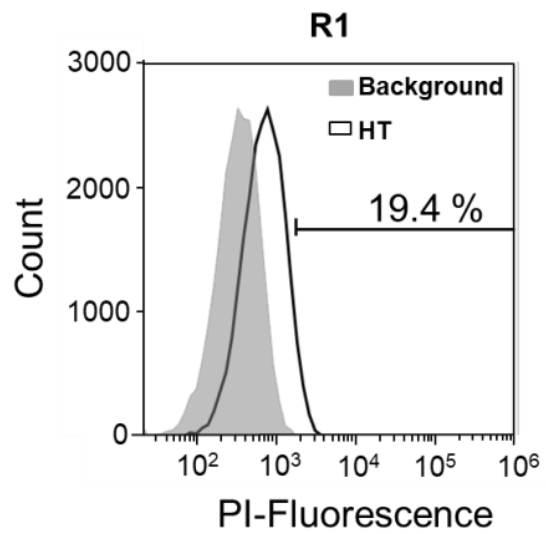

## FUS-Cav + HT

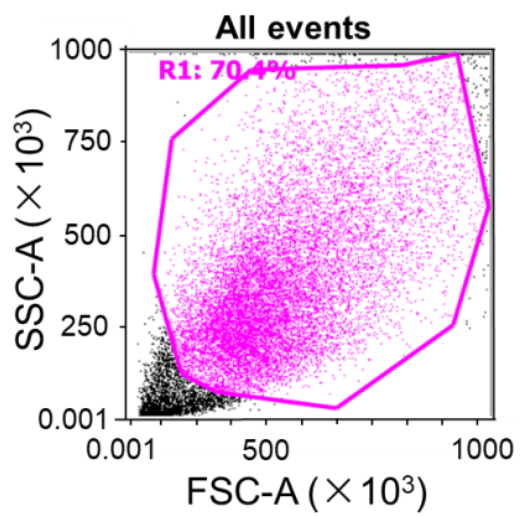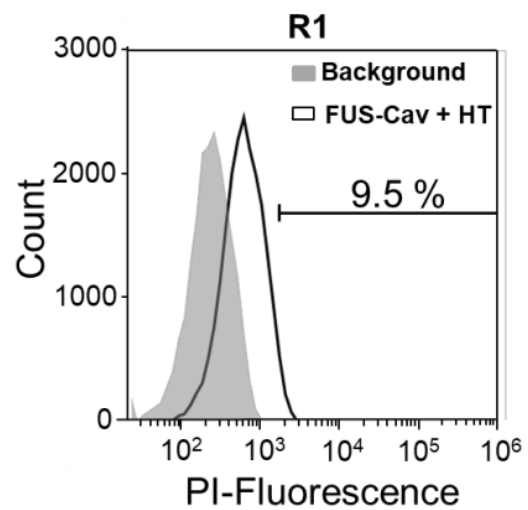

Supplement: Supplementary file 3 [file DataSheet1.ZIP › Figure 6b Flow cytometry results exhibit the percentage of immunofluorescence-positive cells/PC-3 SRD5A 3/gating PC-3 SRD5A 3.pdf]

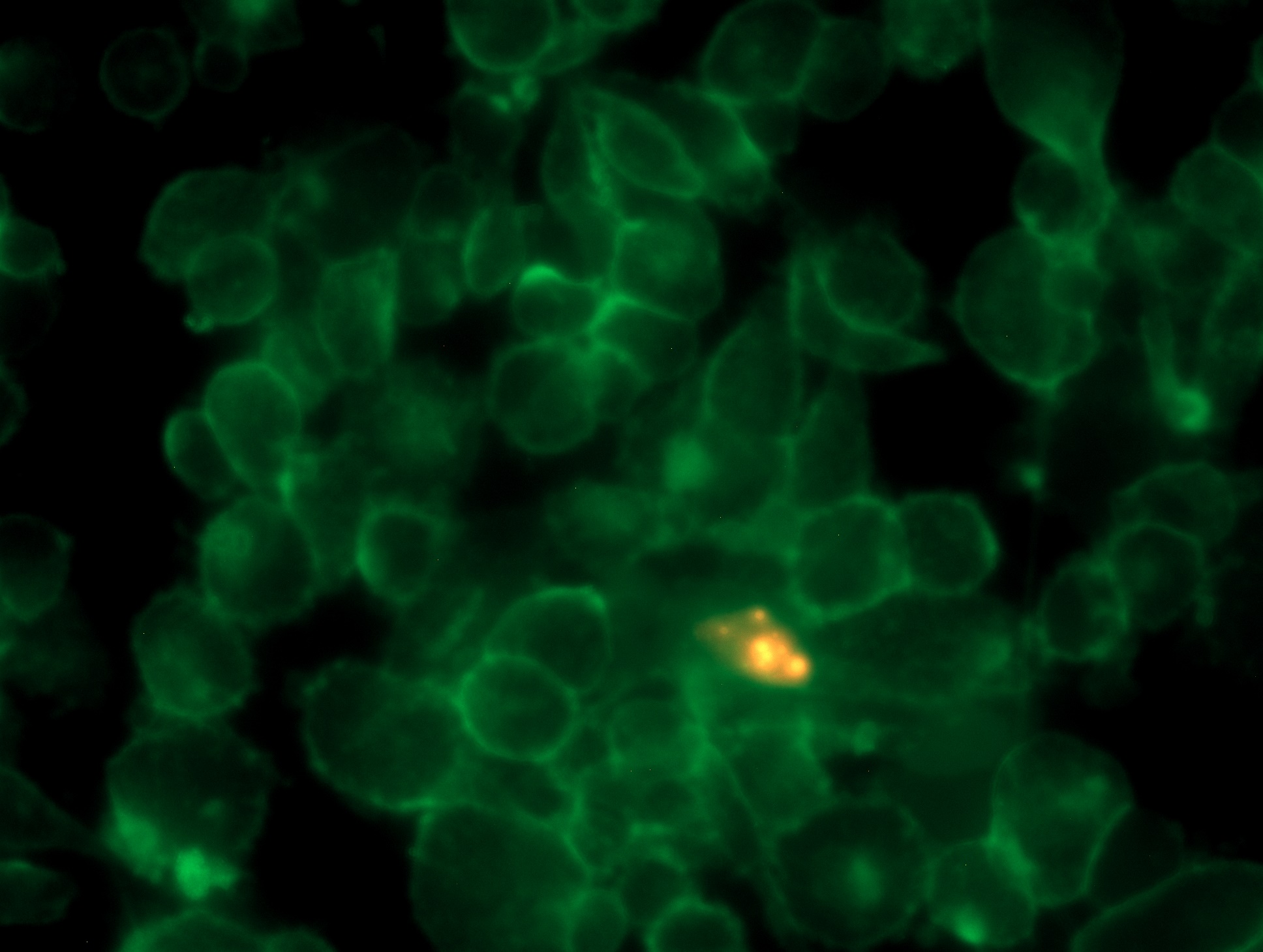

Supplement: Supplementary file 4 [file DataSheet6.ZIP › Figure 7a Representative fluorescence microscopy images for LNCap cells showing sonoporation/30 min after FUS-Cav/Figure 7a Representative fluorescence micros30 min after FUS-Cav merge.jpg]

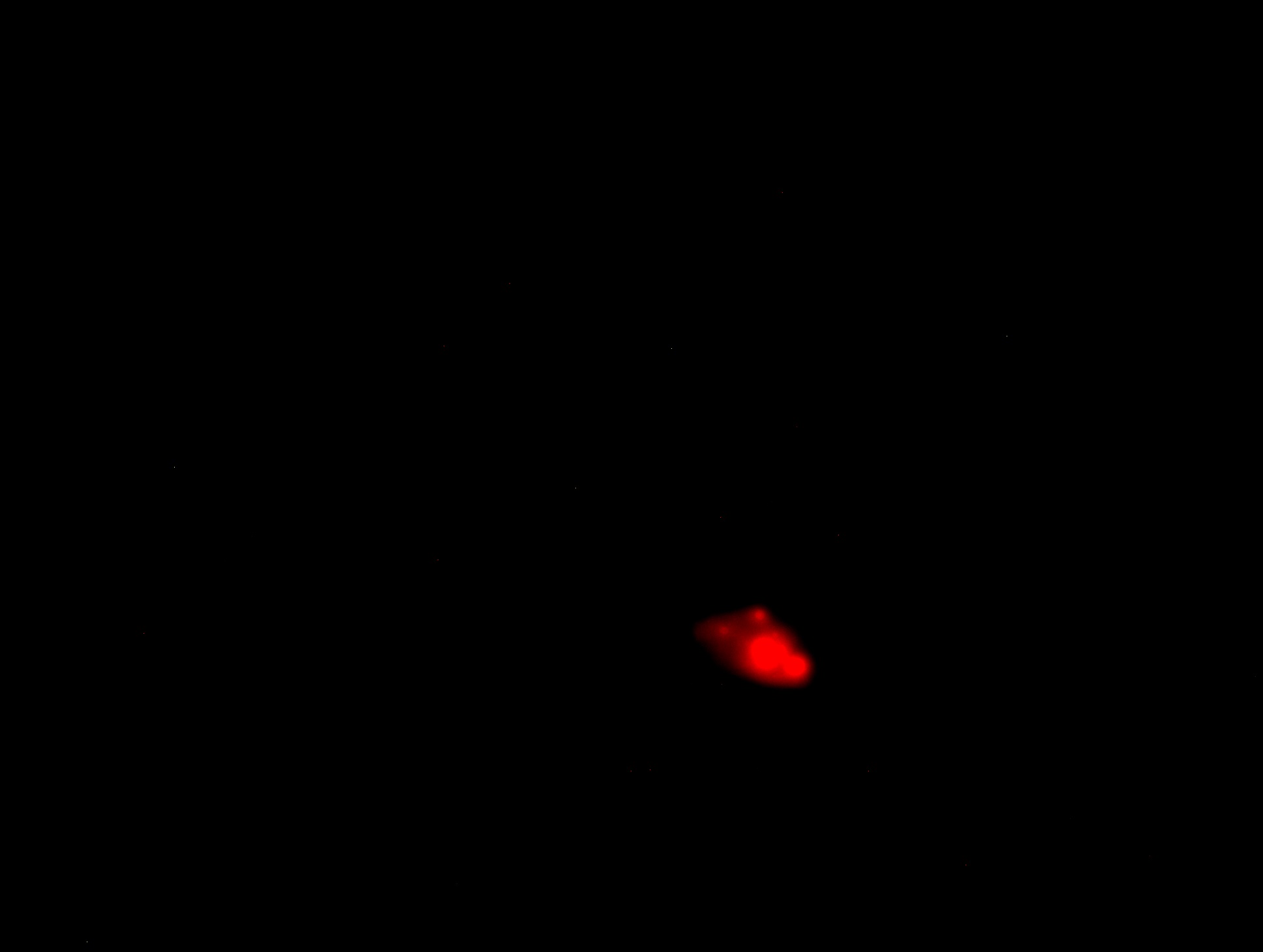

Supplement: Supplementary file 4 [file DataSheet6.ZIP › Figure 7a Representative fluorescence microscopy images for LNCap cells showing sonoporation/30 min after FUS-Cav/Figure 7a Representative fluorescence microscop30 min after FUS-Cav PI.jpg]

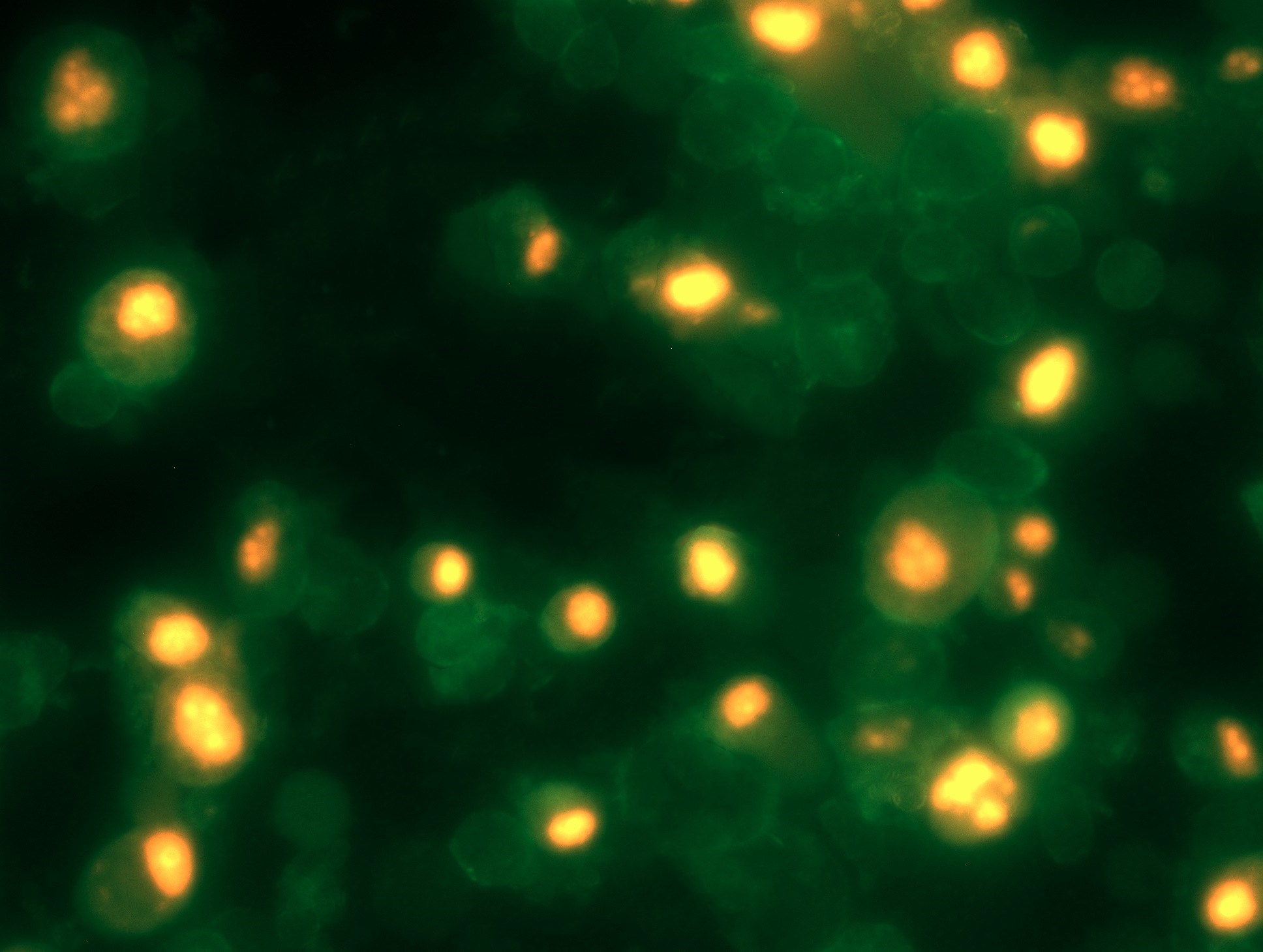

Supplement: Supplementary file 4 [file DataSheet6.ZIP › Figure 7a Representative fluorescence microscopy images for LNCap cells showing sonoporation/FUS-Cav/Figure 7a Representative fluorescence microscopy images for LNCap cellFUS-Cav merge.jpg]

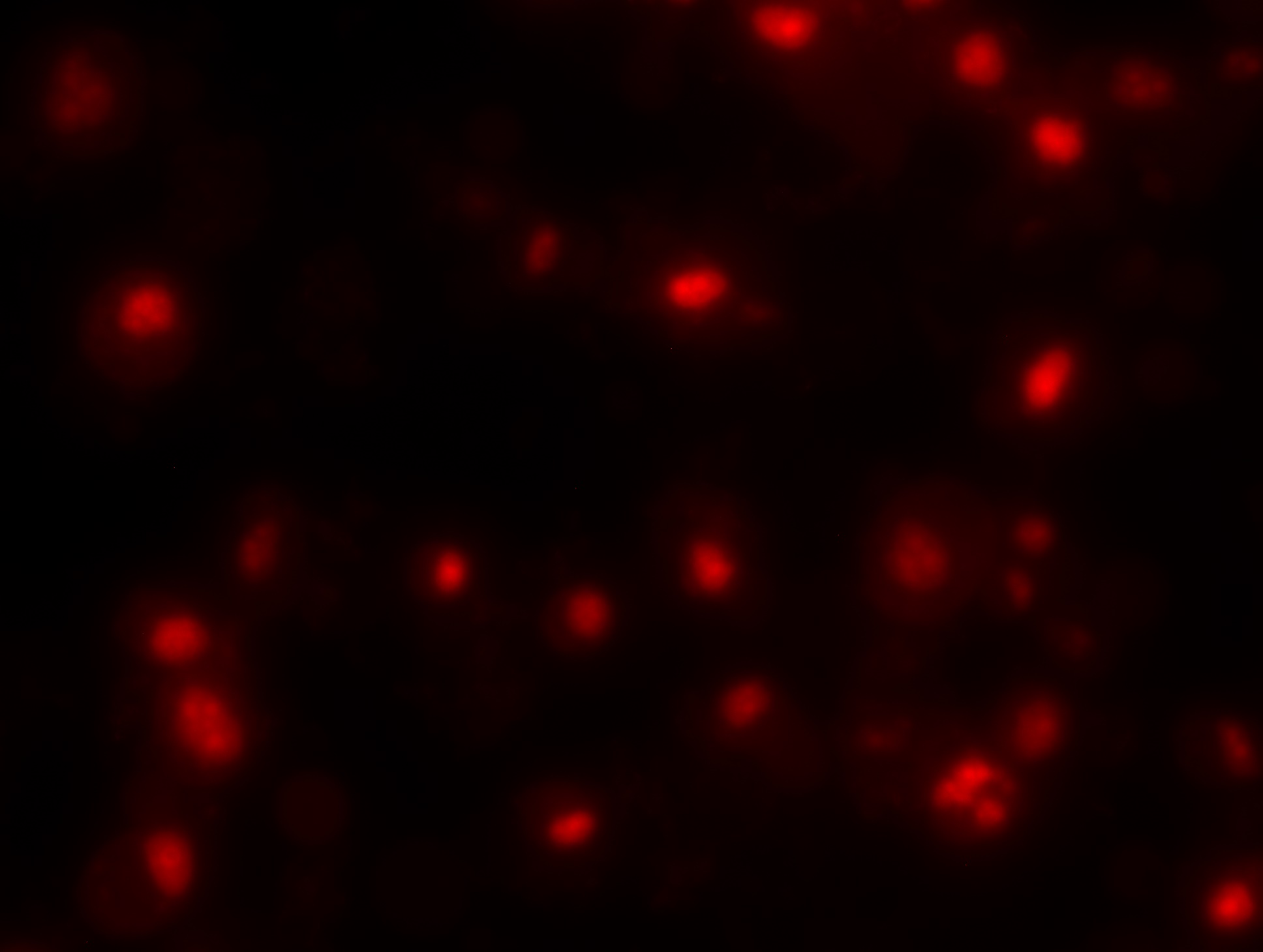

Supplement: Supplementary file 4 [file DataSheet6.ZIP › Figure 7a Representative fluorescence microscopy images for LNCap cells showing sonoporation/FUS-Cav/Figure 7a Representative fluorescence microscopy images for LNCap cells sFUS-Cav PI.jpg]

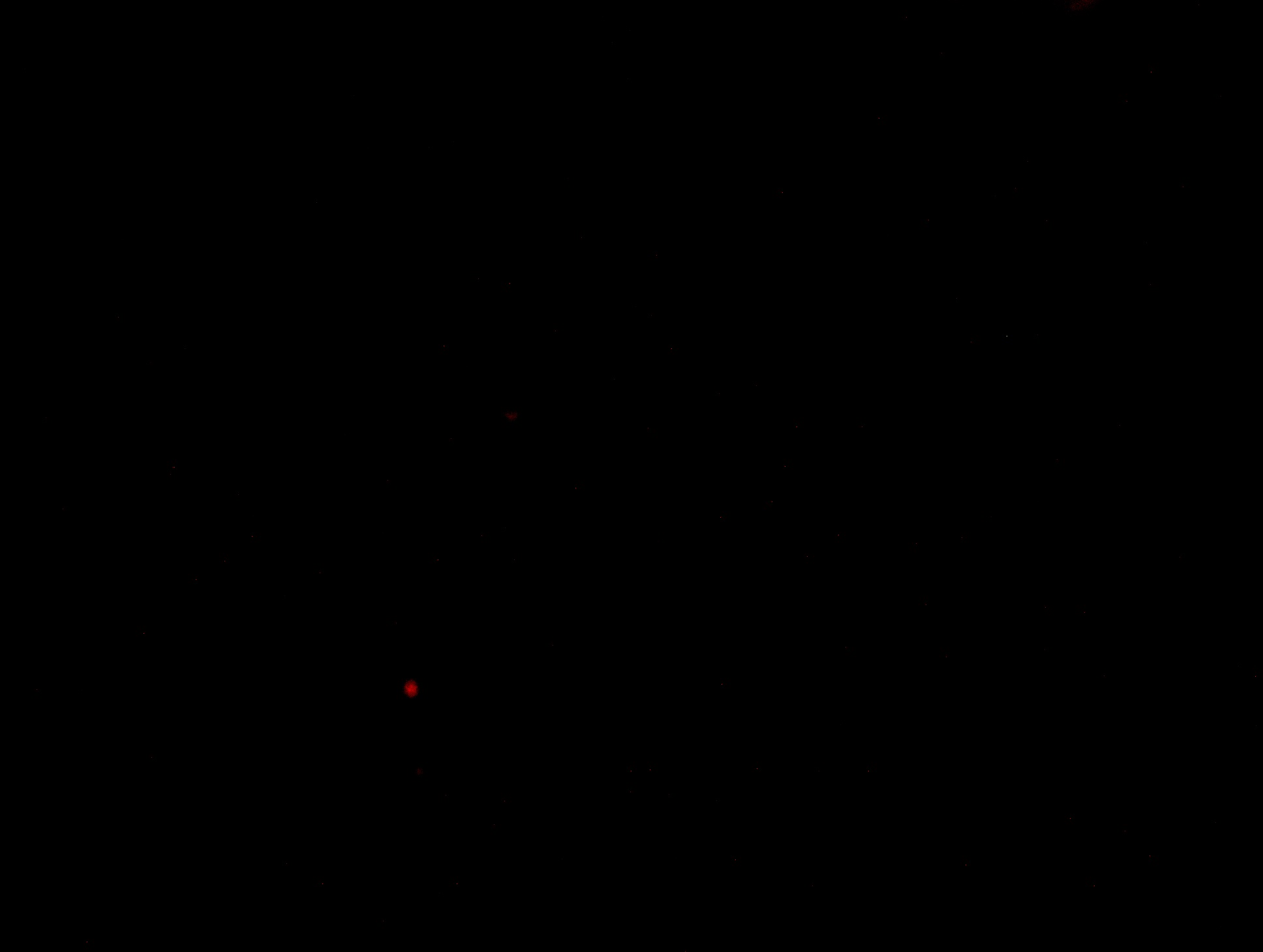

Supplement: Supplementary file 4 [file DataSheet6.ZIP › Figure 7a Representative fluorescence microscopy images for LNCap cells showing sonoporation/Utreated control/Figure 7a Representative fluorescence microscopy images foUtreated control.jpg]

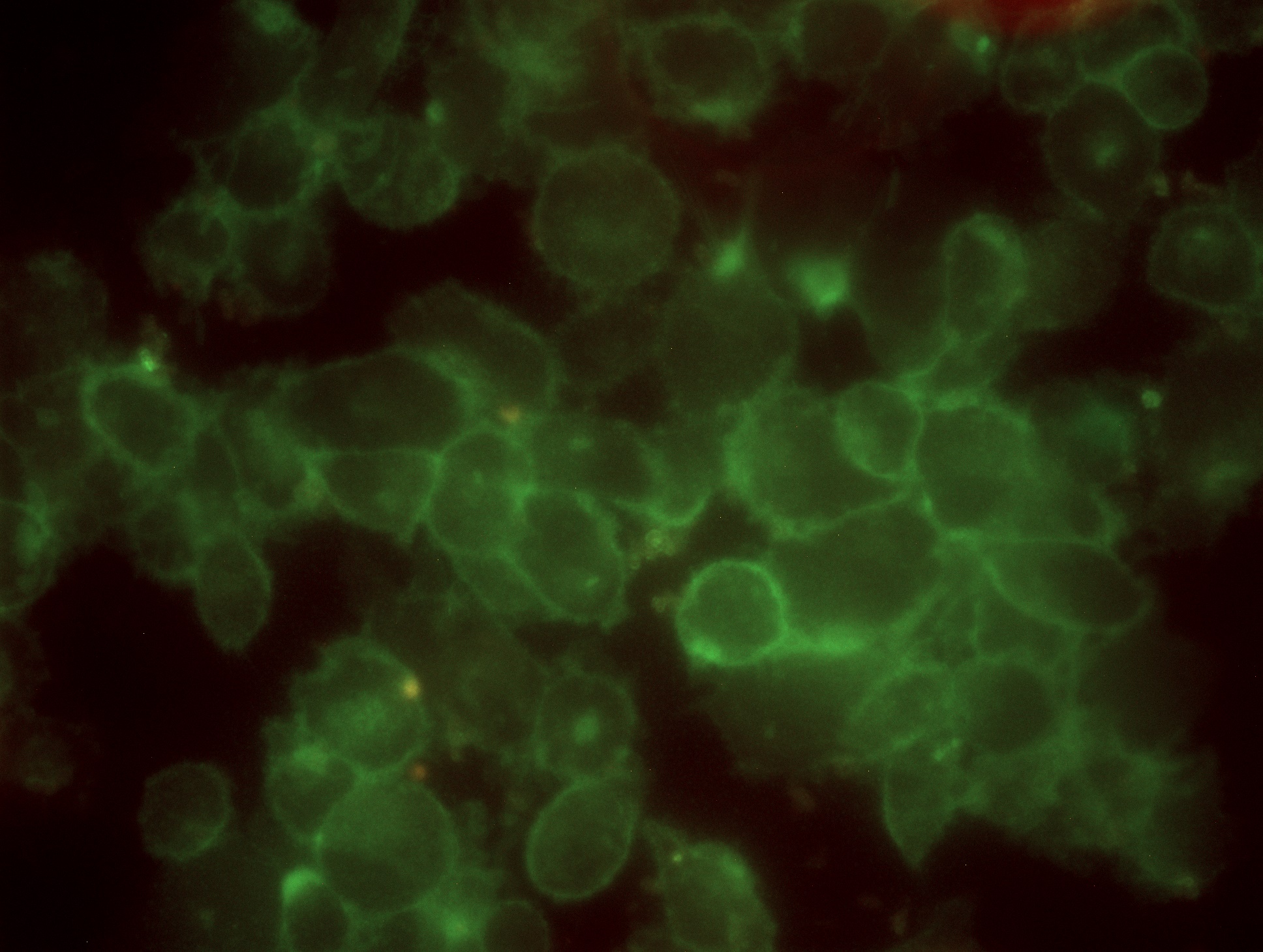

Supplement: Supplementary file 4 [file DataSheet6.ZIP › Figure 7a Representative fluorescence microscopy images for LNCap cells showing sonoporation/Utreated control/Figure 7a Representative fluorescence microscopy imaUtreated control merge.jpg]

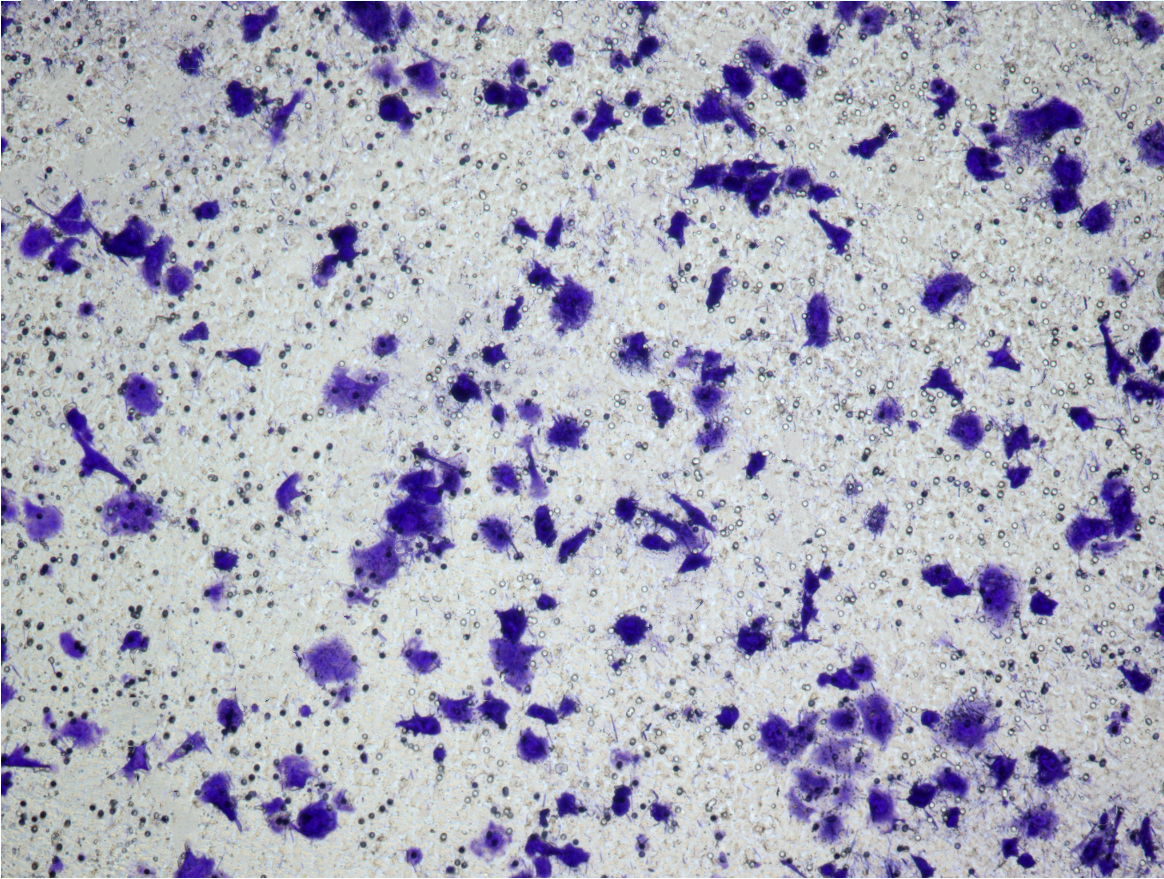

Supplement: Supplementary file 5 [file DataSheet2.ZIP › Figure 3Ba Microscopy images for LNCap cell invasion/Figure 3Ba Microscopy images for LNCap cell invasion Control.tif]

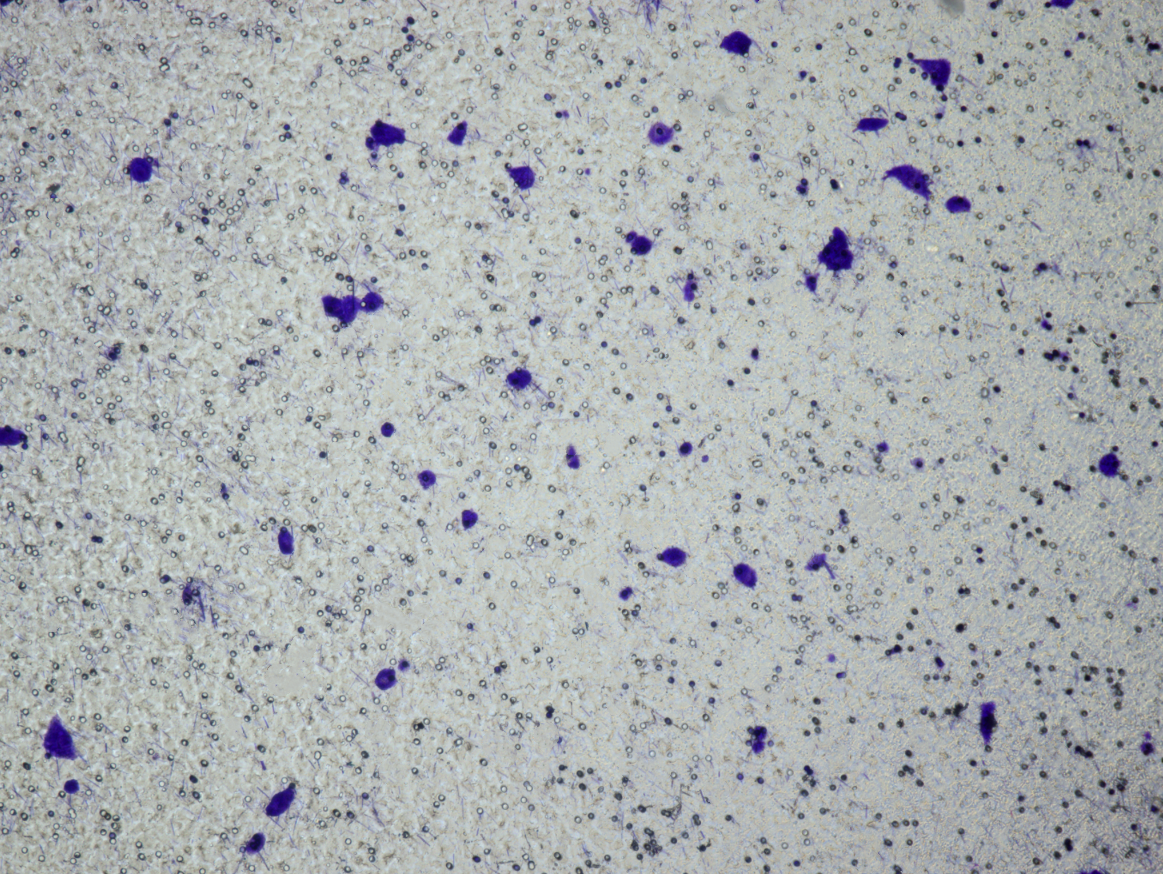

Supplement: Supplementary file 5 [file DataSheet2.ZIP › Figure 3Ba Microscopy images for LNCap cell invasion/Figure 3Ba Microscopy images for LNCap cell invasion FUS-Cav+HT.tif]

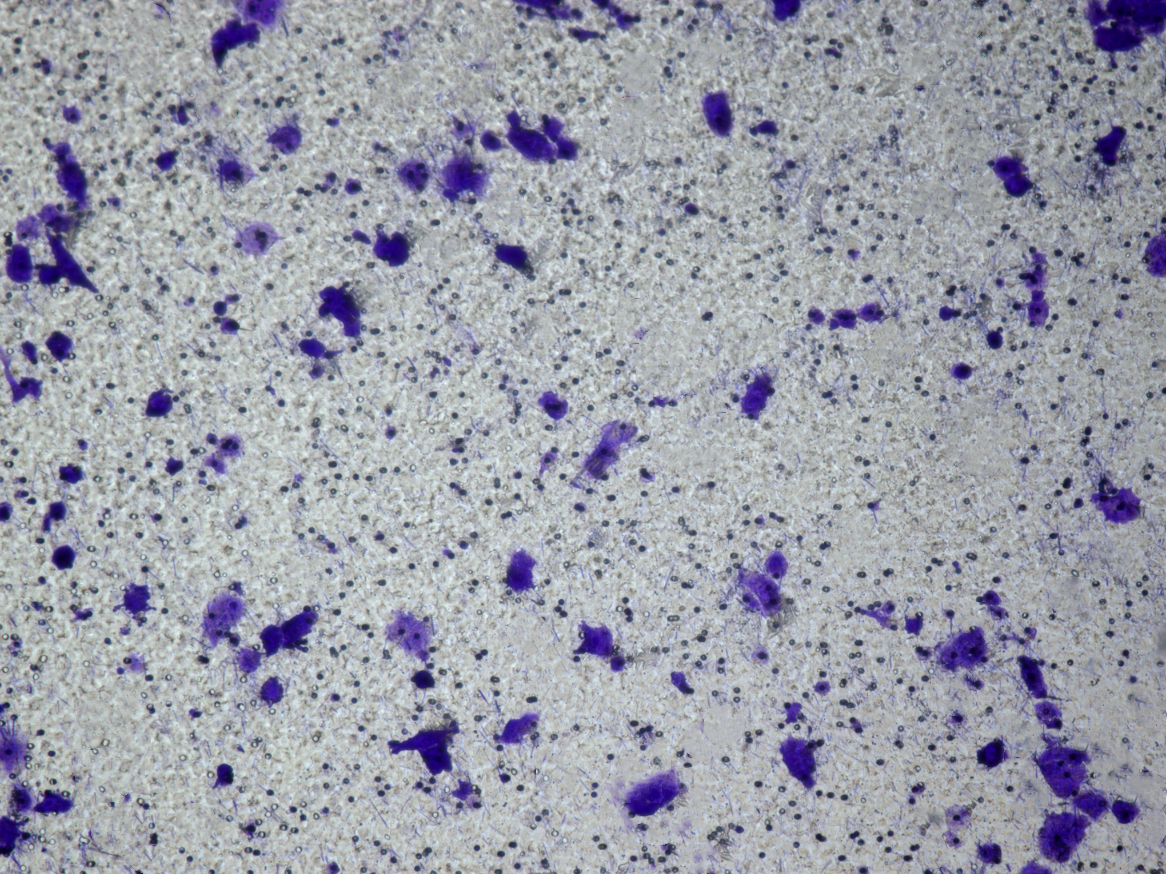

Supplement: Supplementary file 5 [file DataSheet2.ZIP › Figure 3Ba Microscopy images for LNCap cell invasion/Figure 3Ba Microscopy images for LNCap cell invasion HT.tif]

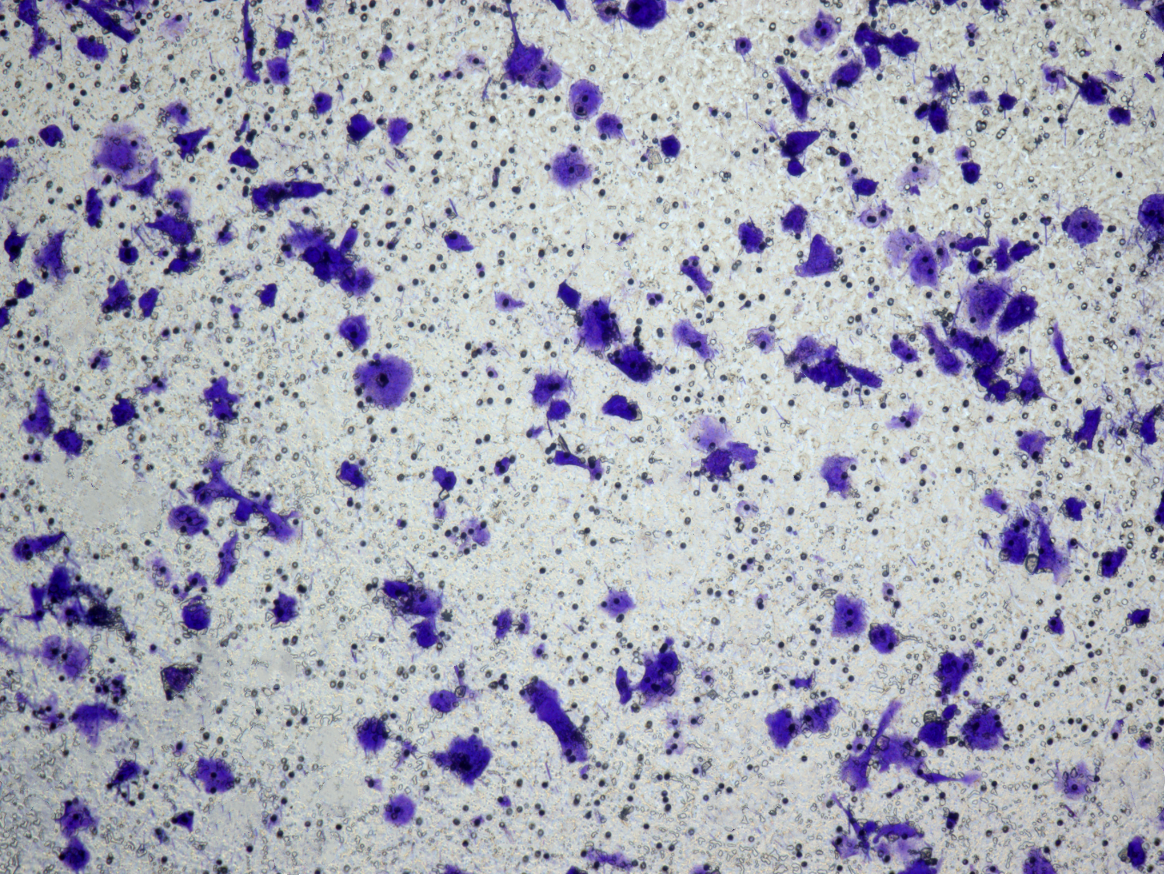

Supplement: Supplementary file 5 [file DataSheet2.ZIP › Figure 3Ba Microscopy images for LNCap cell invasion/Figure 3Ba Microscopy images for LNCap cell invasionFUS-Cav.tif]

## Slide 1
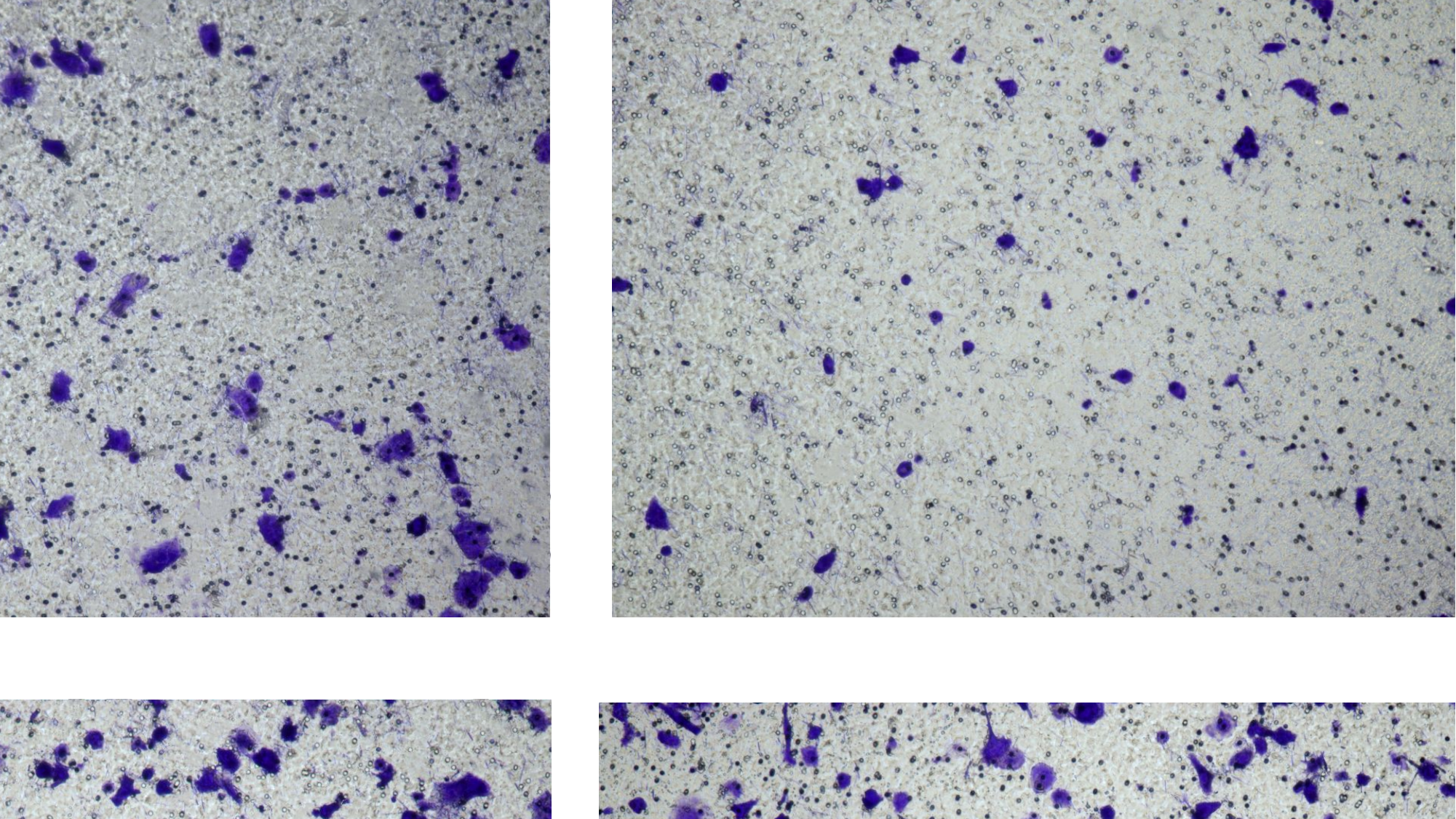

Supplement: Supplementary file 5 [file DataSheet2.ZIP › Figure 3Ba Microscopy images for LNCap cell invasion/╨┬╜¿ Microsoft PowerPoint ╤▌╩╛╬─╕σ.pptx]

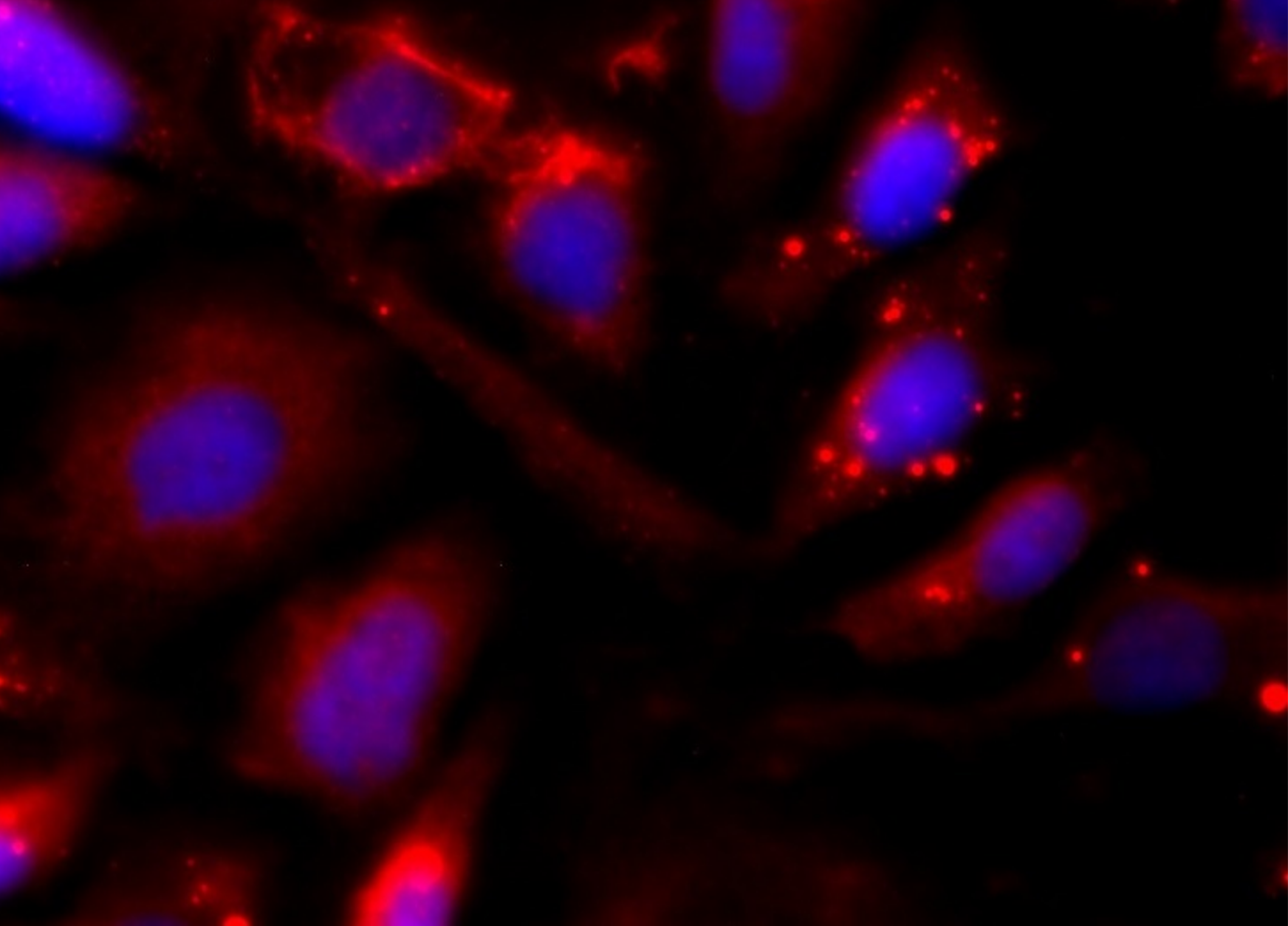

Supplement: Supplementary file 6 [file DataSheet5.ZIP › PC-3 Control SRD5A 1.tif]

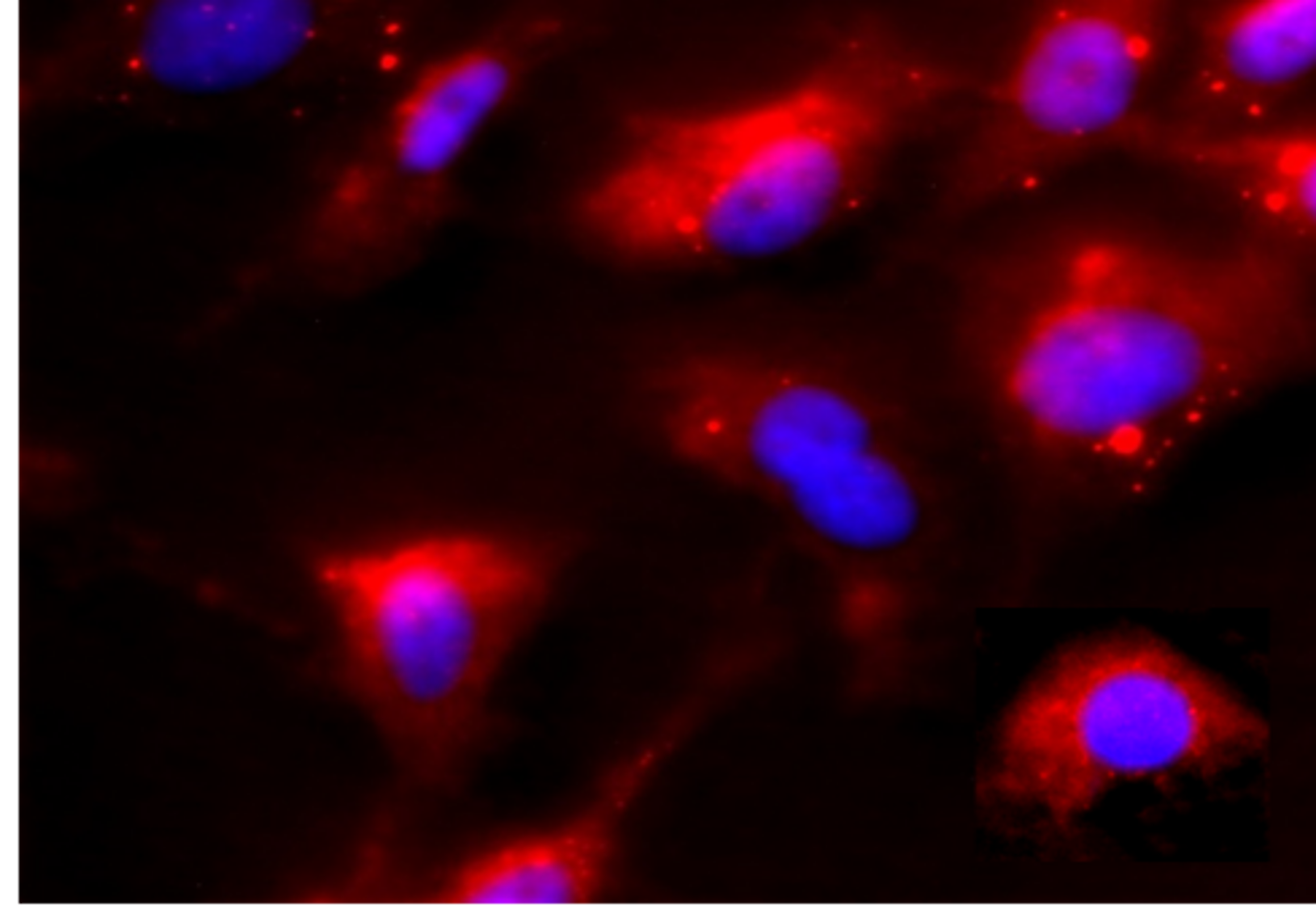

Supplement: Supplementary file 6 [file DataSheet5.ZIP › PC-3 Control SRD5A3.tif]

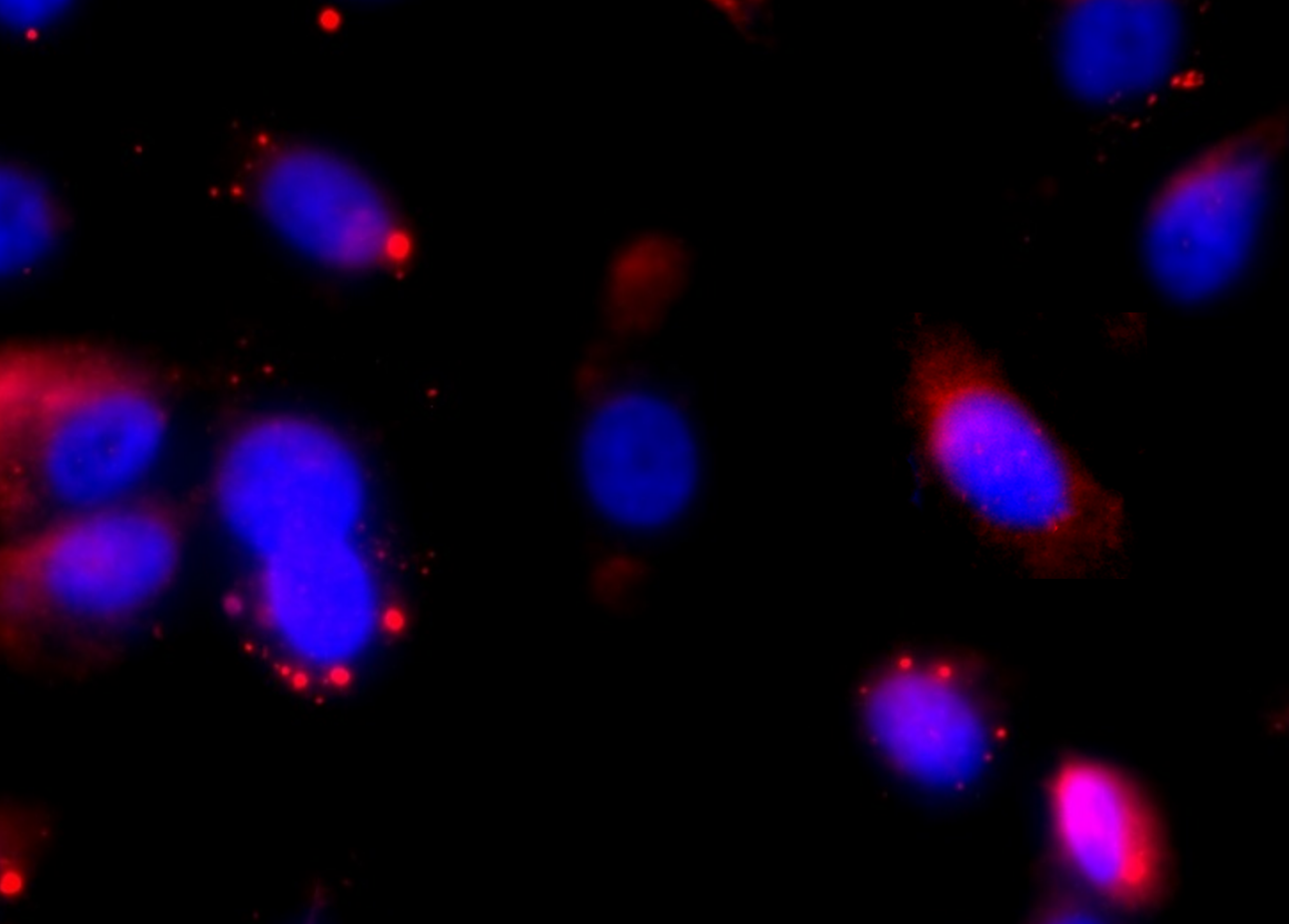

Supplement: Supplementary file 6 [file DataSheet5.ZIP › PC-3 FUS-Cav + HT SRD5A 1.tif]

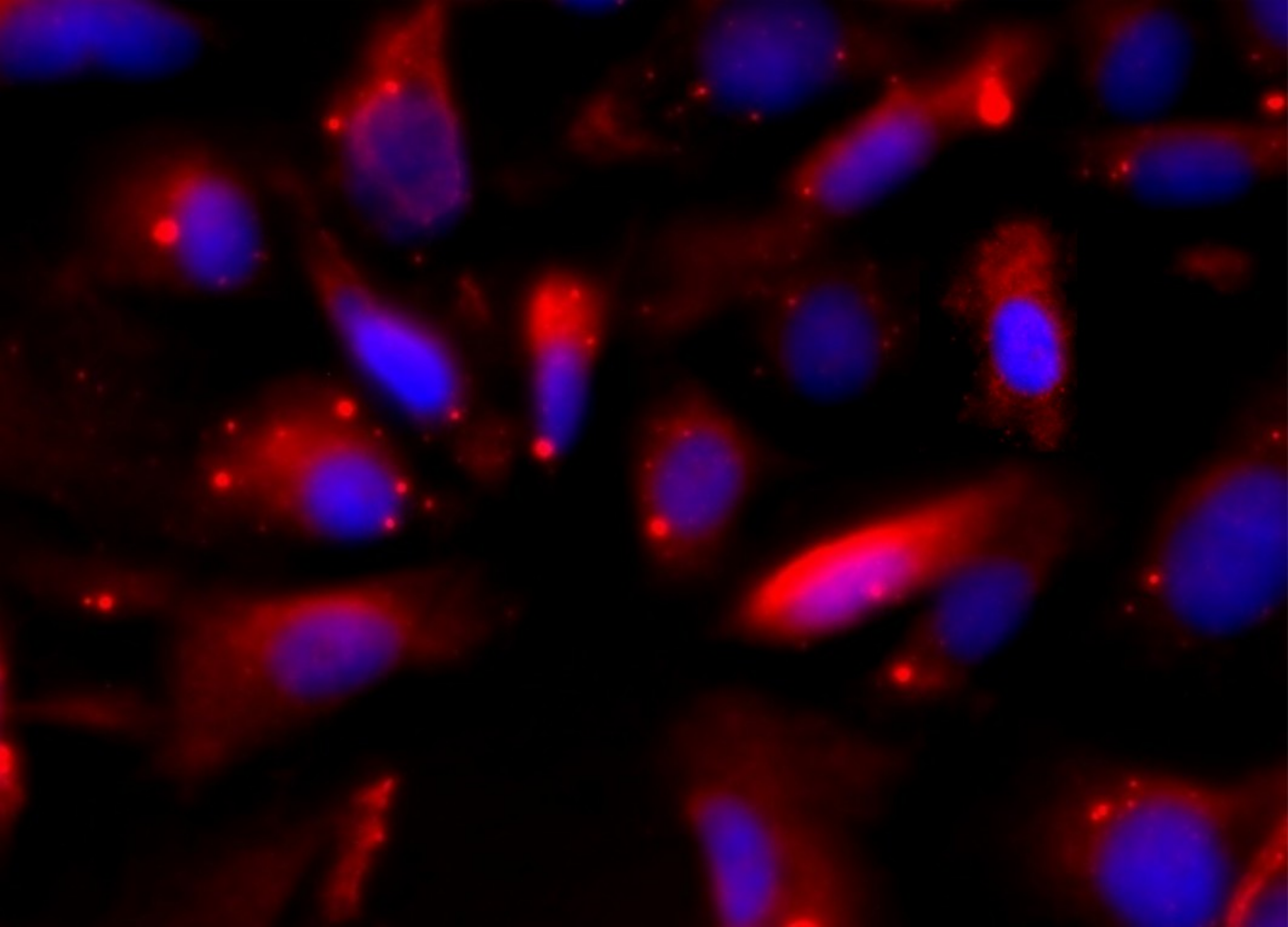

Supplement: Supplementary file 6 [file DataSheet5.ZIP › PC-3 FUS-Cav SRD5A 1.tif]

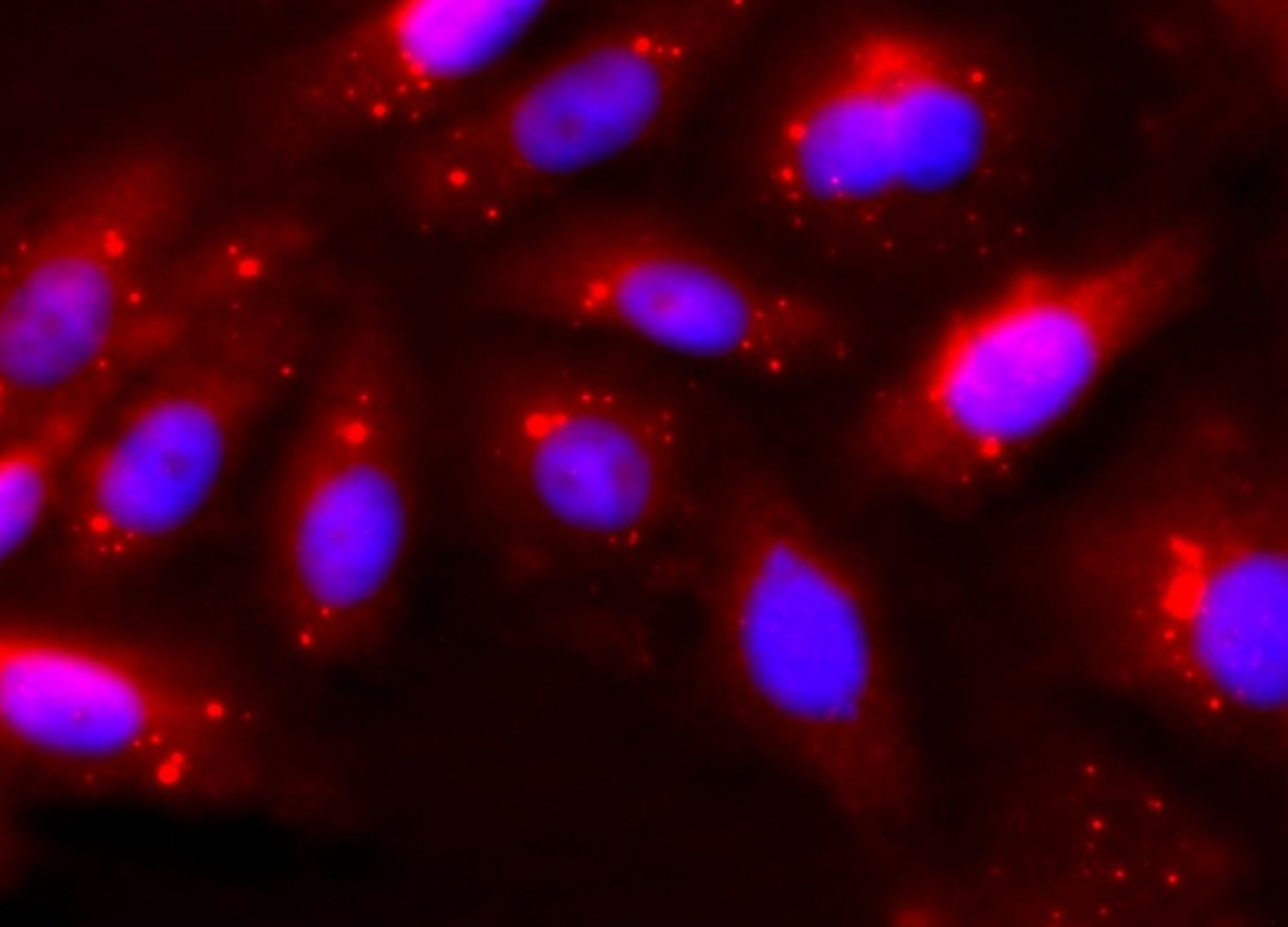

Supplement: Supplementary file 6 [file DataSheet5.ZIP › PC-3 FUS-Cav SRD5A3.tif]

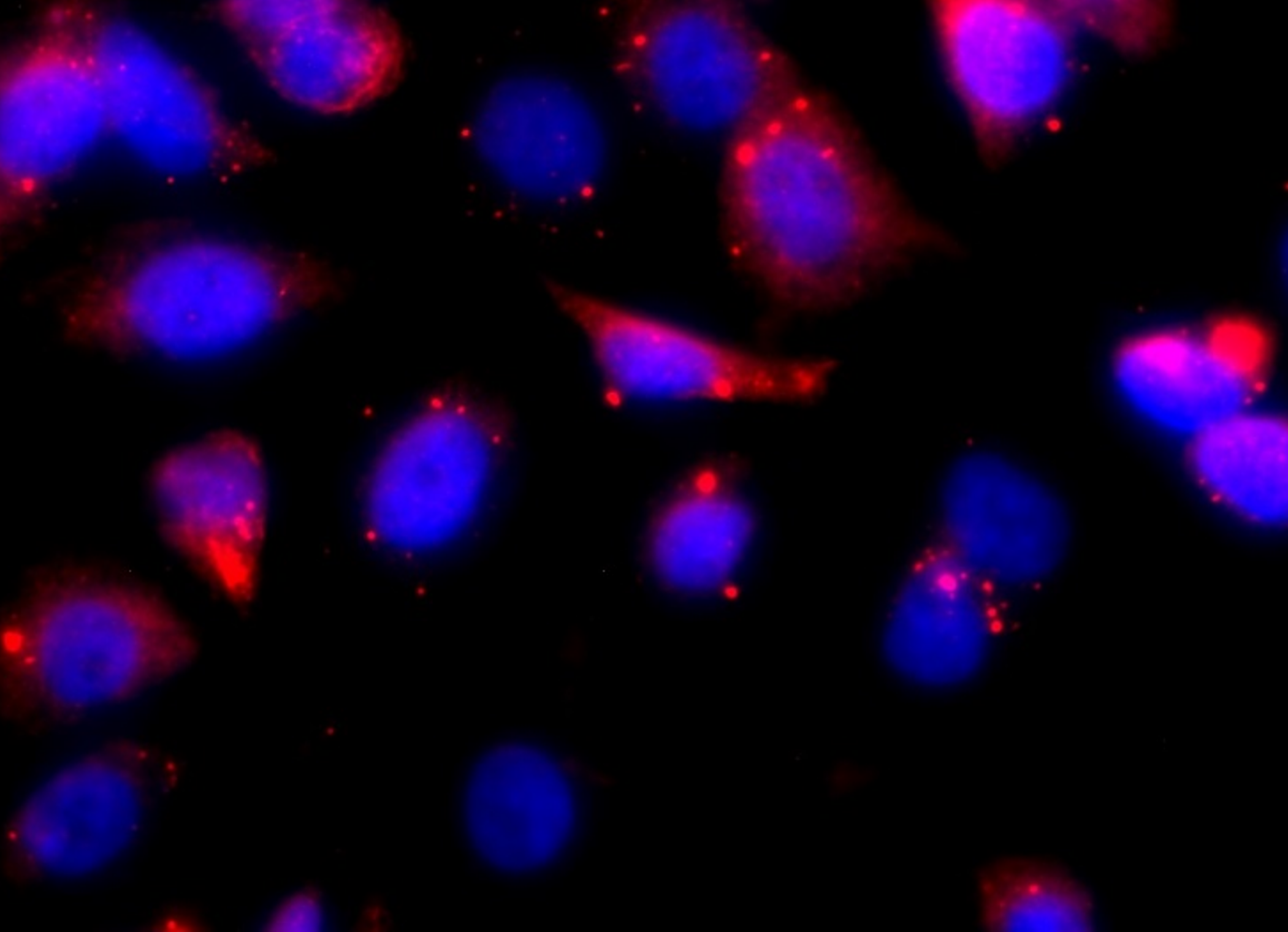

Supplement: Supplementary file 6 [file DataSheet5.ZIP › PC-3 FUS-Cav+HT SRD5A3.tif]

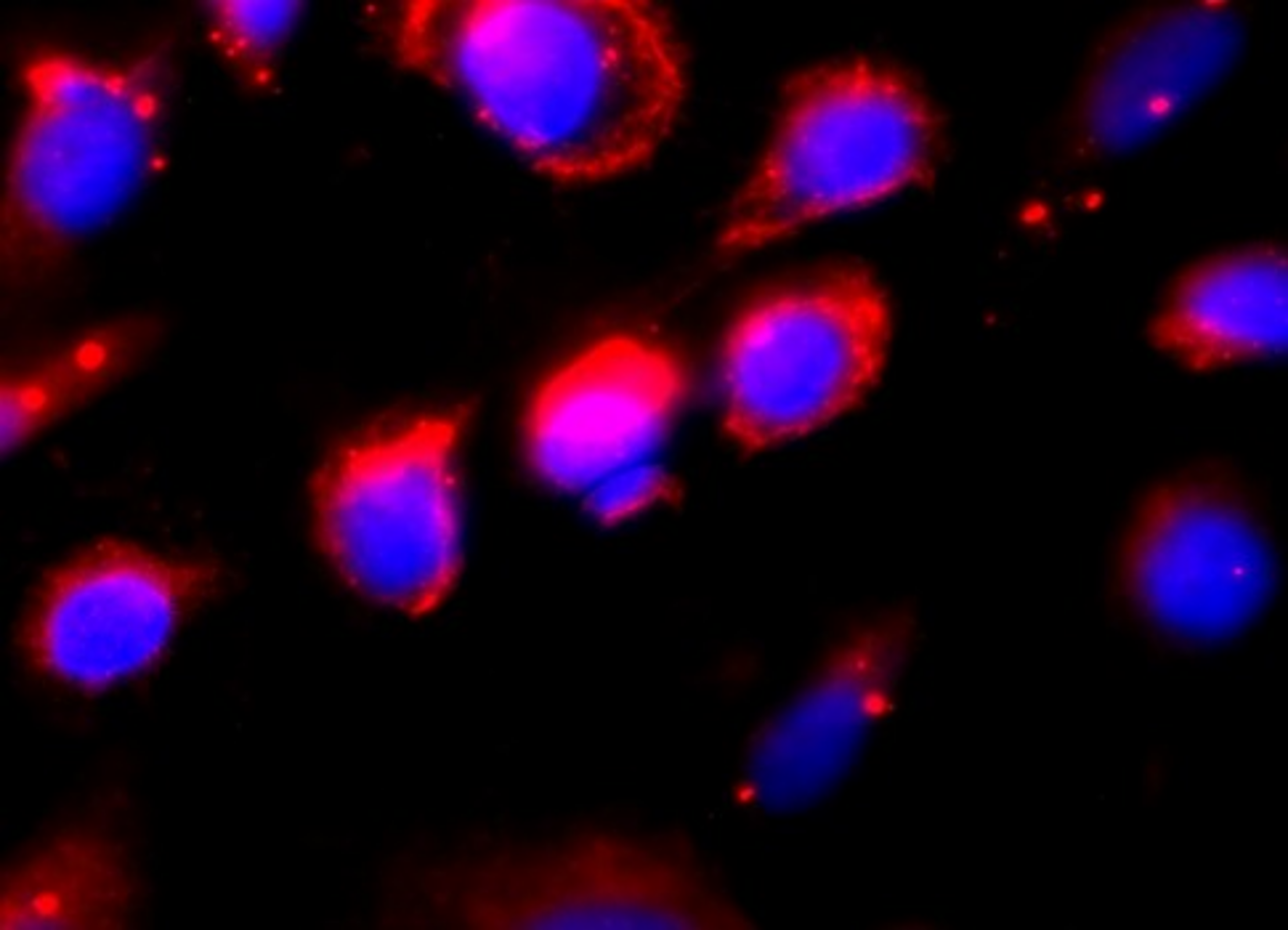

Supplement: Supplementary file 6 [file DataSheet5.ZIP › PC-3 HT SRD5A 1.tif]

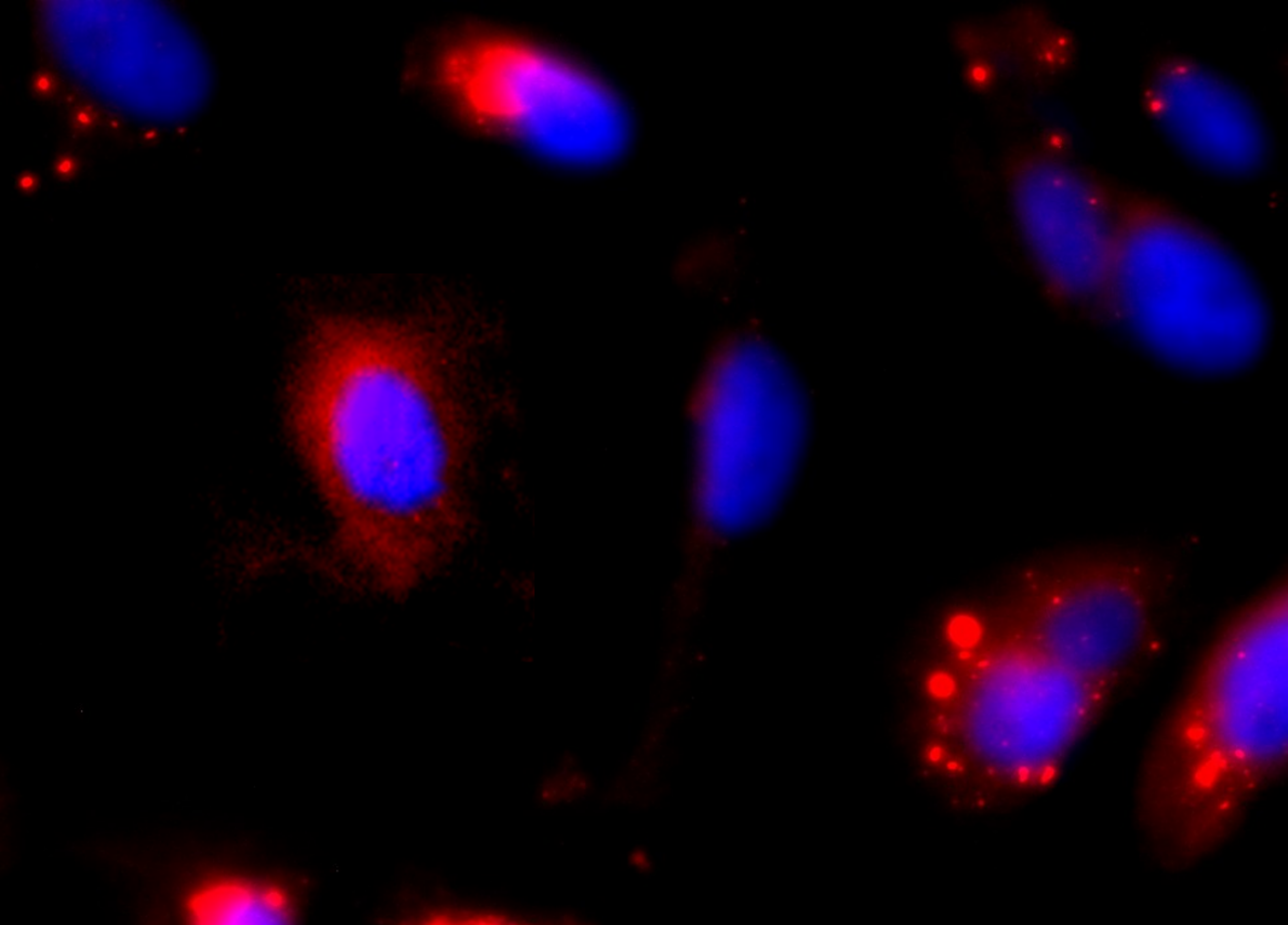

Supplement: Supplementary file 6 [file DataSheet5.ZIP › PC-3 HT SRD5A3.tif]
